# Cosmogenic ^10^Be and equilibrium-line altitude dataset of Holocene glacier advances in the Himalayan-Tibetan orogen

**DOI:** 10.1016/j.dib.2019.104412

**Published:** 2019-08-20

**Authors:** Sourav Saha, Lewis A. Owen, Elizabeth N. Orr, Marc W. Caffee

**Affiliations:** a Department of Earth, Planetary and Space Sciences, University of California, Los Angeles, CA, 90095, USA; b Department of Physics, Geology, and Engineering Technology, Northern Kentucky University, KY, 41099, USA; c Department of Marine, Earth, and Atmospheric Sciences, North Carolina State University, Raleigh, NC, 27695, USA; d German Research Centre for Geosciences (GFZ), University of Potsdam, Institute for Geosciences, 14476, Potsdam, Germany; e Department of Physics, Department of Earth, Atmospheric and Planetary Sciences, Purdue University, West Lafayette, IN, 47907, USA

**Keywords:** Cosmogenic nuclides, Equilibrium-line altitudes, Holocene, Central asia, Glaciation

## Abstract

A comprehensive analysis of the variable temporal and spatial responses of tropical-subtropical high-altitude glaciers to climate change is critical for successful model predictions and environmental risk assessment in the Himalayan-Tibetan orogen. High-frequency Holocene glacier chronostratigraphies are therefore reconstructed in 79 glaciated valleys across the orogen using 519 published and 16 new terrestrial cosmogenic ^10^Be exposure age dataset. Published ^10^Be ages are compiled only for moraine boulders (excluding bedrock ages). These ages are recalculated using the latest ICE-D production rate calibration database and the scaling scheme models. Outliers for the individual moraine are detected using the Chauvenet's criterion. In addition, past equilibrium-line altitudes (ELAs) are determined using the area-altitude (AA), area accumulation ratio (AAR), and toe-headwall accumulation ratio (THAR) methods for each glacier advance. The modern maximum elevations of lateral moraines (MELM) are also used to estimate modern ELAs and as an independent check on mean ELAs derived using the above three methods. These data may serve as an essential archive for future studies focusing on the cryospheric and environmental changes in the Himalayan-Tibetan orogen. A more comprehensive analysis of the published and new ^10^Be ages and ELA results and a list of references are presented in Saha et al. (2019, High-frequency Holocene glacier fluctuations in the Himalayan-Tibetan orogen. Quaternary Science Reviews, 220, 372–400).

**Table:** Specifications table

Subject	Earth and Planetary Sciences
Specific subject area	Earth-Surface Processes; Geology.
Type of data	Table
How data were acquired	Accelerated mass spectrometry (AMS); Google Earth imagery; Advanced Spaceborne Thermal Emission and Reflection Radiometer (ASTER) global digital elevation models (GDEMs) V2; Landsat Enhanced Thematic Mapper Plus (ETM+). ArcGIS 10.5; Read ArcGrid; MATLAB, R *‘luminescence’* statistical package; https://crep.otelo.univ-lorraine.fr/#/; http://hess.ess.washington.edu/; http://cronus.cosmogenicnuclides.rocks/2.0/html/al-be/.
Data format	Analyzed
Parameters for data collection	^10^Be ages from moraine boulders are used after detecting and removing outliers to maintain consistency. No bedrock sample data are used in this study. Limited statistical analyses were performed for moraines that have ≤2^10^Be ages before/after removing outliers. Former ELAs and change in ELAs (ΔELAs) are measured only for those glaciated valleys where the modern glacier-ice is present. Seventy-seven of the total 79 glaciated valleys fulfill the criteria.
Description of data collection	Approximately 500g of rock to a depth of ≤3.5cm from the top of each moraine boulder was collected using a hammer and chisel for^10^Be dating. Sample preparation was performed at the Quaternary Geochronology Laboratories in the University of Cincinnati. AMS measurements were performed at the Purdue Rare Isotope Measurement Laboratory at Purdue University. Raw data for ELA estimates were extracted using satellite images acquired in 26th February 2016 at https://search.earthdata.nasa.gov/search. Present and past glaciated areas were mapped (as vector layers) using Google Earth, and Landsat ETM + images and the raw elevation data were extracted from the ASTER GDEMs (as raster layers).
Data source location	New^10^Be samples were collected from Sonapani glacier in the Kulti valley (32.44° N, 77.33° E) and the Parkachik valley in Nun Kun massif (34.05° N, 76.00° E). Published^10^Be ages and all ELA dataset covers a vast area including the Himalaya, Tibet, Pamir, and Tian Shan with a latitudinal range of 27.04–43.12° N and longitudinal range of 71.62–102.74° E. Note that the sample coordinates are also provided in the tables.
Data accessibility	Data is provided in the paper.
Related research article	Saha, S., Owen, LA., Orr, E.N., Caffee, M.W. (2019). High-frequency Holocene glacier fluctuations in the Himalayan-Tibetan orogen. Quaternary Science Reviews, 220, 372–400.

**Table:** 

**Value of the data** • These data contain an exhaustive list of ^10^Be ages and reconstructed past ELAs and ΔELAs of moraines across the Himalaya, Tibet, Pamir, and Tian Shan for the past 15,000 years. • These data offer valuable information to anyone interested in the paleoclimatic changes in the region, especially in the past cryospheric (freshwater resources) responses to climate variability. • The ^10^Be data can be used/reproduced directly to recalculate exposure ages with future modifications in the dating techniques. • The ELA data are comprehensive and can be directly incorporated into numerical models that use terrestrial glaciers as a proxy for climate change. • The ^10^Be ages and ELAs may have the potentiality to model paleotemperatures in this high-altitude mountainous region.

## 1 Data

Table S1 contains all the new and published ^10^Be apparent moraine ages for the past 15 ka in the 79 glaciated regions of the Himalayan-Tibetan orogen (see also Supplementary item 1). We identified 128 outliers (in blue in Supplementary item 1) from a total age population of 535 in this study. Note that the ages are organized from oldest to youngest local glacial stages for each climatic zone. For an extended discussion on climatic zones, the readers are encouraged to see the article “High-frequency Holocene glacier fluctuations in the Himalayan-Tibetan orogen” in the Quaternary Science Reviews [1]. A comprehensive list of references is also provided in the article. Table 1 contains the ELAs and ΔELAs for the 77 glaciated regions (see also Supplementary item 2).

**Table 1:** Reconstructed ELAs and ΔELAs across the Himalayan-Tibetan orogen for the past ~15.

Glaciated Valley	Glacial Stage	Mean moraine age (ka)	Head (m asl)	Toe (m asl)	MELM (m asl)	Area-Altitude (m asl)	Area-Accumulation ratio			Toe-Headwall altitude ratio			Mean ELA (m asl)	ΔELA (m)
***Climatic Zone 1a: Arid and semiarid colder climatic region— northwest Tibet, Pamir, and Tian Shan.***					**MELM**	**AA**	**AAR (0.50)**	**AAR (0.60)**	**AAR (0.70)**	**THAR (0.30)**	**THAR (0.40)**	**THAR (0.50)**		
Bordoo Valley, Tian Shan	Present	-	4467	3870	4100	4188	4199	4159	4109	4037	4113	4190	4137±56	-
	BOR 1	0.64±0.23	4458	3745	-	4135	4159	4109	4039	3974	4059	4145	4089±67	54±10
	BOR 2	13.08±2.13	4452	3629	-	4044	4079	3979	3869	3890	3987	4085	3990±86	152±46
Kitschi-Kurumdu, Tian Shan	M2	15.16±3.03	4268	3787	-	3999	3969	3930	3899	-	-	-	3949±44	-
Ala Archa, Kyrgyz Tian Shan	Present	-	4299	3722	3950	4004	3989	3949	3889	3907	3973	4040	3963±50	-
	Ala Archa	0.49±0.25	4258	3400	-	3765	3739	3659	3569	3669	3769	3870	3720±97	244±50
Aksai valley, Kyrgyz Tian Shan	Present	-	4139	3843	4100	4006	3989	3979	3949	-	-	-	4005±57	-
	Aksai	5.70±0.16	4072	3408	-	3700	3689	3629	3549	3602	3683	3765	3660±71	339±46
Daxi valley, Tian Shan	Present	-	4400	3801	4000	4075	4049	4019	3969	3972	4043	4115	4030±50	-
	LIA	0.33±0.02	4390	3684	-	4013	3999	3949	3909	3909	3989	4070	3977±58	58±9
Alay Range (Koksu Valley)	Present	-	4917	3621	4059	4180	4209	4159	4099	4019	4149	4280	4144±84	-
	AV	14.02±0.16	4921	3442	-	4060	4129	4029	3899	3893	4041	4190	4034±110	122±39
Muztag Ata and Kongur Shan, NW Tibet	Present	-	6853	2773	3809	3972	3829	3729	3559	4003	4411	4820	4017±409	-
	Olimde 7 stage (m3I)	1.39±0.42	6873	2462	-	3870	3769	3619	3449	3792	4233	4675	3915±413	131±51
Muztag Ata and Kongur Shan, NW Tibet	Present	-	7349	4271	5609	5998	6259	5979	5609	5203	5511	5820	5749±332	-
	Olimde 7 stage (m7H)	1.66±0.17	7349	4054	-	5772	6029	5659	5229	5049	5379	5710	5547±341	222±100
Muztag Ata and Kongur Shan, NW Tibet	Present	-	6847	4300	5809	6030	6189	5989	5809	5068	5324	5580	5725±379	-
	Olimde 6 stage (m5H)	4.32±0.11	6852	4003	-	5866	6099	5879	5629	4864	5149	5435	5560±440	153±40
	Olimde 6 stage (m6H)	3.97±0.30	6852	3919	-	5793	6059	5829	5539	4801	5095	5390	5501±442	212±53
	Olimde 4 stage (m4H)	7.98±0.10	6839	3591	-	5437	5759	5279	4529	4574	4899	5225	5100±454	612±317
	Olimde 2 stage (m3H)	13.01±0.14	6852	3515	-	5377	5679	5069	4469	4521	4855	5190	5023±442	690±334
Muztag Ata and Kongur Shan, NW Tibet	Present	-	6991	4348	4969	5602	5459	5119	4849	5144	5409	5675	5278±301	-
	Olimde 8 stage (m6C)	0.51±0.15	7008	4273	-	5528	5389	4970	4769	5101	5375	5650	5255±317	68±42
	Olimde 2 stage (m5C)	11.71±0.40	7008	4209	-	5375	5089	4789	4599	5052	5333	5615	5122±352	201±126
Muztag Ata and Kongur Shan, NW Tibet	Present	-	6069	4237	4729	4936	4829	4749	4669	4791	4975	5160	4855±160	-
	Olimde 8 stage (m8A)	0.69±0.27	6075	4152	-	4860	4779	4689	4559	4735	4927	5120	4810±181	63±24
	Olimde 7 stage (m7A)	2.20±0.07	6075	4018	-	4803	4739	4619	4499	4637	4843	5050	4741±180	131±26
	Olimde 4 Stage (m6A)	7.80±0.29	6075	3982	-	4796	4729	4610	4489	4616	4825	5035	4729±179	144±28
	Olimde 4 Stage (m5A)	7.74±0.27	6075	3787	-	4621	4559	4409	4229	4476	4705	4935	4562±225	311±69
Muztag Ata and Kongur Shan, NW Tibet	Present	-	6667	4257	4969	5236	5369	5219	4909	4985	5227	5470	5173±201	-
	Olimde 3 stage (m3F)	10.25±0.16	6682	3586	-	4734	4609	4400	4169	4519	4829	5140	4629±313	574±196
Muztag Ata and Kongur Shan, NW Tibet	Present	-	6711	4601	4979	5316	5229	5109	4940	5242	5453	5665	5242±241	-
	Olimde 3 stage (m3F′)	9.69±0.34	6747	3410	-	4501	4489	4209	3929	4415	4750	5085	4483±370	797±140
Muztag Ata and Kongur Shan, NW Tibet	Present	-	6815	4258	4949	5488	5389	5129	4899	5027	5283	5540	5213±247	-
	Olimde 5 stage (m6C′)	5.05±0.14	6865	4059	-	5306	5150	4879	4669	4902	5183	5465	5079±276	172±72
Great Bogchigir Valley	Present	-	5324	4599	4919	4968	4979	4949	4899	4818	4891	4965	4924±54	-
	BO8 stage	13.18±0.64	5326	4425	-	4882	4919	4850	4760	4702	4793	4885	4827±78	97±26
***Climatic Zone 1a: Arid and semiarid colder climatic region— Transhimalaya***					**MELM**	**AA**	**AAR (0.45)**	**AAR (0.55)**	**AAR (0.65)**	**THAR (0.40)**	**THAR (0.50)**	**THAR (0.60)**		
Batura - Hunza Valley	Present	-	7606	2502	3489	4341	4359	3979	3719	4553	5065	5576	4385±689	-
	Batura stage (t6)	14.30±0.01	7617	2455	-	4276	4279	3919	3659	4527	5045	5562	4467±653	46±26
Batura - Hunza Valley	Present	-	6070	3226	4900	4966	5049	4859	4469	4481	4965	5448	4892±315	-
	Batura stage (t6)	12.49±1.05	7112	2542	-	4862	5009	4799	4249	4403	4900	5396	4803±381	88±61
Central Karakoram	Askole 2 stage (m2b)	5.98±0.69	-	-	-	-	-	-	-	-	-	-	-	-
Central Karakoram	Present	-	5173	4290	4829	4827	4859	4809	4779	4646	4735	4824	4789±69	-
	Mungo 2 stage (m2G)	6.64±0.35	5202	2991	-	4310	4729	4559	4089	3883	4105	4326	4286±291	497±232
	Mungo 2 stage (m1G)	13.77±0.53	5202	2889	-	3875	3639	3389	3289	3817	4050	4282	3763±353	1020±366
Central Karakoram	Present	-	6260	3027	4449	4792	4959	4839	4719	4325	4650	4974	4713±231	-
	Askole 3 stage (m1H)	1.03±0.28	6262	2977	-	4769	4949	4829	4689	4295	4625	4954	4730±228	21±8
	Mungo 2 stage (m3I)	13.06±0.40	6262	2977	-	4744	4939	4820	4669	4295	4625	4954	4721±226	30±13
	Mungo 2 stage (m2I)	14.08±0.23	6262	2977	-	4730	4939	4819	4649	4295	4625	4954	4716±226	35±21
	Mungo 2 stage (m1I)	14.98±0.29	6262	2977	-	4710	4929	4809	4619	4295	4625	4954	4706±225	45±32
Central Karakoram	Present	-	5840	4224	4939	5101	5159	5099	5039	4877	5040	5202	5057±108	-
	Mungo 2 stage (m1E)	12.41±0.33	5840	2643	-	4524	5029	4889	4509	3929	4250	4570	4529±370	545±293
Central Karakoram	Present	-	5718	4019	4689	4916	4979	4929	4839	4703	4875	5046	4872±125	-
	Mungo 2 stage (m1F)	13.44±0.19	5718	2873	-	4568	4859	4649	4409	4019	4305	4590	4486±271	413±186
Central Karakoram	Present	-	5700	3877	4509	4893	5050	4909	4709	4611	4795	4978	4807±186	-
		14.62±0.32	5724	2408	-	4338	4659	4429	4199	3737	4070	4402	4262±296	587±162
Ladakh cirque, Ladakh range	Present	-	5776	5407	5529	5596	5609	5570	5539	-	-	-	5529±110	-
	Ladakh Chang La cirque	2.29±0.28	5776	5376	-	5553	5549	5519	5480	-	-	-	5525±34	53±8
Ladakh cirque, Ladakh range	Present	-	5984	5386	5679	5673	5690	5659	5629	5629	5690	5750	5675±39	-
	Pangong high cirque	0.54±0.11	5997	4844	-	5517	5589	5509	5420	5313	5430	5546	5475±93	199±72
Stok Kangri, Zanskar	Present	-	5721	5288	5459	5507	5519	5499	5459	-	-	-	5489±28	-
	mS1	1.42±0.48	5748	5234	-	5456	5449	5419	5399	-	-	-	5431±27	65±13
Stok valley, Zanskar	mG1	-	5649	5258	5479	5510	5539	5509	5479	-	-	-	5503±25	-
	mG1	1.33±0.12	5649	5149	-	5455	5499	5459	5430	-	-	-	5461±29	49±6
Amda Kangri, Lato	Present	-	5743	5312	5489	5538	5539	5519	5489	-	-	-	5515±25	-
	mA1	0.26±0.08	5764	5298	-	5533	5539	5519	5489	-	-	-	5523±25	1±3
	mA2c	0.52±0.20	5764	5264	-	5511	5529	5489	5459	-	-	-	5497±30	24±10
Puga Valley, Zanskar	Present	-	6099	5686	5839	5893	5909	5870	5839	-	-	-	5870±32	-
	PM-3 stage	0.28±0.05	6101	5199	-	5647	5659	5609	5559	5563	5655	5746	5634±64	259±15
	PM-2 stage	3.50±0.87	6101	4797	-	5480	5569	5489	5369	5323	5455	5586	5467±96	401±55
Mentok Kangri, Karzok	Present	-	6003	5482	5659	5740	5759	5729	5709	-	-	-	5719±38	-
	mM1	0.64±0.09	6003	5447	-	5714	5739	5710	5679	-	-	-	5711±25	24±5
	mM2	1.00±0.08	6003	5378	-	5685	5689	5669	5639	5631	5695	5758	5681±42	64±8
Gomuche Kangri, Karzok	Present	-	6084	5381	5649	5873	5939	5909	5869	5669	5740	5810	5807±110	-
	mG1	2.25±0.42	6084	5332	-	5805	5909	5859	5739	5639	5715	5790	5779±91	50±39
	mG2 (or KM-4)	4.66±1.17	6084	5206	-	5732	5869	5739	5579	5561	5650	5738	5695±107	134±78
***Climatic Zone 1b: Arid and semiarid colder climatic region—southern and northeastern Tibet***					**MELM**	**AA**	**AAR (0.60)**	**AAR (0.70)**	**AAR (0.80)**	**THAR (0.30)**	**THAR (0.40)**	**THAR (0.50)**		
Dalijia Shan, NE Tibet	Group D moraines	13.45±0.25	4460	3725	4029	4079	4029	3949	3879	3951	4025	4100	4005±74	-
Xiying He valley, Qilian Shan, NE Tibet	Present	-	4729	4152	4379	4471	4439	4399	4349	-	-	-	4407±48	-
	N/A	13.16±1.05	4729	3397	-	3836	3689	3629	3579	3801	3935	4070	3791±174	731±65
NW Menyuan, Qilian Shan, NE Tibet	Gangshiga glacier (Present)	-	4812	4465	4579	4620	4599	4559	4519	-	-	-	4575±39	-
	Holocene moraine	10.08±0.53	4832	4320	-	4506	4459	4409	4379	-	-	-	4438±56	136±15
Anyemaqen Mountains	Halong glacial I (Present)	-	6183	4443	4939	5140	5339	4959	4729	4971	5145	5320	5068±207	-
	Halong glacial stage	9.48±1.70	6183	4428	-	4675	5109	4780	4639	4957	5133	5310	4943±255	143±167
Anyemaqen Mountains	Halong glacial II (Present)	-	6005	4549	4884	5347	5069	4989	4879	4987	5133	5280	5071±173	-
	Halong glacial stage	13.89±1.26	6006	3940	-	5268	4419	4309	4199	4564	4772	4980	4644±384	453±229
Kunlun Shan (northern slopes)	Present	-	5935	4593	5119	5861	5329	5239	5119	5001	5135	5270	5259±264	-
	M2 moraines	8.04±0.74	5960	4405	-	5693	5129	4999	4849	4877	5033	5190	5110±285	169±71
Karola Pass, Mt. Kaluxung, Sn Tibet	Present	-	6604	4860	5549	5861	5909	5789	5639	5385	5560	5735	5678±177	-
	Youngest	3.28±0.74			-	5754	5809	5629	5469	5369	5549	5730	5616±161	81±71
	Oldest	11.47±0.70	6623	4806	-	5200	5749	5529	5389	5355	5537	5720	5497±198	200±229
***Climatic Zone 2a: Transitional climatic region—western Himalaya***					**MELM**	**AA**	**AAR (0.45)**	**AAR (0.55)**	**AAR (0.65)**	**THAR (0.40)**	**THAR (0.50)**	**THAR (0.60)**		
Nun-Kun massif	Anantick stage (ST-3)	13.55±0.88			-	3685	3699	3639	3569	3705	3795	3884	3711±102	-
Nun-Kun massif	Present	-	5571	4238	4629	4905	5000	4830	4629	4775	4910	5044	4840±156	-
	Lonp stge (TG-3)	0.53±0.13	5575	3277	-	4605	4629	4509	4469	4199	4430	4660	4500±158	370±133
Yunam valley, Zanskar	Present	-	5601	5179	5369	5409	5410	5389	5369	-	-	-	5389±20	-
	Youngest	0.62±0.15	5640	4698	-	5231	5329	5269	5159	5079	5175	5270	5216±84	147±58
Lahul Himalaya, Nn India	Present	-	5378	3649	4639	4382	4389	4319	4219	4345	4520	4694	4438±164	-
	Kulti glacial stage	11.76±0.59	5378	2756	-	3854	3869	3689	3559	3811	4075	4338	3885±256	525±104
Lahul Himalaya, Nn India	Present	-	5746	3972	4639	4960	5049	4999	4939	4691	4870	5048	4899±157	-
	Kulti glacial stage	14.03±0.16	5746	2933	-	4530	4949	4659	4499	4067	4350	4632	4527±274	410±163
Lahul Himalaya, Nn India	Present	-	6002	4187	4909	5170	5269	5169	5060	4917	5100	5282	5109±142	-
	Kulti glacial stage	14.45±0.70	6002	3971	-	4822	4929	4650	4439	4791	4995	5198	4832±245	306±212
Lahul Himalaya, Nn India	Present	-	4225	3470	3749	3776	3739	3720	3709	3774	3850	3926	3780±73	-
	Kulti glacial stage	13.95±0.88	4782	2411	-	3404	3639	3419	3099	3367	3605	3842	3482±238	303±184
Hamtah Valley, Lahul	Present	-	5011	4056	4569	4459	4509	4449	4399	4443	4540	4636	4500±78	-
	mH1a	0.26±0.13	5063	3941	-	4407	4559	4409	4319	4397	4510	4622	4460±106	30±41
	mH3	10.48±0.48	5063	3688	-	4332	4419	4319	4180	4241	4380	4518	4341±112	149±47
Sonapani glacier, Kulti Valley, Lahul Himalaya	Present	-	5465	3901	4640	4815	4929	4819	4719	4533	4690	4846	4749±127	-
	mK1	-	5478	3601	-	4727	4879	4759	4590	4403	4585	4766	4673±157	92±31
	mK2	0.51±0.16	5478	3662	-	4704	4859	4739	4569	4397	4580	4762	4659±154	106±30
	mK3	-	5498	3631	-	4662	4839	4709	4529	4387	4575	4762	4638±153	127±38
	mK4	12.18±0.99	5474	3432	-	4641	4829	4699	4500	4255	4460	4664	4578±188	186±62
	mK5	15.30±0.60	5498	3151	-	4598	4809	4659	4469	4099	4335	4570	4506±232	259±109
Tons Valley, Garhwal Himalaya, Nn. India	Present	-	5883	3848	4679	4570	4449	4320	4259	4665	4870	5074	4611±274	-
	Location G	0.66±0.34	5883	3519	-	4433	4319	4249	4119	4467	4705	4942	4462±281	139±39
	Location F	6.09±0.54	5883	3277	-	4329	4269	4140	4049	4323	4585	4846	4363±271	238±59
	Location F'	10.26±0.35	5883	3211	-	4286	4250	4109	4020	4287	4555	4822	4333±273	268±63
Tons Valley, Garhwal Himalaya, Nn. India	Present	-	5924	4014	4680	5078	5199	5109	4989	4783	4975	5166	4997±183	-
	Location E	0.26±0.08	5955	3884	-	4997	5170	5039	4889	4717	4925	5132	4981±154	61±25
	Location D	11.09±0.50	5955	3527	-	4879	5109	4969	4719	4501	4745	4988	4844±204	198±69
	Location C	14.06±0.10	5955	3352	-	4166	4189	4059	3939	4065	4370	4674	4209±245	834±228
Gangotri, Garhwal Himalaya, Nn India	Present	-	6429	4581	4900	5084	5059	4969	4899	5093	-	-	5001±90	-
	Kedar glacial stage	8.28±0.45	6429	4218	-	5053	5030	4949	4869	5003	-	-	4981±74	40±28
Gangotri, Garhwal Himalaya, Nn India	Present	-	7003	4017	4900	5151	5149	5000	4879	5215	-	-	5049±143	-
	Bhujbas glacial stage	0.21±0.02	7004	3870	-	5132	5139	4990	4859	-	-	-	5050±124	15±6
	Gangotri glacial stage	0.56±0.30	7004	3874	-	5122	5129	4989	4859	-	-	-	5036±114	20±7
	Gangotri glacial stage	2.16±0.35	7010	3792	-	5101	5119	4979	4849	-	-	-	5023±111	33±12
	Shivling glacial stage	5.22±0.27	7010	2980	-	4998	5079	4939	4789	-	-	-	4900±178	94±41
Bhillanganga and Dudhanga valleys, Sn. Garhwal Himalaya	Present	-	6068	3806	4710	4956	5069	4919	4710	4717	4945	5172	4900±175	-
	mbd4	0.13±0.11	6068	3566	-	4918	5049	4889	4659	4573	4825	5058	4853±184	74±50
	mbd3	0.15±0.10	6082	3526	-	4871	5019	4849	4609	4534	4810	5066	4822±181	104±41
	mbd2	0.16±0.15	6082	3459	-	4840	4999	4819	4569	4511	4775	5038	4793±198	134±45
Bhillanganga and Dudhanga valleys, Sn. Garhwal Himalaya	Present	-	5616	4523	5019	5092	5079	5049	5019	4969	5080	5190	5062±66	-
	mbd1	0.21±0.02	5616	3776	-	5005	5060	5029	4999	4519	4705	4890	4887±202	182±188
Kedarnath, Sn. Garhwal Himalaya	Present	-	6136	3805	4730	4985	5189	4959	4730	4745	4980	5214	4941±195	-
	mk2	0.31±0.17	6136	3597	-	4897	5089	4830	4619	4619	4875	5130	4866±201	106±17
	mk1	10.25±0.83	6136	3180	-	4466	4519	4179	4039	4368	4665	4962	4457±306	515±206
Mayalil, Sn. Garhwal Himalaya	Present	-	5121	4620	4839	4893	4919	4869	4839	-	-	-	4872±35	-
	mm1	13.62±0.66	5121	4327	-	4758	4809	4759	4699	4649	4730	4810	4745±58	124±16
Nanda Devi, Garhwa, Nn Indial	Present	-	6862	3560	4879	5080	5279	4929	4639	4884	5215	5546	5056±285	-
	Moraine m4	0.60±0.28	6875	3478	-	4984	5169	4729	4549	4839	5180	5520	4996±325	86±60
	Moraine m2	13.71±0.69	6870	3432		4954	5119	4699	4529	4815	5160	5504	4971±356	113±66
Muguru valley, Gurla Mandhata	Present	-	6739	5621	5969	6190	6249	6159	6079	6077	6190	6302	6152±106	-
	M10	0.24±0.15	6739	5489	-	6115	6189	6089	5969	5981	6110	6238	6099±99	79±18
	M9	0.46±0.10	6753	5430	-	6062	6119	5999	5869	5962	6095	6228	6048±117	130±45
	M8	5.01±0.88	6760	5262	-	5935	5949	5829	5739	5869	6020	6170	5930±139	248±81
	M7	8.75±0.55	6760	5207	-	5904	5920	5800	5699	5767	5935	6102	5875±133	303±62
Muguru valley, Gurla Mandhata	Present	-	6108	5728	5960	5985	6019	5989	5959	-	-	-	5982±25	-
	M5	15.30±0.60	6108	5474	-	5756	5759	5659	5599	-	-	-	5693±78	295±61
***Climatic Zone 2b: Transitional climatic region—central and eastern Himalaya***					**MELM**	**AA**	**AAR (0.50)**	**AAR (0.60)**	**AAR (0.70)**	**THAR (0.30)**	**THAR (0.40)**	**THAR (0.50)**		
Rongbuk valley, N. Mt. Everest	Present	-	6847	5203	5609	5881	5859	5799	5719	5704	5869	6035	5809±132	-
	T7	0.33±0.19	6847	5162	-	5844	5839	5769	5669	5676	5845	6015	5808±119	30±11
	T6	2.08±0.09	6847	5128	-	5840	5839	5759	5659	5648	5821	5995	5794±120	44±13
	T5c	3.18±0.23	6847	5098	-	5832	5830	5759	5650	5627	5803	5980	5783±120	55±17
Khumbu Himal, Nepal	Present	-	7680	4855	5249	5521	5339	5280	5229	5708	5991	6275	5574±388	-
	Chhukung glacial stage	11.52±0.11	7699	4392	-	5477	5329	5259	5209	5392	5723	6055	5492±300	128±134
Khumbu Himal, Nepal	Present	-	5837	4760	5039	5025	4989	4959	4899	5087	5196	5305	5062±132	-
	Chhukung glacial stage	10.97±0.03	5870	4675	-	5004	4969	4929	4879	5039	5159	5280	5037±139	29±10
Khumbu Himal, Nepal	Present	-	6749	4926	5209	5381	5249	5179	5119	5478	5661	5845	5390±256	-
	Thuklha glacial stage	4.43±0.32	6800	4867	-	5361	5210	5140	5070	5451	5645	5840	5388±280	28±15
Hailuogou Valley, Gonga Shan, SE Tibet	Present	-	7331	2963	5080	5188	5219	5119	5039	4280	4714	5155	4974±321	-
	Little Ice Age moraines	0.46±0.06	7354	2911	-	5103	5190	5099	4999	4251	4695	5140	4925±339	34±24
	Neoglacial moraines	1.04±0.10	7354	2799	-	4931	5130	5040	4849	4167	4623	5080	4831±339	128±69
	Recessional moraine	6.03±1.97	7354	2482	-	4750	5099	4969	4149	3950	4437	4925	4611±440	348±262
	Local LGM moraines	10.10±0.73	7354	1697	-	4071	3769	3309	2969	3397	3963	4530	3715±529	1244±550
***Climatic Zone 3: Wet-temperate climatic region—central and eastern Himalaya***					**MELM**	**AA**	**AAR (0.50)**	**AAR (0.60)**	**AAR (0.70)**	**THAR (0.30)**	**THAR (0.40)**	**THAR (0.50)**		
Lete valley, Annapurna, Nepal		6.363±1.21	-	-	-	-	-	-	-	-	-	-	-	-
Ganhaizi and Ganheba, southeastern Tibet	Present	-	5108	4502	4670	4841	4839	4819	4779	4667	4743	4820	4772±72	-
	Ganhaizi	12.97±1.41	5108	2907	-	3998	3940	3559	3219	3554	3789	4025	3726±296	1061±273
	Ganheba	11.96±1.94	5174	2955	-	3715	3429	3359	3309	3608	3861	4115	3628±292	1159±301
Annapurna Range, Nepal	Syaktan glacier (Present)	-	5311	4987	5099	5144	5139	5109	5079	-	-	-	5114±27	-
	Syaktan glacier stage	9.48±0.91	5589	3722	-	4789	4899	4709	4539	4290	4477	4665	4624±205	384±124
Annapurna Range, Nepal	Lyapche glacier (Present)	-	6583	4764	5559	5734	5729	5639	5519	5315	5497	5680	5584±142	-
	Lyapche glacier stage	11.54±0.80	6641	3549	-	5380	5559	5439	5269	4479	4789	5100	5145±387	443±265
Annapurna Range, Nepal	Yak glacier (Present)	-	5144	4870	4979	5012	5009	4989	4969	-	-	-	4992±19	-
	Yak upper	8.72±0.40	5173	4440	-	4882	4929	4869	4789	4662	4736	4810	4811±92	128±41
	Yak lower	11.66±1.29	5179	4388	-	4821	4849	4789	4699	4629	4709	4790	4755±78	205±46
Annapurna Range, Nepal	Danfe Glacier (Present)	-	5420	4621	5029	5000	4899	4849	4809	4869	4949	5030	4929±85	-
	Danfe glacier stage	8.87±0.36	5446	4009	-	4457	4319	4269	4229	4444	4589	4735	4435±181	480±119
Milarepa's Glacier, Annapurna Range, Nepal	Present	-	5386	3879	4439	4551	4399	4359	4319	4334	4487	4640	4441±112	-
	N/A	0.55±0.16	5504	3513	-	4469	4379	4329	4239	4116	4315	4515	4337±135	104±73
Dudh Khola Valley, Annapurna, Nepal	Present	-	6673	3552	4529	5295	5679	4929	4529	4495	4807	5120	4923±423	-
	Neoglacial	1.70±0.50	6673	3084	-	5196	5559	4689	4449	4166	4525	4885	4781±474	198±98
Macha Khola Valley, Gorkha Himal, Nepal	Present	-	5268	4916	5049	5079	5079	5059	5039	-	-	-	5061±18	-
	MK4: LIA	4.99±0.92	5304	3735	-	4523	4659	4379	4079	4210	4367	4525	4392±199	654±230
Mailun Khola, Ganesh Himal, Nepal	Present	-	5530	4799	5089	5144	5149	5119	5069	5021	5095	5170	5107±48	-
	M3	7.04±0.64	5562	4257	-	5024	5090	5019	4939	4652	4783	4915	4917±153	192±119
Langtang Valley, Langtang Himal, Nepal	Present	-	6319	3983	4909	4861	4769	4629	4439	4691	4925	5160	4798±218	-
	Yala I	0.76±0.20	6328	3774	-	4648	4509	4339	4229	4547	4803	5060	4591±280	191±71
	LT6	4.42±0.15	6328	3506	-	4491	4329	4189	4049	4358	4641	4925	4426±292	356±77
Langtang Valley, Langtang Himal, Nepal	Present	-	6514	4392	5239	5369	5429	5309	5149	5035	5247	5460	5280±143	-
	Langtang Stage (LT3)	10.90±0.43	6525	3915	-	5169	5259	5069	4869	4702	4963	5225	5037±204	249±55
Langtang Valley, Langtang Himal, Nepal	Present	-	6563	4369	5139	5195	5069	5020	4949	5029	5249	5470	5140±166	-
	Langtang glacial stage II	4.60±0.33	6563	3344	-	4747	4869	4619	4449	4315	4637	4960	4657±226	484±163
	Langtang glacial stage I	5.47±0.40	6563	3079	-	4374	4499	4049	3419	4126	4475	4825	4252±448	888±315
Nyalam County, Sn Xixabangma, Sn Tibet	Fu Qu glacier (Present)	-	5566	4446	4929	4845	4709	4620	4579	4788	4901	5015	4798±153	-
	Puluo 1 moraine	!	5552	4013	-	4509	4439	4379	4329	4481	4635	4790	4509±158	271±39

- No data.

m asl Meter above sea level.

Note: The present here refers to the year 2016 AD.

## 2 Experimental design, materials, and methods

### 2.1 New ^10^Be ages

We sampled multiple (≥2) boulders from each moraine using a chisel and hammer after carefully considering the moraine morphostratigraphy, physical setting, and surficial characteristics of moraine boulders [2]. Moraines were first mapped and grouped from oldest to youngest based on their relative position from each other (i.e., morphostratigraphy). Since Holocene moraines show similar surficial characteristics, relative dating based on the degree of weathering, vegetation cover, and soil development was not possible. We recorded the stability, degradation, and post-depositional hillslope contribution on each moraine in the field before sampling. We only sampled well-inset stable boulders with no evidence of post-depositional surface deflections, detrital cover, surface spallation, pitting, fracturing, and/or extensive weathering. Preferences were also given to boulders with well-developed lichen cover with the idea that boulders have not recently been exhumed and/or toppled allowing the steady growth of lichens. The sampled boulders have heights ranging from 0.3 to 1.3 m (Table S1). Approximately 500 g of rock was collected from the top of each boulder to a depth was ≤3.5 cm. Topographic shielding from the boulder surface to the horizon was measured using a compass and an inclinometer at 10° azimuth interval [2], [3]. No correction for snow shielding was performed, assuming a windswept condition throughout the year [3].

Quartz extraction and ^10^Be sample preparation were executed at the Quaternary Geochronology Laboratories of the University of Cincinnati [4], [5]. Our sample preparation includes crushing and sieving the boulder samples to obtain a 250–500 μm particle size fraction. Subsequently, samples were leached for about 10 hours in aqua regia to remove any organics and dried for 24 hours. The dry samples were then etched in 1% HF for approximately 1 h. Since quartz is hydrophilic (sticky) in nature, the froth flotation technique was applied to remove excess muscovite and feldspar (which are hydrophobic) in the sample. Samples were then treated two to three times with 5% and 1% HF/HNO_3_. Any remaining feldspar, mica, and other heavy minerals were removed by using lithium heteropolytungstate heavy liquid separation (density 2.7 g/cm^3^) and a Frantz magnetic separator. About 25–15 g of extracted pure quartz was dissolved in 49% concentrated HF acid after adding low background ^9^Be carrier (0.495 mg/g for Kulti and 1.0459 mg/g for Parkachik) and fumed with perchloric (HClO_4_) acid to remove fluorine atoms. In addition, to remove Fe and Ti and separate the ^10^Be fraction, samples were passed through the anion and cation exchange columns using (6–1 N) HCl acid. Beryllium hydroxide (Be(OH)_2_) gel was extracted from the ^10^Be fraction by adding Ammonium hydroxide. The Be(OH)_2_ was heated in an oven at 900 °C for 30 minutes to form BeO, mixed with acetone, Nb powder, and then loaded into a steel target. A minimum of two blanks were prepared to assess the ^9^Be carrier and laboratory background level of ^10^Be for each set of samples. We measured the ratios of ^10^Be/^9^Be using the accelerator mass spectrometry (AMS) at the Purdue Rare Isotope Measurement (PRIME) Laboratory at Purdue University. 07KNSTD (standard) is used to normalize our Be isotopic. The ^10^Be/^9^Be ratios were subsequently converted into ^10^Be concentrations, i.e., in atoms [2] and exposure ages (Table S1) were estimated using the available online age calculators (https://crep.otelo.univ-lorraine.fr/#/; http://hess.ess.washington.edu/; http://cronus.cosmogenicnuclides.rocks/2.0/html/al-be/).

No corrections for residual boron, radioactive decay, and muongenic production [6] were made; they are negligible for the timescale of this study. Native ^9^Be in nearby (uniform) lithology is also insignificant (∼0.0190 ± 0.0160 to 0.0015 ± 0.0001 ppm in Ref. [1]) to account for any adjustments in our calculated exposure ages.

### 2.2 Published ^10^Be ages

For consistency, we followed a strict procedure while compiling the published ^10^Be ages. This includes only using moraine boulder ages, excluding any bedrock ages from the analysis (Table S1; Supplementary item 1). ^10^Be ages that do not follow the moraine morphostratigraphic order as outlined in the original literature were excluded. Slip rate studies on moraines that only dated pebbles/cobbles were also not used in this compilation. Only studies that used the standard [4], [5] ^10^Be extraction procedure are targeted. Since published studies used different standards (e.g., LLNL3000, S555, NIST_Certified, NIST_27900, KNSTD, 07KNSTD) to normalize their Be isotopic measurements (Table S1), a correction factor is used whenever required while recalculating the ages [7]. We used 5 cm as the maximum depth of sample collection and zero erosion rates for studies that did not report any such information. Using the raw data provided in the original literature, we therefore recalculated all the published ^10^Be ages following the same parameters (Table S1; Supplementary item 1).

### 2.3 Exposure age calculation

We calculated/recalculated ^10^Be ages using the community standard Cosmic Ray Exposure program (CREp of [12]), CRONUS-Earth V3 [3], and CRONUScale program [9] (Table S1). Apparent exposure ages are calculated using the scaling schemes of Lifton-Sato-Dunai (LSD; [8]), time-dependent Lal and Stone (Lm; [3]), and time-independent Lal and Stone (St; [10], [11]) (Table S1). The global sea-level high-latitude (SLHL) spallogenic ^10^Be production rate of 4.08 ± 0.23 atoms/g/a was used for the LSD scaling scheme along with the ERA40 atmospheric model and VDM2016 geomagnetic database [12], [13] (http://calibration.ice-d.org/). We assumed zero-erosion rates and reported all the ages in thousands of years (ka) before 2016 CE.

We performed several statistical treatments if > 2 concordant boulder ages are available for a moraine. We applied reduced chi-squared (χ^2^) statistics to assess the distribution of ages. Any age population with χ^2^ > 1 likely had outliers, and further statistical treatment was performed. Chauvenet's criterion [14] was used to detect outliers and highlighted in blue in Table S1. Outliers for new ^10^Be ages were only removed if convincing field evidence supported our statistical results (e.g., possible recent hillslope deposits, shallow burial, and/or toppling). For published studies, we relied on statistical treatment and the recommendations in the original studies to detect and remove outliers. Mean moraine ages (local glacial stages) are reported using arithmetic means ± 1σ, weighted mean ± 1σ, and peaks in the probability distribution (Table S1).

### 2.4 Equilibrium-line altitudes (ELAs)

Present and past ELAs were determined using area-altitude (AA), area accumulation ratio (AAR), and toe-headwall accumulation ratio (THAR) methods for each glacier advance in 77 glaciated valleys (Table 1; Supplementary item 2) [15]. Additionally, the modern maximum elevations of lateral moraines (MELM) were used to evaluate the modern estimated ELAs derived using the above three methods (Table 1). ELAs and ΔELAs are only measured for those glaciated valleys where the modern glacier-ice is present.

Raw data for ELA estimates were extracted using satellite images acquired in 26th February 2016 at https://search.earthdata.nasa.gov/search. Present and past glaciated areas were mapped as vector layers using Google Earth and Landsat ETM+ images in ArcGIS 10.5 (Supplementary item 2). In addition, we used ASTER GDEMs to prepare Hillshade and Slope maps (Spatial Analyst Tools in ArcGIS) to further aid in outlining modern glaciated areas and paleo-ice extents. Paleo-ice extents were defined on the satellite images using moraine positions in the individual valley (Supplementary item 2). The vector layers/maps of the modern and the past glacier extents are then used to extract the DEM values and converted into ASCII flies. The ASCII files were inserted into the Read ArcGrid program developed by Professor David Nash of the University of Cincinnati to generate the glacier's hypsometry. The Read ArcGrid program calculates basic statistics, including Elevation Relief Ratio (hypsometric integral), for a matrix of elevations. Using the steps outlined in Ref. [15] and a combination of AA, AAR, and THAR ratios, we finally measured the ELAs. Different combinations of AARs (e.g., ranging from 0.45 to 0.80) and THARs (e.g., varies from 0.3 to 0.6) were used depending on the glacier setting, physical characteristics, and climate (Table 1). We obtained these ratios from the published literature for each distinct climatic zone (see Ref. [1] for details on climatic zones and references therein). In Table 1, we report the (arithmetic) mean ELA and ΔELA with ± 1σ uncertainty.

## References

[1] Saha S., Owen L.A., Orr E.N., Caffee M.W. (2019). High-frequency Holocene glacier fluctuations in the Himalayan-Tibetan orogen. Quat. Sci. Rev..

[2] Gosse J.C., Phillips F.M. (2001). Terrestrial in situ cosmogenic nuclides: theory and application. Quat. Sci. Rev..

[3] Balco G., Stone J.O., Lifton N.A., Dunai T.J. (2008). A complete and easily accessible means of calculating surface exposure ages or erosion rates from ^10^Be and 26Al measurements. Quat. Geochronol..

[4] Kohl C.P., Nishiizumi K. (1992). Chemical isolation of quartz for measurement of in-situ-produced cosmogenic nuclides. Geochem. Cosmochim. Acta.

[5] Nishiizumi K., Finkel R.C., Caffee M.W., Southon J.R., Kohl C.P., Arnold J.R., Olinger C.T., Poths J., Klein J. (1994). Cosmogenic production of ^10^Be and ^26^Al on the surface of the Earth and underground. Eighth International Conference on Geochronology, Cosmochronology and Isotope Geochemistry.

[6] Braucher R., Brown E.T., Bourlès D.L., Colin F. (2003). In situ produced ^10^Be measurements at great depths: implications for production rates by fast muons. Earth Planet. Sci. Lett..

[7] Nishiizumi K., Imamura M., Caffee M.W., Southon J.R., Finkel R.C., Mcaninch J. (2007). Be AMS standards.

[8] Lifton N., Sato T., Dunai T.J. (2014). Scaling in situ cosmogenic nuclide production rates using analytical approximations to atmospheric cosmic-ray fluxes. Earth Planet. Sci. Lett..

[9] Marrero S.M., Phillips F.M., Borchers B., Lifton N., Aumer R., Balco G. (2016). Cosmogenic nuclide systematics and the CRONUScalc program. Quat. Geochronol..

[10] Lal D. (1991). Cosmic ray labeling of erosion surfaces: in situ nuclide production rates and erosion models. Earth Planet. Sci. Lett..

[11] Stone J.O. (2000). Air pressure and cosmogenic isotope production. J. Geophys. Res. C Ocean. Atmos..

[12] Martin L.C.P., Blard P.H., Balco G., Lavé J., Delunel R., Lifton N., Laurent V. (2017). The CREp program and the ICE-D production rate calibration database: a fully parameterizable and updated online tool to compute cosmic-ray exposure ages. Quat. Geochronol..

[13] Borchers B., Marrero S., Balco G., Caffee M., Goehring B., Lifton N., Nishiizumi K., Phillips F., Schaefer J., Stone J. (2016). Geological calibration of spallation production rates in the CRONUS-Earth project. Quat. Geochronol..

[14] Taylor J.R. (1997). An Introduction to Error Analysis.

[15] Osmaston H. (2005). Estimates of glacier equilibrium line altitudes by the area x altitude, the area x altitude balance ratio and the area x altitude balance index methods and their validation. Quat. Int..

