# Supplementary material for: Cosmogenic 10Be and equilibrium-line altitude dataset of Holocene glacier advances in the Himalayan-Tibetan orogen
Source: Data Brief. 2019 Aug 20;26:104412. doi: 10.1016/j.dib.2019.104412 (PMC6736771; doi:10.1016/j.dib.2019.104412)
Supplement: Multimedia component 3 [file mmc3.docx]

**Table 1.** ^10^Be surface-exposure ages (in thousands of years before AD 2016, labeled ‘ka’±1σ) from 79 glaciated valleys across the Himalayan-Tibetan orogen. Sample data, local glacial stages, exposure ages using different scaling-schemes, and associated age statistics are highlighted. A complete list of references is provided in the article “High-frequency Holocene glacier fluctuations in the Himalayan-Tibetan orogen” in the Quaternary Science Reviews.

| **Reference** | **Local Glacial Stage** | **Glacial stage or moraine name** | **Sample ID** | **Latitude (DD)** | **Longitude (DD)** | **Elevation (m a.s.l.)** | **Sample thickness (cm)** | **Density (g cm-3)** | **Erosion (cm a-1)** | **Shielding correction** | **[10Be] ± 1σ (104 atoms g -1)a** | **AMS std** | **Local glacial stage (based on LSD ages in ka)** | CREp based results | | | CRONUScale 2.0 based results | | | | | | | | CRONUS EARTH 3.0 based results | | | |
| --- | --- | --- | --- | --- | --- | --- | --- | --- | --- | --- | --- | --- | --- | --- | --- | --- | --- | --- | --- | --- | --- | --- | --- | --- | --- | --- | --- | --- |
|  |  |  |  |  |  |  |  |  |  |  |  |  |  | **LSD Age ± 1σ (ka)** | **Basic Statistics** | **Lm Age ± 1σ (ka)** | **SA Age ± 1σ (ka)** | **SF Age ± 1σ (ka)** | **Lm Age ± 1σ (ka)** | **St Age ± 1σ (ka)** | **De Age ± 1σ (ka)** | **Du Age ± 1σ (ka)** | **Li Age ± 1σ (ka)** | **LSD Age ± 1σ (ka)** | | **Lm Age ± 1σ (ka)** | **St Age ± 1σ (ka)** |  |
| ***Climatic Zone 1a: Arid and semiarid colder climatic region— Transhimalaya, northwestern Tibet, Pamir, and Tian Shan.*** | | | | | | | | | | | | | | | | | | | | | | | | | | | | |
| Zech (2012) | Kitschi-Kurumdu | M2 | KI11 | 40.7827 | 75.4876 | 3870 | 3 | 2.7 | 0 | 1 | 61.87±2.54 | 07KNSTD | 15.16±3.03 | 10.73±0.65 | Chi-squared: 17.74 | 11.56±0.67 | 11.44±0.99 | 12.00±1.00 | 11.00±1.00 | 12.00±1.00 | 11.90±1.80 | 12.20±1.70 | 11.60±1.60 | 11.22±0.46 | | 11.63±0.48 | 11.26±0.46 |  |
|  |  |  |  |  |  |  |  |  |  |  |  |  |  |  | Skewness: -1.62 |  |  |  |  |  |  |  |  |  |  |  |  |  |
| Zech (2012) | Kitschi-Kurumdu | M2 | KI12 | 40.7827 | 75.4876 | 3870 | 3 | 2.7 | 0 | 1 | 98.67±3.54 | 07KNSTD |  | 16.53±1.03 | Outlier: 1 | 18.01±0.99 | 17.50±1.30 | 17.80±1.50 | 18.30±1.60 | 18.20±1.50 | 18.30±2.60 | 18.40±2.50 | 17.90±2.30 | 16.41±0.59 | | 17.61±0.64 | 17.98±0.65 |  |
| Zech (2012) | Kitschi-Kurumdu | M2 | KI13 | 40.7826 | 75.4869 | 3870 | 3 | 2.7 | 0 | 1 | 105.42±3.22 | 07KNSTD |  | 17.63±1.04 | Wt. mean: 13.93±3.03 ka | 19.16±0.98 | 18.50±1.40 | 18.90±1.40 | 19.60±1.70 | 19.30±1.50 | 19.40±2.60 | 19.40±2.60 | 19.00±2.30 | 17.43±0.54 | | 18.74±0.58 | 19.22±0.59 |  |
| Zech (2012) | Kitschi-Kurumdu | M2 | KI14 | 40.7826 | 75.4869 | 3870 | 3 | 2.7 | 0 | 1 | 93.38±4.47 | 07KNSTD |  | 15.73±1.06 | Ar. Mean: 15.16±3.03 ka | 17.11±1.07 | 16.60±1.40 | 16.90±1.50 | 17.30±1.60 | 17.30±1.50 | 17.40±2.60 | 17.50±2.50 | 17.00±2.30 | 15.60±0.75 | | 16.73±0.80 | 17.01±0.82 |  |
| Zech (2012) | Kitschi-Kurumdu | M2 | KI21 | 40.7874 | 75.4728 | 3990 | 3 | 2.7 | 0 | 1 | 135.75±4.74 | 07KNSTD |  | 20.73±1.19 | Peak: ! | 22.71±1.20 | 21.60±1.60 | 22.00±1.80 | 23.70±2.10 | 22.90±1.90 | 22.80±3.30 | 22.60±3.20 | 22.10±2.80 | 20.52±0.72 | | 22.23±0.78 | 23.26±0.82 |  |
| Seong et al., 2007 | Mungo 2 stage | m1I | K2-72 | 35.703 | 75.953 | 4209 | 2 | 2.7 | 0 | 1 | 82.28±1.81 | KNSTD | 14.98±0.29 | 14.65±0.68 | Chi-squared: 0.17 | 14.4±0.74 | 15.30±1.10 | 15.50±1.20 | 14.60±1.10 | 14.50±1.20 | 15.00±2.00 | 15.00±2.00 | 14.80±1.80 | 13.86±0.31 | | 14.23±0.31 | 14.23±0.31 |  |
| Seong et al., 2007 | Mungo 2 stage | m1I | K2-73 | 35.704 | 75.952 | 4213 | 2 | 2.7 | 0 | 1 | 84.09±1.81 | KNSTD |  | 14.91±0.69 | Skewness: +0.97 | 14.66±0.75 | 15.50±1.10 | 15.80±1.20 | 14.80±1.10 | 14.80±1.30 | 15.30±2.10 | 15.60±2.10 | 15.10±1.80 | 14.06±0.30 | | 14.50±0.31 | 14.52±0.31 |  |
| Seong et al., 2007 | Mungo 2 stage | m1I | K2-74 | 35.703 | 75.952 | 4212 | 2 | 2.7 | 0 | 1 | 84.99±1.81 | KNSTD |  | 15.04±0.7 | Outlier: 1 | 14.81±0.76 | 15.70±1.10 | 16.00±1.30 | 15.00±1.10 | 14.90±1.30 | 15.50±2.10 | 15.80±2.10 | 15.20±1.80 | 14.21±0.30 | | 14.66±0.31 | 14.68±0.31 |  |
| Seong et al., 2007 | Mungo 2 stage | m1I | K2-75 | 35.703 | 75.948 | 4215 | 3 | 2.7 | 0 | 1 | 83.19±2.71 | KNSTD |  | 14.87±0.76 | Wt. mean: 14.98±0.29 ka | 14.62±0.81 | 15.50±1.10 | 15.70±1.30 | 14.80±1.20 | 14.70±1.30 | 15.30±2.10 | 15.60±2.10 | 15.00±1.90 | 14.01±0.46 | | 14.45±0.47 | 14.47±0.47 |  |
| Seong et al., 2007 | Mungo 2 stage | m1I | K2-76 | 35.703 | 75.947 | 4211 | 3 | 2.7 | 0 | 1 | 107.60±2.71 | KNSTD |  | 18.89±0.91 | Ar. Mean: 14.98±0.29 ka | 18.41±1 | 19.30±1.20 | 19.60±1.30 | 18.90±1.40 | 19.10±1.60 | 19.20±2.30 | 19.40±2.30 | 18.90±2.20 | 17.70±0.45 | | 18.36±0.47 | 18.77±0.48 |  |
| Seong et al., 2007 | Mungo 2 stage | m1I | K2-77 | 35.703 | 75.943 | 4211 | 3 | 2.7 | 0 | 1 | 86.80±1.81 | KNSTD |  | 15.44±0.72 | Peak: 14.95 ka | 15.15±0.79 | 16.10±1.20 | 16.40±1.30 | 15.40±1.20 | 15.40±1.30 | 15.90±2.20 | 16.20±2.20 | 16.00±2.00 | 14.62±0.31 | | 15.04±0.31 | 15.13±0.32 |  |
| Seong et al., 2007 | Mungo 2 stage | m1D (bedrock) | K2-23 | 35.718 | 75.52 | 2646 | 3 | 2.7 | 0 | 0.96 | 33.46±0.90 | KNSTD | 14.62±0.32 | 14.96±0.74 | Chi-squared: 0.19 | 15.51±0.85 | 16.50±1.20 | 16.50±1.30 | 14.90±1.20 | 14.80±1.30 | 16.80±2.30 | 17.00±2.30 | 16.60±2.20 | 14.96±0.41 | | 14.52±0.39 | 14.55±0.40 |  |
|  |  |  |  |  |  |  |  |  |  |  |  |  |  |  | Skewness: +0.70 |  |  |  |  |  |  |  |  |  |  |  |  |  |
|  |  |  |  |  |  |  |  |  |  |  |  |  |  |  | Outlier: 1 |  |  |  |  |  |  |  |  |  |  |  |  |  |
| Seong et al., 2007 | Mungo 2 stage | m1D (bedrock) | K2-24 | 35.718 | 75.52 | 2631 | 3 | 2.7 | 0 | 0.96 | 31.65±0.90 | KNSTD |  | 14.33±0.71 | Wt. mean: 14.61±0.32 ka | 14.92±0.83 | 15.80±1.20 | 15.90±1.30 | 14.30±1.10 | 14.20±1.20 | 16.10±2.30 | 16.30±2.30 | 16.00±2.10 | 14.37±0.41 | | 13.91±0.40 | 13.89±0.40 |  |
| Seong et al., 2007 | Mungo 2 stage | m1D (bedrock) | K2-25 | 35.718 | 75.52 | 2631 | 3 | 2.7 | 0 | 0.96 | 30.74±0.90 | KNSTD |  | 13.96±0.72 | Ar. Mean: 14.62±0.32 ka | 14.55±0.8 | 15.40±1.10 | 15.50±1.20 | 13.90±1.10 | 13.80±1.20 | 15.70±2.20 | 15.90±2.20 | 16.00±2.00 | 13.98±0.41 | | 13.60±0.40 | 13.49±0.40 |  |
| Seong et al., 2007 | Mungo 2 stage | m1D (bedrock) | K2-26 | 35.719 | 75.52 | 2650 | 3 | 2.7 | 0 | 0.96 | 32.55±0.90 | KNSTD |  | 14.57±0.71 | Peak: 14.62 ka | 15.09±0.84 | 16.00±1.20 | 16.10±1.30 | 14.50±1.20 | 14.40±1.30 | 16.40±2.30 | 16.60±2.30 | 16.20±2.10 | 14.58±0.41 | | 14.11±0.39 | 14.12±0.39 |  |
| Owen et al., 2002 | Batura stage | t6 | KK98-34a | 36.51 | 74.89 | 2600 | 5 | 2.7 | 0 | 0.99 | 28.09±0.86 | LLNL3000 | 14.30±0.01 | 12.69±0.68 | Chi-squared: ! | 13.14±0.76 | 14.00±1.10 | 14.00±1.20 | 12.50±1.10 | 12.40±1.10 | 14.30±2.00 | 15.00±2.00 | 14.10±1.80 | 12.88±0.40 | | 12.49±0.39 | 12.16±0.38 |  |
|  |  |  |  |  |  |  |  |  |  |  |  |  |  |  | Skewness: ! |  |  |  |  |  |  |  |  |  |  |  |  |  |
|  |  |  |  |  |  |  |  |  |  |  |  |  |  |  | Outlier: 1 |  |  |  |  |  |  |  |  |  |  |  |  |  |
|  |  |  |  |  |  |  |  |  |  |  |  |  |  |  | Wt. mean: 14.30±0.01 ka |  |  |  |  |  |  |  |  |  |  |  |  |  |
| Owen et al., 2002 | Batura stage | t6 | KK98-34b | 36.51 | 74.89 | 2610 | 5 | 2.7 | 0 | 0.99 | 32.24±0.86 | LLNL3000 |  | 14.31±0.7 | Ar. Mean: 14.30±0.01 ka | 14.81±0.81 | 15.60±1.20 | 15.70±1.30 | 14.30±1.10 | 14.20±1.20 | 16.10±2.30 | 16.30±2.30 | 15.90±2.10 | 14.28±0.38 | | 13.90±0.37 | 13.88±0.37 |  |
| Owen et al., 2002 | Batura stage | t6 | KK98-35 | 36.51 | 74.89 | 2595 | 5 | 2.7 | 0 | 0.99 | 31.90±0.86 | LLNL3000 |  | 14.29±0.71 | Peak: 14.30 ka | 14.8±0.81 | 15.60±1.20 | 15.70±1.20 | 14.20±1.10 | 14.10±1.20 | 16.10±2.30 | 16.30±2.30 | 15.90±2.10 | 14.28±0.39 | | 13.88±0.38 | 13.86±0.38 |  |
| Seong et al., 2007 | Mungo 2 stage | m1I | K2-78 | 35.695 | 75.957 | 4225 | 2 | 2.7 | 0 | 1 | 84.99±1.81 | KNSTD | 14.08±0.23 | 14.96±0.69 | Chi-squared: 0.11 | 14.73±0.76 | 15.60±1.10 | 15.90±1.20 | 14.90±1.10 | 14.80±1.30 | 15.40±2.10 | 15.70±2.10 | 15.10±1.80 | 14.12±0.30 | | 14.57±0.31 | 14.59±0.31 |  |
| Seong et al., 2007 | Mungo 2 stage | m1I | K2-79 | 35.694 | 75.958 | 4221 | 2 | 2.7 | 0 | 1 | 80.47±1.81 | KNSTD |  | 14.26±0.67 | Skewness: -1.29 | 14.02±0.76 | 15.00±1.00 | 15.20±1.10 | 14.20±1.10 | 14.10±1.20 | 15.00±1.90 | 15.00±1.90 | 14.50±1.80 | 13.59±0.31 | | 13.89±0.31 | 13.84±0.31 |  |
| Seong et al., 2007 | Mungo 2 stage | m2I | K2-80 | 35.694 | 75.958 | 3823 | 3 | 2.7 | 0 | 0.81 | 64.20±1.81 | KNSTD |  | 17.14±0.87 | Outlier: 5 | 16.99±0.96 | 18.00±1.30 | 18.20±1.40 | 17.10±1.40 | 17.20±1.50 | 18.00±2.40 | 18.20±2.40 | 17.70±2.20 | 16.31±0.46 | | 16.61±0.47 | 16.86±0.48 |  |
| Seong et al., 2007 | Mungo 2 stage | m2I | K2-81 | 35.693 | 75.96 | 3821 | 3 | 2.7 | 0 | 0.81 | 66.01±1.81 | KNSTD |  | 17.6±0.89 | Wt. mean: 14.08±0.23 ka | 17.43±0.98 | 18.40±1.30 | 18.70±1.40 | 17.60±1.40 | 17.70±1.50 | 18.40±2.40 | 18.60±2.40 | 18.10±2.30 | 16.75±0.46 | | 17.07±0.47 | 17.35±0.48 |  |
| Seong et al., 2007 | Mungo 2 stage | m2I | K2-82 | 35.692 | 75.96 | 3818 | 2 | 2.7 | 0 | 0.81 | 63.29±1.81 | KNSTD |  | 16.83±0.86 | Ar. Mean: 14.08±0.23 ka | 16.66±0.96 | 17.70±1.30 | 17.90±1.40 | 16.80±1.40 | 16.80±1.50 | 17.70±2.40 | 17.90±2.40 | 17.40±2.20 | 16.02±0.46 | | 16.31±0.47 | 16.53±0.47 |  |
| Seong et al., 2007 | Mungo 2 stage | m2I | K2-83 | 35.692 | 75.961 | 3779 | 3 | 2.7 | 0 | 1 | 231.48±5.43 | KNSTD |  | 48.51±3.17 | Peak: 14.09 ka | 45.91±3.44 | 44.50±3.30 | 45.30±3.70 | 46.00±3.90 | 51.80±4.50 | 46.90±6.80 | 45.60±6.80 | 45.50±6.20 | 44.03±1.04 | | 46.56±1.10 | 50.89±1.21 |  |
| Seong et al., 2007 | Mungo 2 stage | m2I | K2-84 | 35.689 | 75.941 | 3768 | 2 | 2.7 | 0 | 1 | 61.49±1.81 | KNSTD |  | 13.82±0.71 |  | 13.82±0.78 | 14.70±1.10 | 14.90±1.10 | 13.70±1.10 | 13.60±1.20 | 15.00±1.90 | 15.00±1.90 | 14.40±1.80 | 13.40±0.40 | | 13.50±0.40 | 13.36±0.39 |  |
| Seong et al., 2007 | Mungo 2 stage | m2I | K2-85 | 35.689 | 75.941 | 3766 | 2 | 2.7 | 0 | 0.99 | 62.39±2.71 | KNSTD |  | 14.15±0.82 |  | 14.13±0.88 | 15.00±1.10 | 15.20±1.20 | 14.10±1.20 | 14.00±1.30 | 14.90±2.00 | 15.00±2.00 | 14.70±1.90 | 13.68±0.60 | | 13.79±0.60 | 13.71±0.60 |  |
| Abramowski et al., 2006 | N/A | AV | AV1 | 39.68 | 71.62 | 3440 | 4 | 2.7 | 0 | 1 | 56.57±3.65 | S555 | 14.02±0.16 | 13.82±1.02 | Chi-squared: 0.04 | 13.24±1.05 | 14.10±1.30 | 14.30±1.40 | 13.80±1.40 | 13.70±1.50 | 14.60±2.10 | 14.80±2.10 | 14.00±2.00 | 13.17±0.85 | | 13.53±0.88 | 13.48±0.87 |  |
|  |  |  |  |  |  |  |  |  |  |  |  |  |  |  | Skewness: +0.07 |  |  |  |  |  |  |  |  |  |  |  |  |  |
|  |  |  |  |  |  |  |  |  |  |  |  |  |  |  | Outlier: 0 |  |  |  |  |  |  |  |  |  |  |  |  |  |
|  |  |  |  |  |  |  |  |  |  |  |  |  |  |  | Wt. mean: 14.02±0.21 ka |  |  |  |  |  |  |  |  |  |  |  |  |  |
| Abramowski et al., 2006 | N/A | AV | AV2 | 39.68 | 71.62 | 3440 | 4 | 2.7 | 0 | 1 | 57.48±3.65 | S555 |  | 14.02±1.01 | Ar. Mean: 14.02±0.21 ka | 13.45±1.04 | 14.30±1.30 | 14.50±1.40 | 14.10±1.40 | 13.90±1.50 | 14.90±2.20 | 15.00±2.20 | 15.00±2.00 | 13.35±0.85 | | 13.71±0.87 | 13.69±0.87 |  |
| Abramowski et al., 2006 | N/A | AV | AV3 | 39.68 | 71.62 | 3440 | 4 | 2.7 | 0 | 1 | 58.39±3.65 | S555 |  | 14.23±1.02 | Peak: 14.02 ka | 13.65±1.03 | 14.50±1.30 | 14.70±1.40 | 14.30±1.40 | 14.20±1.50 | 15.10±2.20 | 15.20±2.20 | 15.00±2.00 | 13.53±0.85 | | 13.89±0.87 | 13.91±0.87 |  |
| Seong et al., 2007 | Mungo 2 stage | m1G | K2-48 | 35.671 | 75.799 | 3633 | 2 | 2.7 | 0 | 1 | 54.25±1.81 | KNSTD | 13.77±0.53 | 13.17±0.69 | Chi-squared: 0.56 | 13.26±0.77 | 14.10±1.10 | 14.30±1.20 | 13.10±1.10 | 12.90±1.20 | 14.00±1.90 | 14.40±1.90 | 13.90±1.80 | 12.94±0.43 | | 12.94±0.43 | 12.68±0.42 |  |
| Seong et al., 2007 | Mungo 2 stage | m1G | K2-49 | 35.671 | 75.799 | 3631 | 2 | 2.7 | 0 | 1 | 55.16±1.81 | KNSTD |  | 13.39±0.69 | Skewness: -0.20 | 13.48±0.76 | 14.30±1.10 | 14.50±1.10 | 13.30±1.10 | 13.10±1.20 | 14.00±1.90 | 14.60±1.90 | 14.10±1.80 | 13.09±0.43 | | 13.10±0.43 | 12.90±0.42 |  |
| Seong et al., 2007 | Mungo 2 stage | m1G | K2-50 | 35.671 | 75.799 | 3640 | 2 | 2.7 | 0 | 1 | 58.77±1.81 | KNSTD |  | 14.12±0.73 | Outlier: 1 | 14.17±0.8 | 15.10±1.10 | 15.20±1.20 | 14.10±1.10 | 13.90±1.20 | 15.00±2.00 | 15.00±2.00 | 14.80±1.80 | 13.71±0.42 | | 13.77±0.43 | 13.69±0.42 |  |
| Seong et al., 2007 | Mungo 2 stage | m1G | K2-51 | 35.671 | 75.799 | 3644 | 2 | 2.7 | 0 | 1 | 54.25±1.81 | KNSTD |  | 13.1±0.7 | Wt. mean: 13.76±0.53 ka | 13.18±0.77 | 14.00±1.10 | 14.20±1.10 | 13.00±1.10 | 12.80±1.10 | 14.00±1.90 | 14.40±1.90 | 13.80±1.80 | 12.89±0.43 | | 12.88±0.43 | 12.60±0.42 |  |
| Seong et al., 2007 | Mungo 2 stage | m1G | K2-52 | 35.667 | 75.798 | 3636 | 2 | 2.7 | 0 | 1 | 54.25±1.81 | KNSTD |  | 13.15±0.7 | Ar. Mean: 13.77±0.53 ka | 13.24±0.77 | 14.10±1.10 | 14.30±1.20 | 13.00±1.10 | 12.90±1.20 | 14.00±1.90 | 14.40±1.90 | 13.90±1.80 | 12.93±0.43 | | 12.92±0.43 | 12.66±0.42 |  |
| Seong et al., 2007 | Mungo 2 stage | m1G | K2-53 | 35.667 | 75.797 | 3643 | 4 | 2.7 | 0 | 1 | 58.77±1.81 | KNSTD |  | 14.33±0.72 | Peak: 13.80 ka | 14.38±0.79 | 15.20±1.10 | 15.40±1.20 | 14.30±1.20 | 14.10±1.20 | 15.20±2.10 | 15.60±2.10 | 15.00±1.90 | 13.85±0.43 | | 13.92±0.43 | 13.90±0.43 |  |
| Seong et al., 2007 | Mungo 2 stage | m1G | K2-54 | 35.666 | 75.797 | 3824 | 3 | 2.7 | 0 | 1 | 43.40±0.90 | KNSTD |  | 9.86±0.47 |  | 9.96±0.54 | 10.40±0.86 | 10.56±0.93 | 9.47±0.84 | 9.39±0.79 | 10.30±1.60 | 10.80±1.60 | 10.10±1.40 | 10.16±0.21 | | 10.02±0.21 | 9.23±0.19 |  |
| Seong et al., 2007 | Mungo 2 stage | m1G | K2-55 | 35.666 | 75.796 | 3825 | 5 | 2.7 | 0 | 1 | 64.20±1.81 | KNSTD |  | 14.32±0.7 |  | 14.28±0.77 | 15.10±1.10 | 15.40±1.20 | 14.20±1.10 | 14.10±1.20 | 15.10±2.00 | 15.00±2.00 | 14.90±1.80 | 13.78±0.39 | | 13.92±0.39 | 13.89±0.39 |  |
| Seong et al., 2007 | Mungo 2 stage | m1G | K2-56 | 35.668 | 75.797 | 3881 | 2 | 2.7 | 0 | 1 | 66.01±1.81 | KNSTD |  | 13.96±0.7 |  | 13.9±0.78 | 15.00±1.00 | 15.00±1.10 | 13.90±1.10 | 13.80±1.20 | 15.00±1.90 | 15.00±1.90 | 14.50±1.80 | 13.48±0.37 | | 13.64±0.38 | 13.52±0.37 |  |
| Seong et al., 2007 | Mungo 2 stage | m1G | K2-58 | 35.694 | 75.719 | 3879 | 2 | 2.7 | 0 | 1 | 65.10±2.71 | KNSTD |  | 13.8±0.79 |  | 13.75±0.84 | 14.60±1.10 | 14.80±1.20 | 13.70±1.20 | 13.60±1.30 | 14.50±2.00 | 15.00±2.00 | 14.30±1.90 | 13.33±0.56 | | 13.48±0.56 | 13.34±0.56 |  |
| Seong et al., 2007 | Mungo 2 stage | m1G | K2-59 | 35.694 | 75.719 | 3811 | 3 | 2.7 | 0 | 1 | 65.10±1.81 | KNSTD |  | 14.38±0.7 |  | 14.32±0.77 | 15.20±1.10 | 15.40±1.20 | 14.30±1.10 | 14.20±1.20 | 15.10±2.00 | 15.00±2.00 | 14.90±1.80 | 13.82±0.39 | | 13.96±0.39 | 13.94±0.39 |  |
| Seong et al., 2007 | Mungo 2 stage | m1F (bedrock) | K2-60 | 35.694 | 75.719 | 3031 | 2 | 2.7 | 0 | 0.94 | 37.07±0.90 | KNSTD | 13.44±0.19 | 13.43±0.64 | Chi-squared: 0.08 | 13.81±0.75 | 15.00±1.00 | 14.80±1.10 | 13.30±1.10 | 13.20±1.10 | 14.80±2.00 | 15.00±2.00 | 14.60±1.80 | 13.38±0.33 | | 13.12±0.32 | 12.94±0.32 |  |
|  |  |  |  |  |  |  |  |  |  |  |  |  |  |  | Skewness: +0.16 |  |  |  |  |  |  |  |  |  |  |  |  |  |
|  |  |  |  |  |  |  |  |  |  |  |  |  |  |  | Outlier: 1 |  |  |  |  |  |  |  |  |  |  |  |  |  |
| Seong et al., 2007 | Mungo 2 stage | m1F (bedrock) | K2-61 | 35.694 | 75.718 | 3032 | 2 | 2.7 | 0 | 0.93 | 36.17±0.90 | KNSTD |  | 13.25±0.64 | Wt. mean: 13.42±0.19 ka | 13.63±0.74 | 14.00±1.00 | 14.60±1.10 | 13.10±1.10 | 13.00±1.10 | 15.00±1.90 | 14.90±1.90 | 14.50±1.80 | 13.21±0.33 | | 12.98±0.33 | 12.75±0.32 |  |
| Seong et al., 2007 | Mungo 2 stage | m1F (bedrock) | K2-62 | 35.672 | 75.814 | 3017 | 2 | 2.7 | 0 | 0.94 | 34.36±1.81 | KNSTD |  | 12.63±0.86 | Ar. Mean: 13.44±0.19 ka | 13.02±0.93 | 13.80±1.20 | 13.90±1.30 | 12.50±1.20 | 12.30±1.20 | 14.00±2.00 | 14.00±2.00 | 13.80±1.90 | 12.75±0.67 | | 12.46±0.66 | 12.10±0.64 |  |
| Seong et al., 2007 | Mungo 2 stage | m1F (bedrock) | K2-63 | 35.672 | 75.814 | 2944 | 2 | 2.7 | 0 | 0.69 | 26.22±0.90 | KNSTD |  | 13.63±0.72 | Peak: 13.43 ka | 14.03±0.83 | 14.90±1.10 | 15.00±1.20 | 13.50±1.20 | 13.40±1.20 | 15.10±2.10 | 15.40±2.10 | 14.90±1.90 | 13.57±0.47 | | 13.26±0.46 | 13.10±0.45 |  |
| Röhringer et al., 2012 | BO8 stage | N/A | BH12 | 37.649 | 72.78 | 4451 | 3 | 2.7 | 0 | 0.96 | 108.28±7.07 | S555 | 13.18±0.64 | 16.81±1.26 | Chi-squared: 0.65 | 15.79±1.21 | 16.80±1.60 | 17.10±1.60 | 16.80±1.70 | 16.90±1.80 | 16.80±2.50 | 17.00±2.50 | 16.40±2.30 | 15.30±1.00 | | 16.32±1.07 | 16.58±1.09 |  |
|  |  |  |  |  |  |  |  |  |  |  |  |  |  |  | Skewness: ! |  |  |  |  |  |  |  |  |  |  |  |  |  |
|  |  |  |  |  |  |  |  |  |  |  |  |  |  |  | Outlier: 1 |  |  |  |  |  |  |  |  |  |  |  |  |  |
|  |  |  |  |  |  |  |  |  |  |  |  |  |  |  | Wt. mean: 13.21±0.64 ka |  |  |  |  |  |  |  |  |  |  |  |  |  |
| Röhringer et al., 2012 | BO8 stage | N/A | BH13 | 37.649 | 72.78 | 4451 | 2 | 2.7 | 0 | 0.96 | 87.05±3.45 | S555 |  | 13.63±0.76 | Ar. Mean: 13.18±0.64 ka | 12.96±0.83 | 13.80±1.10 | 14.10±1.20 | 13.60±1.20 | 13.40±1.20 | 14.00±1.90 | 14.00±1.90 | 13.40±1.80 | 12.79±0.51 | | 13.31±0.53 | 13.21±0.53 |  |
| Röhringer et al., 2012 | BO8 stage | N/A | BH14 | 37.649 | 72.78 | 4449 | 4 | 2.7 | 0 | 0.96 | 79.38±3.73 | S555 |  | 12.73±0.82 | Peak: 13.20 ka | 12.02±0.84 | 12.90±1.10 | 13.10±1.20 | 12.60±1.20 | 12.50±1.20 | 13.00±1.90 | 13.10±1.90 | 12.50±1.70 | 12.11±0.57 | | 12.56±0.59 | 12.25±0.58 |  |
| Blomdin et al. (2016) | BOR 2 | N/A | TS-C-12-023 | 41.8158 | 78.1151 | 3734 | 4 | 2.7 | 0 | 0.99 | 74.79±2.59 | 07KNSTD | 13.08±2.13 | 13.53±0.82 | Chi-squared: 10.59 | 14.75±0.79 | 14.30±1.10 | 14.60±1.20 | 14.80±1.30 | 14.90±1.20 | 15.10±2.20 | 15.20±2.10 | 14.70±1.90 | 13.63±0.47 | | 14.49±0.50 | 14.57±0.51 |  |
|  |  |  |  |  |  |  |  |  |  |  |  |  |  |  | Skewness: -0.93 |  |  |  |  |  |  |  |  |  |  |  |  |  |
|  |  |  |  |  |  |  |  |  |  |  |  |  |  |  | Outlier: 0 |  |  |  |  |  |  |  |  |  |  |  |  |  |
|  |  |  |  |  |  |  |  |  |  |  |  |  |  |  | Wt. mean: 12.56±2.13 ka |  |  |  |  |  |  |  |  |  |  |  |  |  |
| Blomdin et al. (2016) | BOR 2 | N/A | TS-C-12-025 | 41.8144 | 78.1118 | 3706 | 3 | 2.7 | 0 | 0.99 | 58.05±1.54 | 07KNSTD |  | 10.73±0.59 | Ar. Mean: 13.08±2.13 ka | 11.64±0.59 | 11.40±0.95 | 11.62±0.95 | 11.58±0.99 | 11.71±0.96 | 12.00±1.80 | 12.30±1.70 | 11.70±1.60 | 11.24±0.30 | | 11.72±0.31 | 11.38±0.30 |  |
| Blomdin et al. (2016) | BOR 2 | N/A | TS-C-12-026 | 41.8143 | 78.1118 | 3706 | 1 | 2.7 | 0 | 1 | 84.79±1.37 | 07KNSTD |  | 14.93±0.80 | Peak: ! | 16.29±0.77 | 15.70±1.10 | 16.00±1.30 | 16.50±1.40 | 16.50±1.30 | 16.70±2.40 | 16.80±2.30 | 16.30±2.10 | 14.95±0.24 | | 15.98±0.26 | 16.20±0.26 |  |
| Seong et al., 2007 | Mungo 2 stage | m3I | K2-86 | 35.689 | 75.941 | 3445 | 3 | 2.7 | 0 | 0.98 | 46.11±0.90 | KNSTD | 13.06±0.40 | 12.82±0.62 | Chi-squared: 0.33 | 13±0.72 | 13.81±0.99 | 14.00±1.10 | 13.00±1.00 | 12.50±1.10 | 14.00±1.90 | 14.20±1.90 | 13.60±1.80 | 12.74±0.25 | | 12.63±0.25 | 12.29±0.24 |  |
|  |  |  |  |  |  |  |  |  |  |  |  |  |  |  | Skewness: +1.72 |  |  |  |  |  |  |  |  |  |  |  |  |  |
|  |  |  |  |  |  |  |  |  |  |  |  |  |  |  | Outlier: 1 |  |  |  |  |  |  |  |  |  |  |  |  |  |
| Seong et al., 2007 | Mungo 2 stage | m3I | K2-87 | 35.689 | 75.941 | 3442 | 3 | 2.7 | 0 | 0.98 | 48.83±1.81 | KNSTD |  | 13.52±0.73 | Wt. mean: 13.02±0.40 ka | 13.69±0.8 | 14.50±1.10 | 14.70±1.20 | 13.40±1.20 | 13.30±1.20 | 14.60±2.00 | 15.00±2.00 | 14.40±1.80 | 13.27±0.49 | | 13.21±0.49 | 13.04±0.48 |  |
| Seong et al., 2007 | Mungo 2 stage | m3I | K2-88 | 35.688 | 75.927 | 3447 | 3 | 2.7 | 0 | 0.98 | 52.44±1.81 | KNSTD |  | 14.4±0.75 | Ar. Mean: 13.06±0.40 ka | 14.56±0.82 | 15.40±1.10 | 15.60±1.30 | 14.30±1.20 | 14.20±1.30 | 15.50±2.20 | 15.80±2.20 | 15.30±1.90 | 13.98±0.48 | | 13.98±0.48 | 13.97±0.48 |  |
| Seong et al., 2007 | Mungo 2 stage | m3I | K2-89 | 35.688 | 75.927 | 3441 | 3 | 2.7 | 0 | 0.98 | 46.11±0.90 | KNSTD |  | 12.85±0.62 | Peak: 12.98 ka | 13.02±0.71 | 14.00±1.00 | 14.00±1.10 | 13.00±1.00 | 12.50±1.10 | 14.00±1.90 | 14.20±1.90 | 13.70±1.80 | 12.76±0.25 | | 12.65±0.25 | 12.32±0.24 |  |
| Seong et al., 2009 | Olimde 2 stage | m3H | KONG_5 | 38.642 | 74.995 | 3539 | 5 | 2.7 | 0 | 1 | 53.17±1.45 | KNSTD | 13.01±0.14 | 12.88±0.67 | Chi-squared: 0.04 | 12.48±0.75 | 13.00±1.00 | 13.50±1.10 | 12.80±1.10 | 12.70±1.10 | 14.00±1.90 | 13.90±1.90 | 13.30±1.80 | 12.50±0.34 | | 12.68±0.35 | 12.43±0.34 |  |
|  |  |  |  |  |  |  |  |  |  |  |  |  |  |  | Skewness: +0.54 |  |  |  |  |  |  |  |  |  |  |  |  |  |
|  |  |  |  |  |  |  |  |  |  |  |  |  |  |  | Outlier: 0 |  |  |  |  |  |  |  |  |  |  |  |  |  |
|  |  |  |  |  |  |  |  |  |  |  |  |  |  |  | Wt. mean: 13.01±0.14 ka |  |  |  |  |  |  |  |  |  |  |  |  |  |
| Seong et al., 2009 | Olimde 2 stage | m3H | KONG_6 | 38.642 | 74.995 | 3539 | 5 | 2.7 | 0 | 1 | 53.71±1.45 | KNSTD |  | 12.99±0.66 | Ar. Mean: 13.01±0.14 ka | 12.6±0.76 | 13.00±1.00 | 13.60±1.10 | 12.90±1.10 | 12.80±1.10 | 14.00±1.90 | 14.00±1.90 | 13.50±1.80 | 12.60±0.34 | | 12.78±0.35 | 12.55±0.34 |  |
| Seong et al., 2009 | Olimde 2 stage | m3H | KONG_7 | 38.642 | 74.995 | 3542 | 5 | 2.7 | 0 | 1 | 54.61±1.45 | KNSTD |  | 13.15±0.66 | Peak: 13.01 ka | 12.8±0.76 | 14.00±1.00 | 13.80±1.10 | 13.10±1.10 | 13.00±1.10 | 14.00±1.90 | 14.20±1.90 | 13.70±1.80 | 12.74±0.34 | | 12.93±0.34 | 12.74±0.34 |  |
| Owen et al., 2002 | Batura stage | t6 | KK98-1 | 36.46 | 74.9 | 2550 | 3 | 2.7 | 0 | 1 | 42.30±0.90 | LLNL3000 | 12.49±1.05 | 16.51±0.82 | Chi-squared: ! | 17.08±0.94 | 18.00±1.30 | 18.10±1.40 | 16.50±1.30 | 16.50±1.30 | 18.60±2.60 | 18.70±2.50 | 18.40±2.30 | 16.46±0.39 | | 16.02±0.38 | 16.21±0.39 |  |
|  |  |  |  |  |  |  |  |  |  |  |  |  |  |  | Skewness: ! |  |  |  |  |  |  |  |  |  |  |  |  |  |
|  |  |  |  |  |  |  |  |  |  |  |  |  |  |  | Outlier: 2 |  |  |  |  |  |  |  |  |  |  |  |  |  |
| Owen et al., 2002 | Batura stage | t6 | KK98-2 | 36.46 | 74.9 | 2550 | 3 | 2.7 | 0 | 1 | 29.50±0.90 | LLNL3000 |  | 11.75±0.65 | Wt. mean: 12.23±1.05 ka | 12.29±0.78 | 13.10±1.10 | 13.10±1.20 | 12.00±1.10 | 12.00±1.10 | 13.00±2.00 | 13.70±1.90 | 13.20±1.80 | 12.20±0.42 | | 11.73±0.40 | 11.29±0.42 |  |
| Owen et al., 2002 | Batura stage | t6 | KK98-3 | 36.46 | 74.9 | 2550 | 3 | 2.7 | 0 | 1 | 47.10±1.80 | LLNL3000 |  | 18.27±1.06 | Ar. Mean: 12.49±1.05 ka | 18.81±1.17 | 19.70±1.40 | 19.80±1.50 | 18.30±1.40 | 18.30±1.40 | 20.40±2.70 | 20.40±2.60 | 20.10±2.40 | 18.18±0.78 | | 17.71±0.76 | 18.06±0.78 |  |
| Owen et al., 2002 | Batura stage | t6 | KK98-4 | 36.46 | 74.9 | 2550 | 3 | 2.7 | 0 | 1 | 33.30±1.90 | LLNL3000 |  | 13.23±0.94 | Peak: 11.93 | 13.74±1.01 | 14.60±1.30 | 14.60±1.40 | 13.10±1.30 | 13.10±1.30 | 15.00±2.20 | 15.20±2.10 | 15.00±2.00 | 13.37±0.81 | | 12.96±0.78 | 12.75±0.81 |  |
| Seong et al., 2007 | Mungo 2 stage | m1E | K2-31 | 35.721 | 75.676 | 3164 | 2 | 2.7 | 0 | 0.94 | 36.17±0.90 | KNSTD | 12.41±0.33 | 12.18±0.65 | Chi-squared: 0.25 | 12.52±0.74 | 13.00±1.00 | 13.40±1.10 | 12.05±0.97 | 12.00±1.00 | 13.00±1.90 | 13.80±1.90 | 13.20±1.80 | 12.36±0.31 | | 12.09±0.30 | 11.67±0.29 |  |
|  |  |  |  |  |  |  |  |  |  |  |  |  |  |  | Skewness: ! |  |  |  |  |  |  |  |  |  |  |  |  |  |
|  |  |  |  |  |  |  |  |  |  |  |  |  |  |  | Outlier: 1 |  |  |  |  |  |  |  |  |  |  |  |  |  |
|  |  |  |  |  |  |  |  |  |  |  |  |  |  |  | Wt. mean: 12.41±0.33 ka |  |  |  |  |  |  |  |  |  |  |  |  |  |
| Seong et al., 2007 | Mungo 2 stage | m1E | K2-32 | 35.722 | 75.674 | 3166 | 3 | 2.7 | 0 | 0.94 | 5.43±0.90 | KNSTD |  | 1.99±0.36 | Ar. Mean: 12.41±0.33 ka | 2.11±0.4 | 2.23±0.47 | 2.24±0.48 | 1.96±0.41 | 1.79±0.36 | 2.23±0.53 | 2.50±0.53 | 2.22±0.53 | 1.93±0.32 | | 1.86±0.31 | 1.76±0.29 |  |
| Seong et al., 2007 | Mungo 2 stage | m1E (bedrock) | K2-33 | 35.724 | 75.672 | 3052 | 4 | 2.7 | 0 | 0.96 | 35.26±0.90 | KNSTD |  | 12.64±0.65 | Peak: 12.41 ka | 12.99±0.75 | 14.00±1.00 | 13.90±1.10 | 12.00±1.00 | 12.30±1.10 | 14.00±1.90 | 14.30±1.90 | 13.80±1.80 | 12.73±0.33 | | 12.46±0.32 | 12.10±0.31 |  |
| Seong et al., 2009 | Olimde 2 stage | m5C | MUST-72 | 38.287 | 75.011 | 4295 | 5 | 2.7 | 0 | 1 | 80.65±1.99 | KNSTD | 11.71±0.40 | 13.2±0.64 | Chi-squared: 0.46 | 12.49±0.74 | 13.00±1.00 | 14.00±1.00 | 13.20±1.10 | 13.00±1.10 | 13.00±1.90 | 13.70±1.90 | 13.00±1.70 | 12.50±0.31 | | 12.97±0.32 | 12.79±0.32 |  |
|  |  |  |  |  |  |  |  |  |  |  |  |  |  |  | Skewness: ! |  |  |  |  |  |  |  |  |  |  |  |  |  |
|  |  |  |  |  |  |  |  |  |  |  |  |  |  |  | Outlier: 2 |  |  |  |  |  |  |  |  |  |  |  |  |  |
| Seong et al., 2009 | Olimde 2 stage | m5C | MUST-73 | 38.286 | 75.011 | 4298 | 5 | 2.7 | 0 | 1 | 69.62±1.72 | KNSTD |  | 11.43±0.54 | Wt. mean: 11.66±0.40 ka | 10.92±0.56 | 11.60±0.89 | 12.27±0.91 | 11.37±0.95 | 11.24±0.94 | 11.60±1.60 | 12.00±1.60 | 11.40±1.50 | 11.22±0.28 | | 11.50±0.29 | 11.02±0.27 |  |
| Seong et al., 2009 | Olimde 2 stage | m5C | MUST-74 | 38.286 | 75.011 | 4292 | 5 | 2.7 | 0 | 1 | 72.97±1.81 | KNSTD |  | 11.99±0.64 | Ar. Mean: 11.71±0.40 ka | 11.34±0.59 | 12.13±0.88 | 12.85±0.99 | 11.94±0.97 | 11.81±0.99 | 12.10±1.70 | 12.50±1.70 | 11.90±1.50 | 11.61±0.29 | | 11.97±0.30 | 11.59±0.29 |  |
| Seong et al., 2009 | Olimde 2 stage | m5C | MUST-75 | 38.286 | 75.011 | 4293 | 5 | 2.7 | 0 | 1 | 74.14±2.89 | KNSTD |  | 12.19±0.73 | Peak: 11.62 ka | 11.48±0.69 | 12.30±0.98 | 13.00±1.10 | 12.00±1.00 | 12.00±1.10 | 12.30±1.80 | 12.70±1.80 | 12.10±1.60 | 11.73±0.46 | | 12.13±0.48 | 11.77±0.46 |  |
| Seong et al., 2009 | Olimde 3 stage | m3F | MUST-1 | 38.467 | 75.061 | 3689 | 5 | 2.7 | 0 | 1 | 45.30±1.18 | KNSTD | 10.25±0.16 | 10.31±0.5 | Chi-squared: 0.10 | 10.04±0.57 | 10.56±0.87 | 10.74±0.96 | 10.08±0.88 | 9.97±0.86 | 10.80±1.60 | 11.20±1.60 | 10.60±1.50 | 10.34±0.27 | | 10.40±0.27 | 9.79±0.26 |  |
| Seong et al., 2009 | Olimde 3 stage | m3F | MUST-2 | 38.467 | 75.062 | 3681 | 5 | 2.7 | 0 | 1 | 44.85±1.18 | KNSTD |  | 10.26±0.5 | Skewness: +1.26 | 10±0.57 | 10.50±0.86 | 10.68±0.95 | 10.02±0.87 | 9.91±0.85 | 10.70±1.60 | 11.10±1.60 | 10.50±1.50 | 10.30±0.27 | | 10.34±0.27 | 9.73±0.26 |  |
| Seong et al., 2009 | Olimde 3 stage | m3F | MUST-3 | 38.465 | 75.065 | 3700 | 5 | 2.7 | 0 | 1 | 46.66±1.45 | KNSTD |  | 10.54±0.53 | Outlier: 0 | 10.22±0.59 | 10.81±0.91 | 11.00±1.00 | 10.33±0.92 | 10.20±0.90 | 11.00±1.60 | 11.40±1.60 | 10.80±1.50 | 10.60±0.33 | | 10.66±0.33 | 10.02±0.31 |  |
| Seong et al., 2009 | Olimde 3 stage | m3F | MUST-4 | 38.464 | 75.065 | 3709 | 5 | 2.7 | 0 | 1 | 45.12±1.18 | KNSTD |  | 10.18±0.5 | Wt. mean: 10.25±0.16 ka | 9.91±0.57 | 10.40±0.87 | 10.58±0.94 | 9.93±0.87 | 9.82±0.85 | 10.60±1.60 | 11.00±1.60 | 10.40±1.40 | 10.22±0.27 | | 10.27±0.27 | 9.65±0.25 |  |
| Seong et al., 2009 | Olimde 3 stage | m3F | MUST-5 | 38.466 | 75.062 | 3688 | 5 | 2.7 | 0 | 1 | 44.31±1.18 | KNSTD |  | 10.12±0.5 | Ar. Mean: 10.25±0.16 ka | 9.85±0.58 | 10.33±0.86 | 10.51±0.93 | 9.86±0.87 | 9.75±0.84 | 10.50±1.60 | 11.00±1.60 | 10.30±1.40 | 10.17±0.27 | | 10.21±0.27 | 9.58±0.26 |  |
| Seong et al., 2009 | Olimde 3 stage | m3F | MUST-6 | 38.467 | 75.061 | 3685 | 5 | 2.7 | 0 | 1 | 44.12±1.09 | KNSTD |  | 10.1±0.5 | Peak: 10.24 ka | 9.83±0.57 | 10.31±0.85 | 10.49±0.93 | 9.83±0.86 | 9.73±0.83 | 10.50±1.60 | 10.90±1.60 | 10.30±1.40 | 10.16±0.25 | | 10.19±0.25 | 9.56±0.24 |  |
| Seong et al., 2009 | Olimde 3 stage | m3F´ | MUST-7 | 38.511 | 75.034 | 3535 | 5 | 2.7 | 0 | 1 | 42.23±1.54 | KNSTD | 9.69±0.34 | 10.43±0.55 | Chi-squared: 0.43 | 10.2±0.62 | 10.76±0.95 | 11.00±1.00 | 10.22±0.93 | 10.10±0.92 | 11.00±1.60 | 11.40±1.60 | 10.80±1.50 | 10.55±0.39 | | 10.54±0.39 | 9.92±0.36 |  |
| Seong et al., 2009 | Olimde 3 stage | m3F´ | MUST-8 | 38.516 | 75.032 | 3534 | 5 | 2.7 | 0 | 1 | 41.32±1.09 | KNSTD |  | 10.23±0.51 | Skewness: +0.21 | 10.02±0.58 | 10.54±0.87 | 10.71±0.94 | 10.00±0.87 | 9.89±0.85 | 10.80±1.60 | 11.20±1.60 | 10.60±1.50 | 10.32±0.27 | | 10.32±0.27 | 9.71±0.26 |  |
| Seong et al., 2009 | Olimde 3 stage | m3F´ | MUST-9 | 38.52 | 75.031 | 3521 | 5 | 2.7 | 0 | 1 | 39.69±0.99 | KNSTD |  | 9.94±0.49 | Outlier: 1 | 9.73±0.58 | 10.20±0.82 | 10.36±0.92 | 9.66±0.86 | 9.56±0.82 | 10.50±1.60 | 10.90±1.60 | 10.30±1.40 | 10.08±0.25 | | 10.06±0.25 | 9.39±0.24 |  |
| Seong et al., 2009 | Olimde 3 stage | m3F´ | MUST-10 | 38.521 | 75.032 | 3517 | 5 | 2.7 | 0 | 1 | 36.89±0.99 | KNSTD |  | 9.25±0.54 | Wt. mean: 9.71±0.34 ka | 8.99±0.63 | 9.49±0.79 | 9.60±0.90 | 8.96±0.79 | 8.91±0.77 | 9.70±1.50 | 10.20±1.50 | 9.60±1.40 | 9.52±0.26 | | 9.44±0.26 | 8.75±0.24 |  |
| Seong et al., 2009 | Olimde 3 stage | m3F´ | MUST-11 | 38.52 | 75.034 | 3494 | 5 | 2.7 | 0 | 1 | 36.62±0.99 | KNSTD |  | 9.31±0.54 | Ar. Mean: 9.69±0.34 ka | 9.06±0.63 | 9.55±0.81 | 9.71±0.91 | 9.00±0.80 | 8.96±0.78 | 9.80±1.50 | 10.20±1.50 | 9.60±1.40 | 9.58±0.26 | | 9.49±0.26 | 8.79±0.24 |  |
| Seong et al., 2009 | Olimde 3 stage | m3F´ | MUST-12 | 38.521 | 75.034 | 3490 | 5 | 2.7 | 0 | 1 | 38.34±0.99 | KNSTD |  | 9.78±0.5 | Peak: 9.70 ka | 9.56±0.61 | 10.04±0.85 | 10.20±0.87 | 9.48±0.84 | 9.40±0.81 | 10.30±1.60 | 10.70±1.60 | 10.10±1.40 | 9.98±0.26 | | 9.92±0.26 | 9.23±0.24 |  |
| Seong et al., 2009 | Olimde 3 stage | m3F´ | MUST-13 | 38.514 | 75.035 | 3511 | 5 | 2.7 | 0 | 1 | 38.07±1.27 | KNSTD |  | 9.6±0.56 |  | 9.37±0.66 | 9.84±0.88 | 10.00±0.94 | 9.30±0.85 | 9.22±0.82 | 10.10±1.60 | 10.50±1.60 | 9.90±1.40 | 9.83±0.33 | | 9.77±0.33 | 9.06±0.30 |  |
| Seong et al., 2009 | Olimde 3 stage | m3F´ | MUST-14 | 38.513 | 75.035 | 3521 | 5 | 2.7 | 0 | 1 | 38.70±0.99 | KNSTD |  | 9.71±0.51 |  | 9.48±0.62 | 9.95±0.85 | 10.10±0.90 | 9.40±0.83 | 9.30±0.80 | 10.20±1.60 | 10.60±1.60 | 10.00±1.40 | 9.92±0.26 | | 9.86±0.25 | 9.16±0.24 |  |
| Seong et al., 2009 | Olimde 4 Stage | m4H | KONG_8 | 38.64 | 75.003 | 3639 | 5 | 2.7 | 0 | 0.98 | 33.55±0.99 | KNSTD | 7.98±0.10 | 7.95±0.43 | Chi-squared: 0.06 | 7.68±0.44 | 8.14±0.64 | 8.30±0.70 | 7.72±0.65 | 7.70±0.67 | 8.30±1.30 | 8.80±1.30 | 8.20±1.10 | 8.03±0.24 | | 8.08±0.24 | 7.56±0.23 |  |
| Seong et al., 2009 | Olimde 4 Stage | m4H | KONG_9 | 38.64 | 75.004 | 3637 | 5 | 2.7 | 0 | 0.98 | 33.09±0.99 | KNSTD |  | 7.86±0.42 | Skewness: +1.21 | 7.59±0.43 | 8.05±0.62 | 8.17±0.67 | 7.63±0.64 | 7.60±0.67 | 8.20±1.30 | 8.70±1.30 | 8.10±1.10 | 7.92±0.24 | | 7.98±0.24 | 7.47±0.23 |  |
| Seong et al., 2009 | Olimde 4 Stage | m4H | KONG_10 | 38.64 | 75.004 | 3638 | 5 | 2.7 | 0 | 0.98 | 33.82±0.99 | KNSTD |  | 8.01±0.44 | Outlier: 0 | 7.74±0.44 | 8.21±0.65 | 8.34±0.73 | 7.79±0.66 | 7.77±0.68 | 8.40±1.30 | 8.90±1.30 | 8.30±1.10 | 8.10±0.24 | | 8.15±0.24 | 7.63±0.23 |  |
| Seong et al., 2009 | Olimde 4 Stage | m4H | KONG_11 | 38.641 | 75.0047 | 3639 | 5 | 2.7 | 0 | 0.98 | 33.64±1.09 | KNSTD |  | 7.97±0.44 | Wt. mean: 7.98±0.10 ka | 7.71±0.45 | 8.16±0.63 | 8.29±0.75 | 7.74±0.66 | 7.72±0.68 | 8.40±1.30 | 8.80±1.30 | 8.20±1.10 | 8.05±0.26 | | 8.10±0.26 | 7.58±0.25 |  |
| Seong et al., 2009 | Olimde 4 Stage | m4H | KONG_12 | 38.641 | 75.0052 | 3641 | 5 | 2.7 | 0 | 0.98 | 34.54±0.99 | KNSTD |  | 8.17±0.44 | Ar. Mean: 7.98±0.10 ka | 7.9±0.46 | 8.36±0.66 | 8.50±0.79 | 7.94±0.67 | 7.92±0.69 | 8.60±1.30 | 9.00±1.30 | 8.40±1.20 | 8.25±0.24 | | 8.30±0.24 | 7.78±0.22 |  |
| Seong et al., 2009 | Olimde 4 Stage | m4H | KONG_13 | 38.641 | 75.0057 | 3652 | 5 | 2.7 | 0 | 0.98 | 33.73±1.63 | KNSTD |  | 7.94±0.51 | Peak: 7.99 ka | 7.66±0.5 | 8.12±0.72 | 8.25±0.78 | 7.71±0.73 | 7.69±0.75 | 8.30±1.30 | 8.80±1.30 | 8.20±1.10 | 8.01±0.39 | | 8.07±0.39 | 7.55±0.37 |  |
| Seong et al., 2009 | Olimde 4 Stage | m6A | MUST-27 | 38.364 | 75.171 | 4023 | 5 | 2.7 | 0 | 0.99 | 38.97±1.18 | KNSTD | 7.80±0.29 | 7.52±0.39 | Chi-squared: 0.48 | 7.26±0.38 | 7.65±0.55 | 7.77±0.63 | 7.30±0.62 | 7.27±0.64 | 7.70±1.10 | 8.20±1.10 | 7.58±0.98 | 7.45±0.23 | | 7.60±0.23 | 7.14±0.22 |  |
|  |  |  |  |  |  |  |  |  |  |  |  |  |  |  | Skewness: +0.65 |  |  |  |  |  |  |  |  |  |  |  |  |  |
| Seong et al., 2009 | Olimde 4 Stage | m6A | MUST-28 | 38.364 | 75.171 | 4024 | 5 | 2.7 | 0 | 0.99 | 41.41±1.09 | KNSTD |  | 7.98±0.42 | Outlier: 0 | 7.61±0.42 | 8.07±0.61 | 8.21±0.68 | 7.74±0.64 | 7.72±0.67 | 8.20±1.20 | 8.60±1.20 | 8.00±1.10 | 7.96±0.21 | | 8.12±0.21 | 7.59±0.20 |  |
| Seong et al., 2009 | Olimde 4 Stage | m6A | MUST-29 | 38.364 | 75.171 | 4025 | 5 | 2.7 | 0 | 0.99 | 40.06±1.09 | KNSTD |  | 7.72±0.4 | Wt. mean: 7.77±0.29 ka | 7.4±0.39 | 7.82±0.59 | 7.96±0.62 | 7.49±0.62 | 7.46±0.65 | 7.90±1.20 | 8.40±1.20 | 8.00±1.00 | 7.64±0.21 | | 7.84±0.21 | 7.33±0.20 |  |
| Seong et al., 2009 | Olimde 4 Stage | m6A | MUST-30 | 38.364 | 75.171 | 4026 | 5 | 2.7 | 0 | 0.98 | 42.23±1.09 | KNSTD |  | 8.2±0.44 | Ar. Mean: 7.80±0.29 ka | 7.84±0.44 | 8.29±0.68 | 8.44±0.73 | 7.96±0.66 | 7.94±0.68 | 8.40±1.30 | 8.90±1.30 | 8.20±1.10 | 8.20±0.21 | | 8.34±0.22 | 7.81±0.20 |  |
| Seong et al., 2009 | Olimde 4 Stage | m6A | MUST-31 | 38.363 | 75.171 | 4028 | 5 | 2.7 | 0 | 0.98 | 38.97±0.99 | KNSTD |  | 7.57±0.38 | Peak: 7.72 ka | 7.3±0.37 | 7.70±0.57 | 7.83±0.63 | 7.30±0.60 | 7.32±0.63 | 7.80±1.10 | 8.20±1.10 | 7.63±0.98 | 7.50±0.19 | | 7.67±0.20 | 7.20±0.18 |  |
| Seong et al., 2009 | Olimde 4 Stage | m5A | MUST-21 | 38.393 | 75.171 | 3862 | 5 | 2.7 | 0 | 1 | 39.15±0.99 | KNSTD | 7.74±0.27 | 8.11±0.43 | Chi-squared: 0.41 | 7.79±0.44 | 8.25±0.64 | 8.40±0.72 | 7.88±0.66 | 7.86±0.68 | 8.40±1.30 | 8.90±1.30 | 8.20±1.10 | 8.17±0.21 | | 8.26±0.21 | 7.72±0.20 |  |
| Seong et al., 2009 | Olimde 4 Stage | m5A | MUST-22 | 38.393 | 75.172 | 3864 | 5 | 2.7 | 0 | 1 | 36.62±0.99 | KNSTD |  | 7.6±0.39 | Skewness: +0.12 | 7.35±0.38 | 7.76±0.56 | 7.88±0.66 | 7.37±0.61 | 7.34±0.64 | 7.90±1.20 | 8.30±1.20 | 8.00±1.00 | 7.55±0.21 | | 7.69±0.21 | 7.22±0.20 |  |
| Seong et al., 2009 | Olimde 4 Stage | m5A | MUST-23 | 38.393 | 75.172 | 3862 | 5 | 2.7 | 0 | 1 | 35.63±1.27 | KNSTD |  | 7.4±0.4 | Outlier: 0 | 7.19±0.39 | 7.58±0.57 | 7.70±0.64 | 7.18±0.63 | 7.15±0.65 | 7.70±1.10 | 8.10±1.10 | 7.54±0.98 | 7.39±0.26 | | 7.48±0.27 | 7.03±0.25 |  |
| Seong et al., 2009 | Olimde 4 Stage | m5A | MUST-24 | 38.393 | 75.171 | 3862 | 5 | 2.7 | 0 | 1 | 36.44±1.36 | KNSTD |  | 7.56±0.43 | Wt. mean: 7.74±0.27 ka | 7.33±0.42 | 7.70±0.60 | 7.86±0.65 | 7.34±0.64 | 7.31±0.67 | 7.80±1.20 | 8.30±1.20 | 8.00±1.00 | 7.52±0.28 | | 7.65±0.29 | 7.19±0.27 |  |
| Seong et al., 2009 | Olimde 4 Stage | m5A | MUST-25 | 38.393 | 75.171 | 3862 | 5 | 2.7 | 0 | 1 | 37.89±0.99 | KNSTD |  | 7.87±0.41 | Ar. Mean: 7.74±0.27 ka | 7.56±0.42 | 8.00±0.60 | 8.14±0.68 | 7.63±0.63 | 7.60±0.66 | 8.10±1.20 | 8.60±1.20 | 8.00±1.10 | 7.88±0.21 | | 7.99±0.21 | 7.47±0.20 |  |
| Seong et al., 2009 | Olimde 4 Stage | m5A | MUST-26 | 38.393 | 75.172 | 3861 | 5 | 2.7 | 0 | 1 | 38.16±0.99 | KNSTD |  | 7.92±0.41 | Peak: 7.73 ka | 7.61±0.42 | 8.06±0.61 | 8.20±0.69 | 7.69±0.64 | 7.66±0.66 | 8.20±1.30 | 8.60±1.30 | 8.00±1.10 | 7.95±0.21 | | 8.06±0.21 | 7.53±0.20 |  |
| Seong et al., 2007 | Mungo 2 stage | m2G | K2-65 | 35.672 | 75.814 | 3113 | 3 | 2.7 | 0 | 0.8 | 36.17±0.90 | KNSTD | 6.64±0.35 | 14.7±0.7 | Chi-squared: 0.68 | 15±0.81 | 15.90±1.20 | 16.10±1.30 | 14.60±1.10 | 14.50±1.20 | 16.10±2.30 | 16.40±2.30 | 15.90±2.10 | 14.44±0.36 | | 14.24±0.36 | 14.25±0.36 |  |
| Seong et al., 2007 | Mungo 2 stage | m2G | K2-66 | 35.672 | 75.814 | 3128 | 3 | 2.7 | 0 | 0.8 | 14.47±0.90 | KNSTD |  | 6.17±0.41 | Skewness: -1.13 | 6.42±0.44 | 6.54±0.76 | 6.62±0.82 | 5.88±0.58 | 5.76±0.61 | 6.60±1.00 | 7.00±1.00 | 6.50±1.10 | 6.54±0.41 | | 6.19±0.39 | 5.64±0.35 |  |
| Seong et al., 2007 | Mungo 2 stage | m2G | K2-67 | 35.672 | 75.814 | 3122 | 5 | 2.7 | 0 | 0.8 | 15.37±0.90 | KNSTD |  | 6.6±0.44 | Outlier: 2 | 6.86±0.46 | 7.14±0.64 | 7.20±0.68 | 6.33±0.58 | 6.24±0.65 | 7.20±1.10 | 7.60±1.10 | 7.00±1.00 | 7.00±0.41 | | 6.67±0.39 | 6.11±0.36 |  |
| Seong et al., 2007 | Mungo 2 stage | m2G | K2-68 | 35.672 | 75.814 | 3113 | 3 | 2.7 | 0 | 0.8 | 16.28±0.90 | KNSTD |  | 6.87±0.44 | Wt. mean: 6.63±0.35 ka | 7.14±0.47 | 7.44±0.64 | 7.50±0.68 | 6.60±0.60 | 6.53±0.67 | 7.50±1.10 | 7.90±1.10 | 7.00±1.00 | 7.25±0.40 | | 6.95±0.39 | 6.40±0.36 |  |
| Seong et al., 2007 | Mungo 2 stage | m2G | K2-69 | 35.703 | 75.954 | 3109 | 3.5 | 2.7 | 0 | 0.8 | 18.99±0.90 | KNSTD |  | 7.99±0.52 | Ar. Mean: 6.64±0.35 ka | 8.23±0.62 | 8.64±0.85 | 8.73±0.91 | 7.67±0.71 | 7.67±0.74 | 8.70±1.50 | 9.20±1.50 | 8.60±1.30 | 8.47±0.40 | | 8.09±0.39 | 7.51±0.36 |  |
| Seong et al., 2007 | Mungo 2 stage | m2G | K2-70 | 35.703 | 75.952 | 3106 | 2 | 2.7 | 0 | 0.79 | 16.28±0.90 | KNSTD |  | 6.93±0.44 | Peak: 6.68 ka | 7.18±0.47 | 7.49±0.65 | 7.55±0.68 | 6.64±0.58 | 6.58±0.67 | 7.60±1.20 | 8.00±1.20 | 7.00±1.00 | 7.29±0.41 | | 6.99±0.39 | 6.45±0.36 |  |
| Seong et al., 2007 | Askole 2 stage | m2b | K2-116 | 35.292 | 75.662 | 2276 | 4 | 2.7 | 0 | 1 | 10.85±1.81 | KNSTD | 5.98±0.69 | 6.77±1.01 | Chi-squared: 0.92 | 6.33±0.95 | 7.00±1.30 | 7.00±1.30 | 6.10±1.10 | 5.90±1.20 | 7.20±1.50 | 7.50±1.50 | 7.10±1.50 | 6.89±1.15 | | 6.38±1.06 | 5.83±0.97 |  |
|  |  |  |  |  |  |  |  |  |  |  |  |  |  |  | Skewness: +0.33 |  |  |  |  |  |  |  |  |  |  |  |  |  |
|  |  |  |  |  |  |  |  |  |  |  |  |  |  |  | Outlier: 0 |  |  |  |  |  |  |  |  |  |  |  |  |  |
| Seong et al., 2007 | Askole 2 stage | m2b | K2-117 | 35.292 | 75.662 | 2276 | 5 | 2.7 | 0 | 1 | 8.14±1.00 | KNSTD |  | 5.40±0.61 | Wt. mean: 5.83±0.69 ka | 4.97±0.59 | 5.35±0.33 | 5.35±0.36 | 4.68±0.35 | 4.50±0.36 | 5.44±0.66 | 5.66±0.66 | 5.40±0.61 | 0.00±0.00 | | 0.00±0.00 | 0.00±0.00 |  |
| Seong et al., 2007 | Askole 2 stage | m2b | K2-118 | 35.315 | 75.626 | 2276 | 5 | 2.7 | 0 | 1 | 9.95±1.00 | KNSTD |  | 6.34±0.61 | Ar. Mean: 5.98±0.69 ka | 5.90±0.56 | 6.46±0.53 | 6.45±0.58 | 5.60±0.40 | 5.49±0.44 | 6.60±1.10 | 7.00±1.10 | 6.56±0.98 | 0.00±0.00 | | 0.00±0.00 | 0.00±0.00 |  |
| Seong et al., 2007 | Askole 2 stage | m2b | K2-119 | 35.314 | 75.625 | 2276 | 5 | 2.7 | 0 | 1 | 8.14±1.00 | KNSTD |  | 5.40±0.61 | Peak: 5.68 ka | 4.97±0.58 | 5.35±0.33 | 5.35±0.36 | 4.68±0.35 | 4.49±0.36 | 5.44±0.66 | 5.66±0.66 | 5.40±0.61 | 0.00±0.00 | | 0.00±0.00 | 0.00±0.00 |  |
| Koppes et al. (2008) | Aksai | N/A | KTS98-CS-61b | 41 | 76.05 | 3804 | 3 | 2.7 | 0 | 0.99 | 50.70±1.50 | 07KNSTD | 5.70±0.16 | 9.24±0.64 | Chi-squared: ! | 10.03±0.52 | 9.77±0.84 | 9.97±0.91 | 9.77±0.84 | 9.86±0.87 | 10.20±1.60 | 10.60±1.50 | 10.00±1.40 | 9.85±0.29 | | 10.15±0.30 | 9.60±0.29 |  |
|  |  |  |  |  |  |  |  |  |  |  |  |  |  |  | Skewness: ! |  |  |  |  |  |  |  |  |  |  |  |  |  |
|  |  |  |  |  |  |  |  |  |  |  |  |  |  |  | Outlier: 2 |  |  |  |  |  |  |  |  |  |  |  |  |  |
| Koppes et al. (2008) | Aksai | N/A | KTS98-CS-62a | 41 | 76.05 | 3879 | 3 | 2.7 | 0 | 0.99 | 32.00±1.00 | 07KNSTD |  | 5.81±0.31 | Wt. mean: 5.69±0.16 ka | 6.19±0.31 | 5.86±0.49 | 5.99±0.66 | 5.92±0.51 | 6.00±0.50 | 6.10±1.10 | 6.60±1.20 | 5.94±0.91 | 6.01±0.19 | | 6.25±0.20 | 5.82±0.18 |  |
| Koppes et al. (2008) | Aksai | N/A | KTS98-CS-62b | 41 | 76.05 | 3879 | 3 | 2.7 | 0 | 0.989 | 30.50±0.90 | 07KNSTD |  | 5.58±0.29 | Ar. Mean: 5.70±0.16 ka | 5.93±0.29 | 5.60±0.36 | 5.70±0.46 | 5.64±0.49 | 5.74±0.45 | 5.80±0.90 | 6.20±1.20 | 5.67±0.72 | 5.69±0.17 | | 5.94±0.18 | 5.54±0.16 |  |
| Koppes et al. (2008) | Aksai | N/A | KTS98-CS-66 | 40.98 | 76.15 | 3576 | 4 | 2.7 | 0 | 0.996 | 205.80±3.50 | 07KNSTD |  | 39.33±2.00 | Peak: 5.68 ka | 42.39±1.97 | 39.30±2.80 | 40.00±3.00 | 45.40±3.80 | 41.90±3.10 | 42.00±6.00 | 41.70±5.90 | 41.00±5.00 | 38.49±0.66 | | 41.39±0.71 | 44.56±0.77 |  |
| Seong et al., 2009 | Olimde 5 stage | m6C´ | MUST-76 | 38.292 | 75.025 | 4477 | 5 | 2.7 | 0 | 0.99 | 36.08±0.81 | KNSTD | 5.05±0.14 | 5.75±0.26 | Chi-squared: 0.33 | 5.59±0.26 | 5.53±0.31 | 5.61±0.36 | 5.51±0.44 | 5.36±0.46 | 5.50±0.82 | 5.83±0.82 | 5.40±0.62 | 5.61±0.13 | | 5.72±0.13 | 5.27±0.12 |  |
|  |  |  |  |  |  |  |  |  |  |  |  |  |  |  | Skewness: +1.69 |  |  |  |  |  |  |  |  |  |  |  |  |  |
|  |  |  |  |  |  |  |  |  |  |  |  |  |  |  | Outlier: 1 |  |  |  |  |  |  |  |  |  |  |  |  |  |
| Seong et al., 2009 | Olimde 5 stage | m6C´ | MUST-77 | 38.292 | 75.025 | 4473 | 5 | 2.7 | 0 | 0.99 | 30.47±0.90 | KNSTD |  | 4.98±0.26 | Wt. mean: 5.06±0.14 ka | 4.88±0.28 | 4.85±0.36 | 4.93±0.38 | 4.71±0.38 | 4.50±0.40 | 4.77±0.58 | 5.18±0.58 | 4.67±0.67 | 4.68±0.14 | | 4.82±0.14 | 4.46±0.13 |  |
| Seong et al., 2009 | Olimde 5 stage | m6C´ | MUST-78 | 38.293 | 75.024 | 4461 | 5 | 2.7 | 0 | 0.99 | 31.92±0.90 | KNSTD |  | 5.22±0.25 | Ar. Mean: 5.05±0.14 ka | 5.12±0.27 | 5.07±0.35 | 5.16±0.38 | 4.94±0.38 | 4.78±0.42 | 5.00±0.57 | 5.37±0.57 | 4.91±0.63 | 5.02±0.14 | | 5.14±0.15 | 4.70±0.13 |  |
| Seong et al., 2009 | Olimde 5 stage | m6C´ | MUST-79 | 38.293 | 75.025 | 4473 | 5 | 2.7 | 0 | 0.99 | 30.38±0.90 | KNSTD |  | 4.96±0.25 | Peak: 5.05 ka | 4.87±0.27 | 4.83±0.36 | 4.92±0.38 | 4.70±0.39 | 4.50±0.40 | 4.76±0.59 | 5.17±0.59 | 4.66±0.66 | 4.66±0.14 | | 4.81±0.14 | 4.45±0.13 |  |
| Hedrick et al., 2011 | KM-4 stage | mG2 | TM-B | 32.971 | 78.182 | 5306 | 2 | 2.7 | 0 | 1 | 47.04±8.73 | 07KNSTD | 4.66±1.17 | 5.86±0.95 | Chi-squared: 13.12 | 6±0.91 | 5.80±1.10 | 5.90±1.30 | 6.00±1.00 | 5.40±1.10 | 5.00±1.20 | 5.80±1.20 | 5.41±0.95 | 6.08±1.13 | | 5.99±1.11 | 5.27±0.98 |  |
| Hedrick et al., 2011 | KM-4 stage | mG2 | TM-C | 32.971 | 78.182 | 5309 | 3 | 2.7 | 0 | 1 | 207.93±15.08 | 07KNSTD |  | 22.93±1.71 | Skewness: +0.03 | 22.14±1.58 | 23.00±2.10 | 23.40±2.20 | 22.80±2.30 | 23.90±2.60 | 21.90±3.00 | 22.00±3.00 | 21.50±2.70 | 21.38±1.56 | | 22.38±1.63 | 23.56±1.72 |  |
| Hedrick et al., 2011 | KM-4 stage | mG2 | TM-D | 32.971 | 78.182 | 5309 | 3 | 2.7 | 0 | 1 | 33.21±6.28 | 07KNSTD |  | 4.35±0.78 | Outlier: 2 | 4.54±0.81 | 4.34±0.98 | 4.00±1.00 | 4.07±0.75 | 3.80±0.78 | 3.90±1.20 | 4.40±1.20 | 3.89±0.98 | 4.30±0.82 | | 4.27±0.81 | 3.75±0.71 |  |
| Hedrick et al., 2011 | KM-4 stage | mG2 | TM-F | 32.971 | 78.181 | 5318 | 1 | 2.7 | 0 | 1 | 26.54±3.79 | 07KNSTD |  | 3.45±0.49 | Wt. mean: 4.43±1.17 ka | 3.59±0.52 | 3.34±0.52 | 3.41±0.56 | 3.28±0.51 | 2.98±0.49 | 3.11±0.64 | 3.43±0.64 | 3.08±0.54 | 3.33±0.48 | | 3.34±0.48 | 2.93±0.42 |  |
| Saha et al. (2018) | Neoglacial | mG2 | KO-17 | 32.971 | 78.179 | 5331 | 3 | 2.7 | 0 | 0.984 | 23.57±0.97 | 07KNSTD |  | 3.29±0.23 | Ar. Mean: 4.66±1.17 ka | 3.16±0.23 | 3.07±0.25 | 3.12±0.25 | 3.02±0.23 | 2.72±0.25 | 2.90±0.44 | 3.16±0.44 | 2.88±0.29 | 2.67±0.11 | | 3.07±0.13 | 3.06±0.13 |  |
| Saha et al. (2018) | Neoglacial | mG2 | KO-18 | 32.971 | 78.178 | 5337 | 2 | 2.7 | 0 | 0.985 | 36.75±1.19 | 07KNSTD |  | 4.93±0.28 | Peak: ! | 4.74±0.28 | 4.80±0.39 | 4.90±0.40 | 4.45±0.34 | 4.19±0.37 | 4.38±0.69 | 4.85±0.69 | 4.32±0.66 | 4.12±0.13 | | 4.70±0.15 | 4.77±0.15 |  |
| Saha et al. (2018) | Neoglacial | mG2 | KO-1501 | 32.972 | 78.182 | 5327 | 1 | 2.7 | 0 | 0.99 | 47.97±1.22 | 07KNSTD |  | 6.05±0.3 |  | 5.92±0.3 | 5.89±0.46 | 6.01±0.54 | 5.61±0.41 | 5.42±0.46 | 5.52±0.84 | 5.84±0.84 | 5.45±0.58 | 5.33±0.14 | | 6.06±0.16 | 6.14±0.16 |  |
| Saha et al. (2018) | Neoglacial | mG2 | KO-1502 | 32.972 | 78.181 | 5334 | 2 | 2.7 | 0 | 0.99 | 105.93±1.35 | 07KNSTD |  | 12.37±0.7 |  | 12.42±0.7 | 13.12±0.99 | 13.40±1.10 | 12.25±0.93 | 12.06±0.99 | 12.10±1.70 | 12.60±1.70 | 11.90±1.50 | 11.86±0.15 | | 12.44±0.16 | 12.18±0.16 |  |
| Seong et al., 2009 | Olimde 6 stage | m5H | KONG_18 | 38.649 | 75.032 | 4057 | 5 | 2.7 | 0 | 1 | 22.33±0.72 | KNSTD | 4.32±0.11 | 4.43±0.24 | Chi-squared: 0.19 | 4.38±0.27 | 4.30±0.44 | 4.39±0.46 | 4.20±0.34 | 4.02±0.36 | 4.31±0.69 | 4.78±0.69 | 4.22±0.66 | 4.17±0.14 | | 4.23±0.14 | 3.95±0.13 |  |
|  |  |  |  |  |  |  |  |  |  |  |  |  |  |  | Skewness: +0.42 |  |  |  |  |  |  |  |  |  |  |  |  |  |
| Seong et al., 2009 | Olimde 6 stage | m5H | KONG_19 | 38.649 | 75.032 | 4057 | 5 | 2.7 | 0 | 1 | 21.70±0.81 | KNSTD |  | 4.31±0.25 | Outlier: 2 | 4.27±0.28 | 4.16±0.43 | 4.25±0.45 | 4.09±0.34 | 3.90±0.36 | 4.18±0.76 | 4.65±0.76 | 4.09±0.66 | 4.06±0.15 | | 4.12±0.16 | 3.84±0.14 |  |
| Seong et al., 2009 | Olimde 6 stage | m5H | KONG_20 | 38.649 | 75.032 | 4060 | 5 | 2.7 | 0 | 1 | 21.25±0.81 | KNSTD |  | 4.22±0.25 | Wt. mean: 4.32±0.11 ka | 4.17±0.28 | 4.05±0.42 | 4.14±0.45 | 4.00±0.33 | 3.82±0.35 | 4.08±0.79 | 4.54±0.79 | 3.99±0.63 | 3.98±0.15 | | 4.04±0.16 | 3.75±0.14 |  |
| Seong et al., 2009 | Olimde 6 stage | m5H | KONG_21 | 38.645 | 75.031 | 4039 | 5 | 2.7 | 0 | 1 | 5.43±0.54 | KNSTD |  | 1.06±0.12 | Ar. Mean: 4.32±0.11 ka | 1.02±0.12 | 1.15±0.11 | 1.16±0.13 | 1.12±0.13 | 0.99±0.14 | 1.15±0.20 | 1.20±0.20 | 1.14±0.16 | 1.05±0.11 | | 1.06±0.11 | 0.97±0.10 |  |
| Seong et al., 2009 | Olimde 6 stage | m5H | KONG_22 | 38.645 | 75.031 | 4042 | 5 | 2.7 | 0 | 1 | 28.39±0.90 | KNSTD |  | 5.55±0.27 | Peak: 4.32 ka | 5.45±0.27 | 5.41±0.36 | 5.48±0.38 | 5.29±0.44 | 5.15±0.46 | 5.40±0.76 | 5.76±0.76 | 5.34±0.62 | 5.45±0.17 | | 5.50±0.18 | 5.06±0.16 |  |
| Seong et al., 2009 | Olimde 6 stage | m6H | KONG_23 | 38.644 | 75.028 | 4008 | 5 | 2.7 | 0 | 1 | 17.63±0.72 | KNSTD | 3.97±0.30 | 3.62±0.21 | Chi-squared: 2.17 | 3.58±0.24 | 3.44±0.28 | 3.50±0.33 | 3.45±0.29 | 3.30±0.30 | 3.48±0.64 | 3.84±0.64 | 3.42±0.46 | 3.31±0.14 | | 3.41±0.14 | 3.20±0.13 |  |
| Seong et al., 2009 | Olimde 6 stage | m6H | KONG_24 | 38.643 | 75.028 | 4005 | 5 | 2.7 | 0 | 1 | 9.13±0.63 | KNSTD |  | 1.85±0.16 | Skewness: -1.73 | 1.8±0.17 | 1.89±0.24 | 1.93±0.25 | 1.80±0.20 | 1.69±0.19 | 1.90±0.42 | 2.23±0.42 | 1.87±0.33 | 1.66±0.12 | | 1.73±0.12 | 1.66±0.12 |  |
| Seong et al., 2009 | Olimde 6 stage | m6H | KONG_25 | 38.645 | 75.026 | 3999 | 5 | 2.7 | 0 | 1 | 20.25±0.63 | KNSTD |  | 4.14±0.22 | Outlier: 3 | 4.1±0.25 | 4.00±0.40 | 4.07±0.45 | 3.94±0.31 | 3.76±0.33 | 4.02±0.75 | 4.48±0.75 | 3.94±0.59 | 3.93±0.12 | | 3.98±0.13 | 3.69±0.12 |  |
| Seong et al., 2009 | Olimde 6 stage | m6H | KONG_26 | 38.645 | 75.026 | 3997 | 5 | 2.7 | 0 | 1 | 20.25±0.72 | KNSTD |  | 4.15±0.24 | Wt. mean: 3.95±0.30 ka | 4.12±0.27 | 3.99±0.41 | 4.08±0.46 | 3.94±0.32 | 3.76±0.34 | 4.03±0.75 | 4.48±0.75 | 3.90±0.60 | 3.93±0.14 | | 3.98±0.14 | 3.70±0.13 |  |
| Seong et al., 2009 | Olimde 6 stage | m6H | KONG_27 | 38.646 | 75.026 | 4000 | 5 | 2.7 | 0 | 1 | 14.20±0.72 | KNSTD |  | 2.94±0.2 | Ar. Mean: 3.97±0.30 ka | 2.91±0.23 | 2.88±0.21 | 2.90±0.20 | 2.84±0.24 | 2.63±0.26 | 2.89±0.41 | 3.20±0.41 | 2.86±0.32 | 2.76±0.14 | | 2.81±0.14 | 2.59±0.13 |  |
| Seong et al., 2009 | Olimde 6 stage | m6H | KONG_28 | 38.646 | 75.026 | 4000 | 5 | 2.7 | 0 | 1 | 6.96±0.54 | KNSTD |  | 1.4±0.13 | Peak: 4.13 ka | 1.35±0.13 | 1.44±0.15 | 1.47±0.17 | 1.42±0.14 | 1.29±0.15 | 1.46±0.34 | 1.62±0.34 | 1.44±0.21 | 1.28±0.10 | | 1.33±0.10 | 1.27±0.10 |  |
| Hedrick et al., 2011 | PM-2 stage | N/A | India-10 | 33.245 | 78.2 | 4910 | 3 | 2.7 | 0 | 1 | 62.54±3.21 | 07KNSTD | 3.50±0.87 | 8.94±0.67 | Chi-squared: 17.79 | 9.05±0.77 | 9.32±0.92 | 9.50±0.99 | 8.51±0.85 | 8.50±0.82 | 8.70±1.50 | 9.30±1.50 | 8.60±1.30 | 9.45±0.49 | | 9.31±0.48 | 8.36±0.43 |  |
| Hedrick et al., 2011 | PM-2 stage | N/A | India-11 | 33.245 | 78.201 | 4905 | 3 | 2.7 | 0 | 1 | 42.94±2.96 | 07KNSTD |  | 6.3±0.45 | Skewness: +1.18 | 6.44±0.46 | 6.41±0.73 | 6.54±0.77 | 5.99±0.58 | 5.85±0.62 | 6.00±1.30 | 6.60±1.30 | 5.91±0.88 | 6.59±0.46 | | 6.48±0.45 | 5.75±0.40 |  |
| Hedrick et al., 2011 | PM-2 stage | N/A | India-12 | 33.245 | 78.201 | 4904 | 2 | 2.7 | 0 | 1 | 20.84±1.97 | 07KNSTD |  | 3.25±0.33 | Outlier: 4 | 3.4±0.36 | 3.17±0.37 | 3.23±0.37 | 3.10±0.34 | 2.81±0.35 | 3.00±0.52 | 3.32±0.52 | 2.99±0.34 | 3.15±0.30 | | 3.14±0.30 | 2.77±0.26 |  |
| Hedrick et al., 2011 | PM-2 stage | N/A | India-13 | 33.245 | 78.201 | 4899 | 2 | 2.7 | 0 | 1 | 307.50±7.55 | 07KNSTD |  | 39.27±1.69 | Wt. mean: 3.42±0.87 ka | 37.82±2.04 | 36.50±2.40 | 37.20±2.60 | 37.70±2.70 | 42.00±3.60 | 36.30±4.40 | 35.50±4.40 | 35.50±4.10 | 36.25±0.90 | | 38.26±0.95 | 41.32±1.03 |  |
| Hedrick et al., 2011 | PM-2 stage | N/A | India-14 | 33.244 | 78.202 | 4886 | 2 | 2.7 | 0 | 1 | 5.45±1.22 | 07KNSTD |  | 0.81±0.19 | Ar. Mean: 3.50±0.87 ka | 0.84±0.2 | 1.01±0.14 | 1.02±0.16 | 0.92±0.17 | 0.74±0.18 | 0.95±0.25 | 0.97±0.25 | 0.95±0.19 | 0.91±0.21 | | 0.89±0.20 | 0.73±0.16 |  |
| Hedrick et al., 2011 | PM-2 stage | N/A | India-56 | 33.244 | 78.198 | 4924 | 3 | 2.7 | 0 | 1 | 29.04±0.75 | 07KNSTD |  | 4.46±0.22 | Peak: 2.80 ka | 4.65±0.26 | 4.50±0.40 | 4.58±0.43 | 4.17±0.31 | 3.92±0.33 | 4.16±0.74 | 4.65±0.74 | 4.11±0.65 | 4.42±0.11 | | 4.35±0.11 | 3.85±0.10 |  |
| Hedrick et al., 2011 | PM-2 stage | N/A | India-57 | 33.245 | 78.199 | 4921 | 3 | 2.7 | 0 | 1 | 17.76±0.70 | 07KNSTD |  | 2.78±0.18 |  | 2.91±0.21 | 2.82±0.15 | 2.85±0.17 | 2.70±0.22 | 2.40±0.22 | 2.68±0.28 | 2.93±0.28 | 2.70±0.30 | 2.75±0.11 | | 2.74±0.11 | 2.36±0.09 |  |
| Dortch et al., 2013 | Ladakh Chang La cirque | N/A | Chang-1 | 34.04 | 77.93 | 5461 | 5 | 2.7 | 0 | 1 | 17.00±2.00 | 07KNSTD | 2.29±0.28 | 2.02±0.27 | Chi-squared: 2.02 | 2.07±0.29 | 2.16±0.35 | 2.21±0.34 | 1.99±0.29 | 1.79±0.26 | 1.97±0.45 | 2.26±0.45 | 1.95±0.39 | 1.88±0.22 | | 1.92±0.23 | 1.77±0.21 |  |
| Dortch et al., 2013 | Ladakh Chang La cirque | N/A | Chang-2 | 34.04 | 77.93 | 5459 | 3 | 2.7 | 0 | 1 | 20.00±2.00 | 07KNSTD |  | 2.36±0.28 | Skewness: +0.72 | 2.44±0.31 | 2.51±0.26 | 2.55±0.26 | 2.34±0.31 | 2.08±0.27 | 2.31±0.39 | 2.59±0.39 | 2.30±0.40 | 2.29±0.23 | | 2.34±0.23 | 2.05±0.21 |  |
| Dortch et al., 2013 | Ladakh Chang La cirque | N/A | Chang-3 | 34.04 | 77.93 | 5458 | 4 | 2.7 | 0 | 1 | 22.00±1.00 | 07KNSTD |  | 2.65±0.18 | Outlier: 2 | 2.73±0.21 | 2.71±0.16 | 2.75±0.18 | 2.59±0.22 | 2.30±0.21 | 2.53±0.33 | 2.80±0.33 | 2.51±0.32 | 2.62±0.12 | | 2.64±0.12 | 2.27±0.10 |  |
| Dortch et al., 2013 | Ladakh Chang La cirque | N/A | Chang-4 | 34.04 | 77.93 | 5460 | 5 | 2.7 | 0 | 1 | 55.00±2.00 | 07KNSTD |  | 6.27±0.31 | Wt. mean: 2.31±0.28 ka | 6.31±0.33 | 6.20±0.60 | 6.39±0.67 | 5.96±0.48 | 5.81±0.52 | 5.77±1.20 | 6.20±1.20 | 5.68±0.67 | 6.48±0.24 | | 6.44±0.24 | 5.72±0.21 |  |
| Dortch et al., 2013 | Ladakh Chang La cirque | N/A | Chang-5 | 34.04 | 77.93 | 5462 | 4 | 2.7 | 0 | 1 | 18.00±1.00 | 07KNSTD |  | 2.13±0.16 | Ar. Mean: 2.29±0.28 ka | 2.18±0.19 | 2.28±0.23 | 2.33±0.22 | 2.10±0.22 | 1.88±0.19 | 2.08±0.36 | 2.37±0.36 | 2.05±0.34 | 1.99±0.11 | | 2.02±0.11 | 1.85±0.10 |  |
| Dortch et al., 2013 | Ladakh Chang La cirque | N/A | Chang-6 | 34.04 | 77.93 | 5445 | 5 | 2.7 | 0 | 1 | 97.00±3.00 | 07KNSTD |  | 10.8±0.51 | Peak: 2.15 ka | 10.7±0.54 | 11.19±0.92 | 11.00±1.00 | 10.48±0.93 | 10.30±0.90 | 10.30±1.60 | 10.90±1.60 | 10.10±1.50 | 10.93±0.34 | | 11.03±0.34 | 10.17±0.32 |  |
| Saha et al., 2018 | Late Holocene | mG1 | KO-14 | 32.9672 | 78.1794 | 5374 | 3 | 2.7 | 0 | 0.961 | 17.10±0.72 | 07KNSTD | 2.25±0.42 | 2.36±0.19 | Chi-squared: 9.37 | 2.27±0.19 | 2.43±0.19 | 2.47±0.21 | 2.24±0.22 | 1.98±0.18 | 2.22±0.37 | 2.49±0.37 | 2.21±0.33 | 1.95±0.08 | | 2.20±0.09 | 2.20±0.09 |  |
|  |  |  |  |  |  |  |  |  |  |  |  |  |  |  | Skewness: -1.07 |  |  |  |  |  |  |  |  |  |  |  |  |  |
| Saha et al., 2018 | Late Holocene | mG1 | KO-15 | 32.9678 | 78.1796 | 5366 | 3 | 2.7 | 0 | 0.966 | 4.93±0.26 | 07KNSTD |  | 0.64±0.05 | Outlier: 2 | 0.62±0.05 | 0.82±0.06 | 0.83±0.08 | 0.73±0.06 | 0.57±0.06 | 0.74±0.12 | 0.72±0.12 | 0.74±0.10 | 0.56±0.03 | | 0.72±0.04 | 0.73±0.04 |  |
| Saha et al., 2018 | Late Holocene | mG1 | KO-16 | 32.9678 | 78.1796 | 5366 | 4 | 2.7 | 0 | 0.966 | 3.87±0.32 | 07KNSTD |  | 0.5±0.06 | Wt. mean: 2.10±0.42 ka | 0.48±0.06 | 0.66±0.07 | 0.67±0.09 | 0.58±0.06 | 0.45±0.05 | 0.59±0.10 | 0.57±0.10 | 0.59±0.09 | 0.44±0.04 | | 0.57±0.05 | 0.58±0.05 |  |
| Saha et al., 2018 | Late Holocene | mG1 | KO1601 | 32.968 | 78.1796 | 5374 | 2 | 2.7 | 0 | 0.966 | 13.40±0.39 | 07KNSTD |  | 1.79±0.12 | Ar. Mean: 2.25±0.42 ka | 1.73±0.12 | 1.86±0.17 | 1.90±0.19 | 1.71±0.14 | 1.53±0.13 | 1.69±0.32 | 1.92±0.32 | 1.68±0.24 | 1.51±0.04 | | 1.65±0.05 | 1.63±0.05 |  |
| Saha et al., 2018 | Late Holocene | mG1 | KO1602 | 32.9671 | 78.18 | 5388 | 1.5 | 2.7 | 0 | 0.954 | 18.94±0.71 | 07KNSTD |  | 2.61±0.19 | Peak: 2.48 ka ! | 2.51±0.19 | 2.63±0.16 | 2.67±0.18 | 2.47±0.22 | 2.17±0.19 | 2.42±0.36 | 2.69±0.36 | 2.41±0.31 | 2.14±0.08 | | 2.53±0.10 | 2.51±0.09 |  |
| Seong et al., 2009 | Olimde 7 stage | m7A | MUST-32 | 38.361 | 75.171 | 4082 | 5 | 2.7 | 0 | 0.99 | 10.76±0.54 | KNSTD | 2.20±0.07 | 2.15±0.16 | Chi-squared: 0.19 | 2.1±0.17 | 2.24±0.23 | 2.28±0.26 | 2.11±0.21 | 1.94±0.19 | 2.22±0.34 | 2.54±0.34 | 2.19±0.35 | 1.93±0.10 | | 2.00±0.10 | 1.91±0.10 |  |
| Seong et al., 2009 | Olimde 7 stage | m7A | MUST-33 | 38.361 | 75.171 | 4081 | 5 | 2.7 | 0 | 0.99 | 11.21±0.54 | KNSTD |  | 2.24±0.16 | Skewness: +1.07 | 2.2±0.18 | 2.34±0.22 | 2.38±0.24 | 2.21±0.22 | 2.00±0.20 | 2.32±0.34 | 2.63±0.34 | 2.29±0.35 | 2.03±0.10 | | 2.10±0.10 | 1.99±0.10 |  |
| Seong et al., 2009 | Olimde 7 stage | m7A | MUST-34 | 38.36 | 75.172 | 4094 | 5 | 2.7 | 0 | 0.99 | 11.66±0.54 | KNSTD |  | 2.32±0.17 | Outlier: 1 | 2.28±0.19 | 2.40±0.20 | 2.45±0.22 | 2.29±0.23 | 2.10±0.20 | 2.39±0.35 | 2.70±0.35 | 2.36±0.33 | 2.11±0.10 | | 2.19±0.10 | 2.06±0.10 |  |
| Seong et al., 2009 | Olimde 7 stage | m7A | MUST-35 | 38.359 | 75.172 | 4120 | 5 | 2.7 | 0 | 0.98 | 13.56±0.54 | KNSTD |  | 2.71±0.17 | Wt. mean: 2.19±0.07 ka | 2.69±0.19 | 2.72±0.17 | 2.76±0.18 | 2.64±0.22 | 2.43±0.22 | 2.71±0.32 | 3.00±0.32 | 2.68±0.32 | 2.58±0.10 | | 2.62±0.11 | 2.39±0.10 |  |
| Seong et al., 2009 | Olimde 7 stage | m7A | MUST-36 | 38.356 | 75.173 | 4167 | 5 | 2.7 | 0 | 0.99 | 11.48±0.54 | KNSTD |  | 2.19±0.15 | Ar. Mean: 2.20±0.07 ka | 2.15±0.18 | 2.29±0.24 | 2.32±0.22 | 2.16±0.22 | 1.99±0.19 | 2.26±0.34 | 2.58±0.34 | 2.23±0.33 | 1.97±0.09 | | 2.05±0.10 | 1.95±0.09 |  |
| Seong et al., 2009 | Olimde 7 stage | m7A | MUST-37 | 38.356 | 75.173 | 4176 | 5 | 2.7 | 0 | 0.99 | 11.30±0.45 | KNSTD |  | 2.15±0.14 | Peak: 2.19 ka | 2.1±0.16 | 2.23±0.22 | 2.28±0.24 | 2.10±0.20 | 1.95±0.18 | 2.21±0.33 | 2.54±0.33 | 2.18±0.35 | 1.92±0.08 | | 2.00±0.08 | 1.91±0.08 |  |
| Seong et al., 2009 | Olimde 7 stage | m7A | MUST-38 | 38.355 | 75.173 | 4180 | 5 | 2.7 | 0 | 0.99 | 11.21±0.81 | KNSTD |  | 2.12±0.19 |  | 2.08±0.21 | 2.21±0.26 | 2.25±0.28 | 2.09±0.25 | 1.93±0.22 | 2.19±0.39 | 2.51±0.39 | 2.15±0.38 | 1.90±0.14 | | 1.98±0.14 | 1.89±0.14 |  |
| Seong et al., 2009 | Olimde 7 stage | m7H | KONG_14 | 38.644 | 75.041 | 4168 | 5 | 2.7 | 0 | 1 | 9.58±0.99 | KNSTD | 1.66±0.17 | 1.78±0.21 | Chi-squared: 0.75 | 1.72±0.22 | 1.80±0.26 | 1.80±0.30 | 1.76±0.24 | 1.63±0.23 | 1.81±0.49 | 2.12±0.49 | 1.78±0.34 | 1.58±0.16 | | 1.67±0.17 | 1.60±0.17 |  |
|  |  |  |  |  |  |  |  |  |  |  |  |  |  |  | Skewness: -1.63 ka |  |  |  |  |  |  |  |  |  |  |  |  |  |
|  |  |  |  |  |  |  |  |  |  |  |  |  |  |  | Outlier: 1 |  |  |  |  |  |  |  |  |  |  |  |  |  |
| Seong et al., 2009 | Olimde 7 stage | m7H | KONG_15 | 38.644 | 75.04 | 4178 | 5 | 2.7 | 0 | 1 | 5.15±0.90 | KNSTD |  | 0.94±0.17 | Wt. mean: 1.66±0.17 ka | 0.89±0.17 | 1.05±0.14 | 1.06±0.16 | 1.02±0.17 | 0.87±0.18 | 1.04±0.21 | 1.08±0.21 | 1.03±0.18 | 0.97±0.17 | | 0.97±0.17 | 0.86±0.15 |  |
| Seong et al., 2009 | Olimde 7 stage | m7H | KONG_16 | 38.644 | 75.04 | 4179 | 5 | 2.7 | 0 | 1 | 9.40±0.90 | KNSTD |  | 1.74±0.19 | Ar. Mean: 1.66±0.17 ka | 1.68±0.2 | 1.75±0.26 | 1.79±0.26 | 1.72±0.22 | 1.59±0.21 | 1.76±0.45 | 2.06±0.45 | 1.73±0.32 | 1.54±0.15 | | 1.63±0.16 | 1.56±0.15 |  |
| Seong et al., 2009 | Olimde 7 stage | m7H | KONG_17 | 38.643 | 75.039 | 4178 | 5 | 2.7 | 0 | 1 | 7.96±0.99 | KNSTD |  | 1.46±0.2 | Peak: 1.70 ka | 1.4±0.2 | 1.49±0.21 | 1.52±0.21 | 1.47±0.21 | 1.35±0.22 | 1.50±0.41 | 1.68±0.41 | 1.48±0.27 | 1.31±0.16 | | 1.38±0.17 | 1.32±0.17 |  |
| Orr et al., 2017 | Late Holocene | mS2 | STOK-1401 | 33.9873 | 77.4557 | 5329 | 3 | 2.7 | 0 | 0.964 | 15.57±0.51 | 07KNSTD | 1.42±0.48 | 2.06±0.14 | Chi-squared: 21.83 | 2.00±0.14 | 2.16±0.21 | 2.21±0.23 | 1.98±0.18 | 1.78±0.16 | 1.97±0.37 | 2.26±0.37 | 1.90±0.30 | 1.75±0.06 | | 1.90±0.06 | 1.87±0.06 |  |
| Orr et al., 2017 | Late Holocene | mS2 | STOK-1402 | 33.9879 | 77.4557 | 5330 | 3 | 2.7 | 0 | 0.967 | 11.72±0.58 | 07KNSTD |  | 1.51±0.12 | Skewness: +0.34 | 1.48±0.12 | 1.59±0.14 | 1.61±0.13 | 1.50±0.12 | 1.33±0.13 | 1.48±0.30 | 1.60±0.30 | 1.50±0.20 | 1.31±0.07 | | 1.43±0.07 | 1.38±0.07 |  |
| Orr et al., 2017 | Late Holocene | mS2 | STOK-1403 | 33.9883 | 77.457 | 5323 | 1 | 2.7 | 0 | 0.969 | 14.65±0.65 | 07KNSTD |  | 1.89±0.14 | Outlier: 3 | 1.84±0.14 | 2.00±0.20 | 2.00±0.20 | 1.82±0.16 | 1.64±0.15 | 1.81±0.37 | 2.08±0.37 | 1.79±0.28 | 1.62±0.07 | | 1.75±0.08 | 1.72±0.08 |  |
| Orr et al., 2017 | Late Holocene | mS2 | STOK-1404 | 33.9882 | 77.4571 | 5328 | 2 | 2.7 | 0 | 0.969 | 2.08±0.09 | 07KNSTD |  | 0.25±0.02 | Wt. mean: 1.20±0.48 ka | 0.25±0.02 | 0.34±0.04 | 0.35±0.04 | 0.32±0.03 | 0.24±0.02 | 0.30±0.05 | 0.29±0.05 | 0.30±0.04 | 0.23±0.01 | | 0.32±0.01 | ± |  |
| Orr et al., 2017 | Late Holocene | mS1 | STOK-1405 | 33.9872 | 77.4601 | 5370 | 2 | 2.7 | 0 | 0.98 | 7.64±0.31 | 07KNSTD |  | 0.93±0.07 | Ar. Mean: 1.42±0.48 ka | 0.92±0.07 | 1.08±0.07 | 1.10±0.07 | 1.02±0.07 | 0.84±0.08 | 1.02±0.13 | 1.05±0.13 | 1.00±0.10 | 0.82±0.03 | | 0.98±0.04 | 0.98±0.04 |  |
| Orr et al., 2017 | Late Holocene | mS1 | STOK-1406 | 33.987 | 77.46 | 5314 | 2.5 | 2.7 | 0 | 0.98 | 4.54±0.22 | 07KNSTD |  | 0.56±0.04 | Peak: ! | 0.55±0.04 | 0.73±0.06 | 0.74±0.07 | 0.65±0.06 | 0.51±0.05 | 0.66±0.11 | 0.64±0.11 | 0.66±0.10 | 0.50±0.02 | | 0.63±0.03 | 0.65±0.03 |  |
| Orr et al., 2017 | Late Holocene | mS1 | STOK-1407 | 33.9871 | 77.4608 | 5340 | 3 | 2.7 | 0 | 0.978 | 1.25±0.21 | 07KNSTD |  | 0.14±0.03 |  | 0.14±0.03 | 0.20±0.03 | 0.21±0.03 | 0.19±0.04 | 0.14±0.03 | 0.18±0.04 | 0.17±0.04 | 0.18±0.04 | 0.14±0.02 | | 0.19±0.03 | ± |  |
| Orr et al., 2017 | Late Holocene | mS1 | STOK-1408 | 33.9871 | 77.4606 | 5308 | 3 | 2.7 | 0 | 0.978 | 9.31±0.72 | 07KNSTD |  | 1.19±0.11 |  | 1.17±0.11 | 1.29±0.13 | 1.31±0.11 | 1.23±0.11 | 1.06±0.12 | 1.21±0.24 | 1.28±0.24 | 1.21±0.14 | 1.04±0.08 | | 1.16±0.09 | 1.14±0.09 |  |
| Saha et al., 2018 | Late Holocene | mS1 | STOK-1516 | 33.9872 | 77.4599 | 5303 | 3 | 2.7 | 0 | 0.977 | 7.37±0.65 | 07KNSTD |  | 0.94±0.1 |  | 0.92±0.1 | 1.09±0.09 | 1.10±0.09 | 1.02±0.09 | 0.84±0.10 | 1.02±0.15 | 1.05±0.15 | 1.02±0.11 | 0.83±0.07 | | 0.98±0.09 | 0.99±0.09 |  |
| Seong et al., 2009 | Olimde 7 stage | m3I | KONG_35 | 38.709 | 75.28 | 3326 | 5 | 2.7 | 0 | 0.93 | 3.07±0.54 | KNSTD | 1.39±0.42 | 0.95±0.18 | Chi-squared: 4.58 | 0.94±0.18 | 1.08±0.17 | 1.09±0.18 | 1.02±0.17 | 0.88±0.19 | 1.09±0.24 | 1.13±0.24 | 1.08±0.19 | 0.99±0.18 | | 0.97±0.17 | 0.87±0.15 |  |
|  |  |  |  |  |  |  |  |  |  |  |  |  |  |  | Skewness: -0.03 |  |  |  |  |  |  |  |  |  |  |  |  |  |
| Seong et al., 2009 | Olimde 7 stage of | m3I | KONG_36 | 38.709 | 75.28 | 3328 | 5 | 2.7 | 0 | 0.93 | 5.33±0.63 | KNSTD |  | 1.67±0.22 | Outlier: 1 | 1.66±0.23 | 1.74±0.29 | 1.76±0.32 | 1.65±0.25 | 1.53±0.24 | 1.79±0.48 | 2.06±0.48 | 1.77±0.36 | 1.53±0.18 | | 1.56±0.19 | 1.50±0.18 |  |
| Seong et al., 2009 | Olimde 7 stage of | m3I | KONG_37 | 38.711 | 75.28 | 3309 | 5 | 2.7 | 0 | 0.93 | 5.70±0.54 | KNSTD |  | 1.81±0.2 | Wt. mean: 1.35±0.42 ka | 1.8±0.21 | 1.90±0.26 | 1.92±0.29 | 1.78±0.23 | 1.65±0.22 | 1.90±0.43 | 2.25±0.43 | 1.92±0.36 | 1.67±0.16 | | 1.69±0.16 | 1.62±0.15 |  |
| Seong et al., 2009 | Olimde 7 stage of | m3I | KONG_38 | 38.714 | 75.28 | 3299 | 5 | 2.7 | 0 | 0.93 | 3.53±0.63 | KNSTD |  | 1.11±0.21 | Ar. Mean: 1.39±0.42 ka | 1.09±0.21 | 1.21±0.21 | 1.22±0.23 | 1.16±0.22 | 1.03±0.22 | 1.23±0.37 | 1.31±0.37 | 1.22±0.26 | 1.10±0.20 | | 1.09±0.20 | 1.01±0.18 |  |
| Seong et al., 2009 | Olimde 7 stage of | m3I | KONG_41 | 38.738 | 75.273 | 3118 | 5 | 2.7 | 0 | 0.93 | 1.27±0.45 | KNSTD |  | 0.43±0.16 | Peak: 1.02 ka ! | 0.43±0.16 | 0.56±0.20 | 0.56±0.22 | 0.50±0.18 | 0.41±0.17 | 0.55±0.22 | 0.54±0.22 | 0.55±0.21 | 0.00±0.00 | | 0.00±0.00 | 0.00±0.00 |  |
| Orr et al., 2017 | N/A | mG1 | ZK34 | 34.0043 | 77.4618 | 5280 | 3 | 2.7 | 0 | 0.96 | 16.20±1.20 | 07KNSTD | 1.33±0.12 | 2.22±0.18 | ! | 2.14±0.20 | 2.31±0.24 | 2.45±0.24 | 1.94±0.21 | 2.16±0.26 | 1.98±0.24 | 2.28±0.25 | 2.18±0.25 | 2.01±0.15 | | 2.04±0.15 | 1.87±0.14 |  |
| Orr et al., 2017 | N/A | mG1 | ZK36 | 34.0042 | 77.461 | 5294 | 3 | 2.7 | 0 | 1 | 10.50±0.70 | 07KNSTD |  | 1.33±0.12 |  | 1.29±0.11 | 1.41±0.12 | 1.5±0.15 | 1.20±0.12 | 1.36±0.12 | 1.26±0.11 | 1.35±0.15 | 1.36±0.12 | 1.23±0.08 | | 1.26±0.08 | 1.16±0.07 |  |
| Seong et al., 2007 | Askole 3 stage | m1H | K2-90 | 35.688 | 75.927 | 3095 | 3 | 2.7 | 0 | 0.97 | 1.81±0.90 | KNSTD | 1.03±0.28 | 0.70±0.34 | Chi-squared: 0.82 | 0.66±0.32 | 0.84±0.07 | 0.84±0.07 | 0.74±0.05 | 0.60±0.05 | 0.83±0.14 | 0.83±0.14 | 0.83±0.11 | 0.00±0.00 | | 0.00±0.00 | 0.00±0.00 |  |
| Seong et al., 2007 | Askole 3 stage | m1H | K2-91 | 35.695 | 75.927 | 3096 | 3 | 2.7 | 0 | 0.96 | 3.62±0.90 | KNSTD |  | 1.34±0.35 | Skewness: -0.08 | 1.41±0.38 | 1.49±0.39 | 1.50±0.34 | 1.36±0.34 | 1.22±0.35 | 1.50±0.60 | 1.70±0.60 | 1.50±0.43 | 1.30±0.33 | | 1.28±0.32 | 1.20±0.30 |  |
| Seong et al., 2007 | Askole 3 stage | m1H | K2-92 | 35.687 | 75.926 | 3094 | 3 | 2.7 | 0 | 0.96 | 2.71±0.90 | KNSTD |  | 1.05±0.36 | Outlier: 2 | 1.00±0.34 | 1.16±0.07 | 1.16±0.08 | 1.07±0.07 | 0.92±0.07 | 1.16±0.18 | 1.22±0.18 | 1.16±0.13 | 0.00±0.00 | | 0.00±0.00 | 0.00±0.00 |  |
| Seong et al., 2007 | Askole 3 stage | m1H | K2-93 | 35.686 | 75.926 | 3090 | 2 | 2.7 | 0 | 0.96 | 7.23±0.90 | KNSTD |  | 2.75±0.37 | Wt. mean: 1.03±0.28 ka | 2.94±0.41 | 2.88±0.33 | 2.90±0.31 | 2.70±0.40 | 2.43±0.39 | 2.90±0.54 | 3.16±0.54 | 2.88±0.41 | 2.77±0.35 | | 2.66±0.33 | 2.38±0.30 |  |
| Seong et al., 2007 | Askole 3 stage | m1H | K2-94 | 35.688 | 75.925 | 3096 | 3 | 2.7 | 0 | 0.96 | 3.62±0.90 | KNSTD |  | 1.34±0.35 | Ar. Mean: 1.03±0.28 ka | 1.41±0.38 | 1.49±0.39 | 1.50±0.34 | 1.36±0.34 | 1.22±0.35 | 1.50±0.60 | 1.70±0.60 | 1.50±0.43 | 1.30±0.33 | | 1.28±0.32 | 1.20±0.30 |  |
| Seong et al., 2007 | Askole 3 stage | m1H | K2-95 | 35.292 | 75.662 | 3087 | 3 | 2.7 | 0 | 0.96 | 1.81±0.90 | KNSTD |  | 0.72±0.35 | Peak: 1.02 ka | 0.68±0.33 | 0.86±0.07 | 0.87±0.08 | 0.76±0.05 | 0.62±0.05 | 0.85±0.14 | 0.86±0.14 | 0.86±0.12 | 0.00±0.00 | | 0.00±0.00 | 0.00±0.00 |  |
| Seong et al., 2007 | Askole 3 stage | m1H | K2-96 | 35.292 | 75.662 | 3116 | 3 | 2.7 | 0 | 0.97 | 4.52±0.90 | KNSTD |  | 1.67±0.35 |  | 1.78±0.39 | 1.85±0.49 | 1.87±0.52 | 1.65±0.38 | 1.51±0.36 | 1.86±0.62 | 2.11±0.62 | 1.85±0.52 | 1.61±0.32 | | 1.56±0.31 | 1.48±0.30 |  |
| Saha et al., 2018 | Late Holocene | mM2 | KO1 | 32.9321 | 78.2143 | 5532 | 3 | 2.7 | 0 | 1 | 20.44±0.66 | 07KNSTD | 1.00±0.08 | 2.56±0.19 | Chi-squared: 0.90 | 2.46±0.19 | 2.59±0.18 | 2.63±0.17 | 2.42±0.21 | 2.13±0.19 | 2.37±0.33 | 2.63±0.33 | 2.30±0.30 | 2.10±0.07 | | 2.48±0.08 | 2.44±0.08 |  |
|  |  |  |  |  |  |  |  |  |  |  |  |  |  |  | Skewness: -0.17 |  |  |  |  |  |  |  |  |  |  |  |  |  |
| Saha et al., 2018 | Late Holocene | mM2 | KO-2 | 32.9317 | 78.2143 | 5541 | 6 | 2.7 | 0 | 1 | 9.03±0.53 | 07KNSTD |  | 1.09±0.09 | Outlier: 1 | 1.07±0.09 | 1.21±0.08 | 1.23±0.11 | 1.14±0.09 | 0.96±0.10 | 1.12±0.16 | 1.16±0.16 | 1.12±0.12 | 0.95±0.06 | | 1.09±0.07 | 1.08±0.06 |  |
| Saha et al., 2018 | Late Holocene | mM2 | KO-3 | 32.9314 | 78.2142 | 5548 | 4 | 2.7 | 0 | 0.991 | 8.18±0.39 | 07KNSTD |  | 0.98±0.07 | Wt. mean: 0.99±0.08 ka | 0.95±0.07 | 1.12±0.06 | 1.13±0.08 | 1.05±0.08 | 0.86±0.08 | 1.04±0.13 | 1.06±0.13 | 1.00±0.10 | 0.85±0.04 | | 1.01±0.05 | 1.01±0.05 |  |
| Saha et al., 2018 | Late Holocene | mM2 | MENTOK-1505 | 32.9312 | 78.2145 | 5569 | 2 | 2.7 | 0 | 0.991 | 8.76±0.32 | 07KNSTD |  | 1.02±0.07 | Ar. Mean: 1.00±0.08 ka | 1.00±0.07 | 1.15±0.07 | 1.17±0.08 | 1.08±0.08 | 0.90±0.08 | 1.07±0.13 | 1.10±0.13 | 1.07±0.11 | 0.89±0.03 | | 1.04±0.04 | 1.04±0.04 |  |
| Saha et al., 2018 | Late Holocene | mM2 | MENTOK-1506 | 32.9311 | 78.2145 | 5574 | 1 | 2.7 | 0 | 0.992 | 7.84±0.50 | 07KNSTD |  | 0.90±0.08 | Peak: 1.00 ka | 0.88±0.08 | 1.06±0.07 | 1.08±0.08 | 0.99±0.09 | 0.80±0.08 | 0.98±0.15 | 1.00±0.15 | 0.99±0.11 | 0.78±0.05 | | 0.95±0.06 | 0.96±0.06 |  |
| Seong et al., 2009 | Olimde 8 stage | m8A | MUST-39 | 38.353 | 75.171 | 4228 | 5 | 2.7 | 0 | 1 | 2.62±0.45 | KNSTD | 0.69±0.27 | 0.46±0.08 | Chi-squared: 10.19 | 0.44±0.08 | 0.58±0.12 | 0.59±0.12 | 0.54±0.10 | 0.44±0.09 | 0.56±0.12 | 0.54±0.12 | 0.55±0.12 | 0.53±0.09 | | 0.52±0.09 | 0.43±0.07 |  |
|  |  |  |  |  |  |  |  |  |  |  |  |  |  |  | Skewness: +1.08 |  |  |  |  |  |  |  |  |  |  |  |  |  |
|  |  |  |  |  |  |  |  |  |  |  |  |  |  |  | Outlier: 0 |  |  |  |  |  |  |  |  |  |  |  |  |  |
|  |  |  |  |  |  |  |  |  |  |  |  |  |  |  | Wt. mean: 0.66±0.27 ka |  |  |  |  |  |  |  |  |  |  |  |  |  |
| Seong et al., 2009 | Olimde 8 stage | m8A | MUST-40 | 38.353 | 75.171 | 4227 | 5 | 2.7 | 0 | 1 | 5.52±0.45 | KNSTD |  | 0.99±0.09 | Ar. Mean: 0.69±0.27 ka | 0.95±0.1 | 1.09±0.10 | 1.10±0.08 | 1.06±0.11 | 0.92±0.11 | 1.08±0.17 | 1.12±0.17 | 1.07±0.13 | 1.01±0.08 | | 1.01±0.08 | 0.90±0.07 |  |
| Seong et al., 2009 | Olimde 8 stage | m8A | MUST-41 | 38.353 | 75.17 | 4238 | 5 | 2.7 | 0 | 1 | 3.53±0.36 | KNSTD |  | 0.62±0.07 | Peak: 0.60 ka ! | 0.6±0.07 | 0.76±0.10 | 0.77±0.11 | 0.71±0.09 | 0.58±0.08 | 0.73±0.15 | 0.72±0.15 | 0.73±0.11 | 0.70±0.07 | | 0.68±0.07 | 0.57±0.06 |  |
| Saha et al., 2018 | Late Holocene | mM1 | KO-7 | 32.9328 | 78.2136 | 5524 | 6 | 2.7 | 0 | 1 | 2.04±0.14 | 07KNSTD | 0.64±0.09 | 0.24±0.02 | Chi-squared: 1.47 | 0.23±0.02 | 0.32±0.03 | 0.33±0.04 | 0.30±0.03 | 0.22±0.02 | 0.28±0.05 | 0.27±0.05 | 0.28±0.05 | 0.22±0.01 | | 0.30±0.02 | ± |  |
|  |  |  |  |  |  |  |  |  |  |  |  |  |  |  | Skewness: -1.71 |  |  |  |  |  |  |  |  |  |  |  |  |  |
|  |  |  |  |  |  |  |  |  |  |  |  |  |  |  | Outlier: 1 |  |  |  |  |  |  |  |  |  |  |  |  |  |
| Saha et al., 2018 | Late Holocene | mM1 | KO-8 | 32.9333 | 78.2136 | 5516 | 2.5 | 2.7 | 0 | 1 | 5.92±0.55 | 07KNSTD |  | 0.69±0.08 | Wt. mean: 0.67±0.09 ka | 0.68±0.08 | 0.88±0.11 | 0.90±0.10 | 0.79±0.09 | 0.62±0.08 | 0.79±0.15 | 0.78±0.15 | 0.80±0.11 | 0.61±0.06 | | 0.77±0.07 | 0.78±0.07 |  |
| Saha et al., 2018 | Late Holocene | mM1 | KO-9 | 32.9341 | 78.2147 | 5503 | 1 | 2.7 | 0 | 1 | 6.00±0.24 | 07KNSTD |  | 0.70±0.05 | Ar. Mean: 0.64±0.09 ka | 0.68±0.05 | 0.89±0.08 | 0.91±0.07 | 0.79±0.06 | 0.62±0.06 | 0.80±0.13 | 0.79±0.13 | 0.80±0.10 | 0.61±0.02 | | 0.78±0.03 | 0.79±0.03 |  |
| Saha et al., 2018 | Late Holocene | mM1 | KO10 | 32.9345 | 78.2148 | 5503 | 1 | 2.7 | 0 | 1 | 4.73±0.67 | 07KNSTD |  | 0.54±0.09 | Peak: 0.69 ka | 0.53±0.09 | 0.72±0.10 | 0.73±0.11 | 0.63±0.10 | 0.49±0.08 | 0.64±0.13 | 0.62±0.13 | 0.64±0.13 | 0.48±0.07 | | 0.62±0.09 | 0.63±0.09 |  |
| Blomdin et al. (2016) | Bordoo Valley | BOR 1 | TS-C-12-027 | 41.8125 | 78.1319 | 3775 | 2 | 2.7 | 0 | 0.99 | 2.52±0.37 | 07KNSTD | 0.64±0.23 | 0.45±0.07 | Chi-squared: 10.96 | 0.50±0.08 | 0.60±0.10 | 0.61±0.07 | 0.48±0.08 | 0.58±0.09 | 0.60±0.12 | 0.56±0.11 | 0.59±0.11 | 0.56±0.08 | | 0.55±0.08 | 0.47±0.07 |  |
|  |  |  |  |  |  |  |  |  |  |  |  |  |  |  | Skewness: +1.23 |  |  |  |  |  |  |  |  |  |  |  |  |  |
| Blomdin et al. (2016) | Bordoo Valley | BOR 1 | TS-C-12-028 | 41.8124 | 78.1314 | 3776 | 2 | 2.7 | 0 | 0.99 | 28.96±1.26 | 07KNSTD |  | 5.40±0.32 | Outlier: 0 | 5.78±0.33 | 5.50±0.40 | 5.54±0.39 | 5.51±0.51 | 5.61±0.48 | 5.69±0.84 | 6.00±1.00 | 5.55±0.66 | 5.54±0.24 | | 5.76±0.25 | 5.41±0.24 |  |
| Blomdin et al. (2016) | Bordoo Valley | BOR 1 | TS-C-12-029 | 41.8121 | 78.1299 | 3759 | 2.5 | 2.7 | 0 | 0.99 | 17.23±1.01 | 07KNSTD |  | 3.34±0.26 | Wt. mean: 0.65±0.23 ka | 3.61±0.26 | 3.34±0.28 | 3.40±0.32 | 3.32±0.33 | 3.48±0.32 | 3.50±0.55 | 3.79±0.57 | 3.41±0.47 | 3.22±0.19 | | 3.43±0.20 | 3.26±0.19 |  |
| Blomdin et al. (2016) | Bordoo Valley | BOR 1 | TS-C-12-030 | 41.8122 | 78.1301 | 3766 | 2.6 | 2.7 | 0 | 0.99 | 3.16±0.42 | 07KNSTD |  | 0.57±0.08 | Ar. Mean: 0.64±0.23 ka | 0.64±0.09 | 0.75±0.10 | 0.76±0.09 | 0.61±0.10 | 0.73±0.10 | 0.75±0.14 | 0.72±0.16 | 0.74±0.13 | 0.69±0.09 | | 0.69±0.09 | 0.60±0.08 |  |
| Blomdin et al. (2016) | Bordoo Valley | BOR 1 | TS-C-12-031 | 41.8124 | 78.1305 | 3769 | 2 | 2.7 | 0 | 0.98 | 4.95±0.22 | 07KNSTD |  | 0.90±0.07 | Peak: ! | 1.01±0.07 | 1.07±0.07 | 1.09±0.09 | 0.95±0.09 | 1.08±0.08 | 1.10±0.14 | 1.11±0.16 | 1.08±0.13 | 0.99±0.04 | | 1.02±0.05 | 0.94±0.04 |  |
| Dortch et al., 2013 | Pangong high cirque | N/A | Pang-25 | 33.89 | 78.43 | 5375 | 5 | 2.7 | 0 | 1 | 14.47±3.62 | KNSTD | 0.54±0.11 | 1.79±0.48 | Chi-squared: 0.63 | 1.84±0.5 | 1.90±0.51 | 1.94±0.63 | 1.77±0.52 | 1.59±0.46 | 1.74±0.69 | 1.99±0.69 | 1.72±0.55 | 1.67±0.42 | | 1.70±0.43 | 1.57±0.39 |  |
|  |  |  |  |  |  |  |  |  |  |  |  |  |  |  | Skewness: 0 |  |  |  |  |  |  |  |  |  |  |  |  |  |
| Dortch et al., 2013 | Pangong high cirque | N/A | Pang-26 | 33.89 | 78.43 | 5368 | 5 | 2.7 | 0 | 1 | 4.52±0.90 | KNSTD |  | 0.54±0.11 | Outlier: 2 | 0.54±0.12 | 0.72±0.15 | 0.73±0.16 | 0.64±0.15 | 0.50±0.12 | 0.64±0.18 | 0.63±0.18 | 0.65±0.17 | 0.64±0.13 | | 0.62±0.12 | 0.49±0.10 |  |
| Dortch et al., 2013 | Pangong high cirque | N/A | Pang-27 | 33.89 | 78.43 | 5371 | 5 | 2.7 | 0 | 1 | 5.43±1.81 | KNSTD |  | 0.65±0.22 | Wt. mean: 0.50±0.11 ka | 0.66±0.23 | 0.84±0.30 | 0.86±0.28 | 0.76±0.26 | 0.60±0.23 | 0.77±0.30 | 0.75±0.30 | 0.77±0.27 | 0.75±0.25 | | 0.74±0.25 | 0.59±0.20 |  |
| Dortch et al., 2013 | Pangong high cirque | N/A | Pang-28 | 33.89 | 78.43 | 5363 | 5 | 2.7 | 0 | 1 | 14.47±1.81 | KNSTD |  | 1.8±0.24 | Ar. Mean: 0.54±0.11 ka | 1.84±0.27 | 1.91±0.31 | 1.95±0.34 | 1.77±0.29 | 1.60±0.26 | 1.75±0.46 | 2.00±0.46 | 1.73±0.35 | 1.68±0.21 | | 1.71±0.21 | 1.57±0.20 |  |
| Dortch et al., 2013 | Pangong high cirque | N/A | Pang-29 | 33.89 | 78.43 | 5360 | 4 | 2.7 | 0 | 1 | 3.62±0.90 | KNSTD |  | 0.43±0.11 | Peak: 0.50 ka | 0.43±0.11 | 0.57±0.17 | 0.58±0.18 | 0.51±0.13 | 0.40±0.11 | 0.52±0.15 | 0.50±0.15 | 0.52±0.15 | 0.52±0.13 | | 0.51±0.13 | 0.39±0.10 |  |
| Saha et al., 2018 | Late Holocene | mA2c | LATO-1409 | 33.6851 | 77.5953 | 5314 | 3 | 2.7 | 0 | 0.959 | 265.10±5.64 | 07KNSTD | 0.52±0.20 | 27.56±1.52 | Chi-squared: 24.25 | 29.25±1.52 | 28.30±1.90 | 28.80±2.10 | 29.00±2.10 | 31.10±2.60 | 27.50±3.40 | 27.10±3.40 | 26.90±3.20 | 30.65±0.66 | | 28.48±0.61 | 26.35±0.56 |  |
| Saha et al., 2018 | Late Holocene | mA2c | LATO-1410 | 33.6851 | 77.5953 | 5321 | 2.5 | 2.7 | 0 | 0.959 | 21.86±0.87 | 07KNSTD |  | 3.04±0.21 | Skewness: 0.94 | 2.93±0.21 | 2.91±0.16 | 2.94±0.18 | 2.83±0.21 | 2.53±0.23 | 2.75±0.29 | 2.99±0.29 | 2.73±0.29 | 2.49±0.10 | | 2.88±0.11 | 2.86±0.11 |  |
| Saha et al., 2018 | Late Holocene | mA2c | LATO-1411 | 33.6851 | 77.5952 | 5315 | 2 | 2.7 | 0 | 0.959 | 29.63±1.78 | 07KNSTD |  | 4.06±0.32 | Outlier: 3 | 3.92±0.32 | 3.82±0.41 | 3.91±0.46 | 3.71±0.32 | 3.43±0.35 | 3.53±0.68 | 3.92±0.68 | 3.49±0.51 | 3.37±0.20 | | 3.86±0.23 | 3.86±0.23 |  |
| Saha et al., 2018 | Late Holocene | mA2c | LATO-1412 | 33.685 | 77.5957 | 5315 | 1 | 2.7 | 0 | 0.958 | 2.76±0.15 | 07KNSTD |  | 0.35±0.03 | Wt. mean: 0.45±0.20 ka | 0.34±0.03 | 0.46±0.04 | 0.47±0.05 | 0.42±0.03 | 0.32±0.03 | 0.41±0.07 | 0.40±0.07 | 0.41±0.07 | 0.31±0.02 | | 0.41±0.02 | 0.42±0.02 |  |
| Saha et al., 2018 | Late Holocene | mA2c | LATO-1413 | 33.6849 | 77.5957 | 5317 | 2 | 2.7 | 0 | 0.959 | 5.77±0.20 | 07KNSTD |  | 0.74±0.06 | Ar. Mean: 0.52±0.20 ka | 0.73±0.06 | 0.93±0.06 | 0.94±0.06 | 0.83±0.07 | 0.67±0.06 | 0.85±0.15 | 0.85±0.15 | 0.85±0.11 | 0.66±0.02 | | 0.81±0.03 | 0.83±0.03 |  |
| Saha et al., 2018 | Late Holocene | mA2c | LATO-1414 | 33.6851 | 77.5954 | 5314 | 2 | 2.7 | 0 | 0.96 | 3.76±0.18 | 07KNSTD |  | 0.48±0.03 | Peak: ! | 0.46±0.03 | 0.62±0.06 | 0.64±0.07 | 0.56±0.05 | 0.43±0.04 | 0.56±0.09 | 0.55±0.09 | 0.57±0.08 | 0.43±0.02 | | 0.55±0.03 | 0.56±0.03 |  |
| Seong et al., 2009 | Olimde 8 stage | m6C | MUST-52 | 38.285 | 75.023 | 4580 | 5 | 2.7 | 0 | 1 | 1.18±0.36 | KNSTD | 0.51±0.15 | 0.16±0.06 | Chi-squared: 4.19 | 0.16±0.05 | 0.22±0.08 | 0.23±0.09 | 0.22±0.07 | 0.16±0.06 | 0.21±0.07 | 0.18±0.07 | 0.20±0.08 | 0.00±0.00 | | 0.00±0.00 | 0.00±0.00 |  |
|  |  |  |  |  |  |  |  |  |  |  |  |  |  |  | Skewness: +0.14 |  |  |  |  |  |  |  |  |  |  |  |  |  |
| Seong et al., 2009 | Olimde 8 stage | m6C | MUST-53 | 38.285 | 75.026 | 4568 | 5 | 2.7 | 0 | 1 | 2.89±0.36 | KNSTD |  | 0.43±0.06 | Outlier: 1 | 0.4±0.05 | 0.54±0.07 | 0.55±0.07 | 0.50±0.07 | 0.41±0.07 | 0.51±0.11 | 0.50±0.11 | 0.51±0.09 | 0.50±0.06 | | 0.49±0.06 | 0.40±0.05 |  |
| Seong et al., 2009 | Olimde 8 stage | m6C | MUST-54 | 38.287 | 75.024 | 4524 | 5 | 2.7 | 0 | 1 | 2.26±0.45 | KNSTD |  | 0.34±0.07 | Wt. mean: 0.48±0.15 ka | 0.32±0.07 | 0.44±0.09 | 0.45±0.09 | 0.41±0.08 | 0.32±0.08 | 0.42±0.11 | 0.39±0.11 | 0.41±0.10 | 0.41±0.08 | | 0.40±0.08 | 0.32±0.06 |  |
| Seong et al., 2009 | Olimde 8 stage | m6C | MUST-55 | 38.287 | 75.025 | 4504 | 5 | 2.7 | 0 | 1 | 4.34±0.45 | KNSTD |  | 0.68±0.08 | Ar. Mean: 0.51±0.15 ka | 0.64±0.08 | 0.81±0.10 | 0.82±0.12 | 0.76±0.10 | 0.63±0.09 | 0.78±0.16 | 0.78±0.16 | 0.77±0.13 | 0.74±0.08 | | 0.73±0.08 | 0.62±0.07 |  |
| Seong et al., 2009 | Olimde 8 stage | m6C | MUST-56 | 38.287 | 75.025 | 4511 | 5 | 2.7 | 0 | 1 | 3.71±0.45 | KNSTD |  | 0.57±0.08 | Peak: 0.42 ka ! | 0.54±0.07 | 0.70±0.08 | 0.71±0.10 | 0.66±0.11 | 0.54±0.08 | 0.67±0.13 | 0.65±0.13 | 0.67±0.13 | 0.64±0.08 | | 0.63±0.08 | 0.53±0.06 |  |
| Koppes et al. (2008) | Ala Archa | N/A | KTS98-CS-101 | 42.52 | 74.51 | 3246 | 2 | 2.7 | 0 | 0.929 | 1.20±0.30 | 07KNSTD | 0.49±0.25 | 0.31±0.08 | Chi-squared: ! | 0.32±0.09 | 0.41±0.09 | 0.41±0.10 | 0.32±0.08 | 0.40±0.10 | 0.41±0.11 | 0.37±0.12 | 0.40±0.10 | 0.39±0.10 | | 0.38±0.09 | 0.32±0.08 |  |
|  |  |  |  |  |  |  |  |  |  |  |  |  |  |  | Skewness: ! |  |  |  |  |  |  |  |  |  |  |  |  |  |
|  |  |  |  |  |  |  |  |  |  |  |  |  |  |  | Outlier: 0 |  |  |  |  |  |  |  |  |  |  |  |  |  |
|  |  |  |  |  |  |  |  |  |  |  |  |  |  |  | Wt. mean: 0.43±0.25 ka |  |  |  |  |  |  |  |  |  |  |  |  |  |
|  |  |  |  |  |  |  |  |  |  |  |  |  |  |  | Ar. Mean: 0.49±0.25 ka |  |  |  |  |  |  |  |  |  |  |  |  |  |
| Koppes et al. (2008) | Ala Archa | N/A | KTS98-CS-102 | 42.52 | 74.51 | 3180 | 1.5 | 2.7 | 0 | 0.913 | 2.50±0.40 | 07KNSTD |  | 0.67±0.11 | Peak: ! | 0.73±0.12 | 0.83±0.14 | 0.84±0.13 | 0.70±0.13 | 0.82±0.13 | 0.87±0.19 | 0.85±0.20 | 0.85±0.17 | 0.77±0.12 | | 0.78±0.13 | 0.69±0.11 |  |
| Li et al., 2014 | LIA | Daxi | 1#-10-18 | 43.115 | 86.825 | 3686 | 3 | 2.7 | 0 | 0.972 | 1.66±0.18 | 07KNSTD | 0.33±0.02 | 0.30±0.03 | Chi-squared: 0.49 | 0.33±0.04 | 0.42±0.05 | 0.42±0.06 | 0.33±0.04 | 0.42±0.05 | 0.42±0.08 | 0.37±0.08 | 0.41±0.07 | 0.41±0.04 | | 0.40±0.04 | 0.32±0.04 |  |
|  |  |  |  |  |  |  |  |  |  |  |  |  |  |  | Skewness: -1.19 |  |  |  |  |  |  |  |  |  |  |  |  |  |
|  |  |  |  |  |  |  |  |  |  |  |  |  |  |  | Outlier: 0 |  |  |  |  |  |  |  |  |  |  |  |  |  |
| Li et al., 2014 | LIA | Daxi | 1#-10-19 | 43.115 | 86.825 | 3691 | 2 | 2.7 | 0 | 0.972 | 2.00±0.18 | 07KNSTD |  | 0.35±0.03 | Wt. mean: 0.33±0.02 ka | 0.40±0.04 | 0.50±0.06 | 0.51±0.04 | 0.39±0.05 | 0.49±0.05 | 0.50±0.09 | 0.45±0.09 | 0.49±0.08 | 0.47±0.04 | | 0.47±0.04 | 0.38±0.04 |  |
| Li et al., 2014 | LIA | Daxi | 1#-10-20 | 43.115 | 86.825 | 3694 | 1 | 2.7 | 0 | 0.972 | 1.93±0.45 | 07KNSTD |  | 0.34±0.08 | Ar. Mean: 0.33±0.02 ka | 0.38±0.09 | 0.47±0.13 | 0.48±0.14 | 0.37±0.09 | 0.47±0.10 | 0.47±0.13 | 0.43±0.14 | 0.47±0.13 | 0.46±0.11 | | 0.45±0.10 | 0.37±0.09 |  |
| Li et al., 2014 | LIA | Daxi | 1#-10-21 | 43.115 | 86.825 | 3698 | 3.5 | 2.7 | 0 | 0.972 | 1.84±0.22 | 07KNSTD |  | 0.33±0.04 | Peak: ! | 0.36±0.05 | 0.46±0.05 | 0.47±0.07 | 0.36±0.05 | 0.46±0.06 | 0.46±0.09 | 0.42±0.09 | 0.45±0.08 | 0.44±0.05 | | 0.43±0.05 | 0.36±0.04 |  |
| Hedrick et al., 2011 | PM-3 stage | N/A | India-45 | 33.226 | 78.166 | 5266 | 3 | 2.7 | 0 | 1 | 4.45±0.30 | 07KNSTD | 0.28±0.05 | 0.55±0.05 | Chi-squared: 2.22 | 0.57±0.05 | 0.75±0.07 | 0.76±0.07 | 0.66±0.07 | 0.52±0.05 | 0.67±0.11 | 0.65±0.11 | 0.68±0.11 | 0.66±0.04 | | 0.65±0.04 | 0.51±0.03 |  |
| Hedrick et al., 2011 | PM-3 stage | N/A | India-46 | 33.226 | 78.167 | 5263 | 3 | 2.7 | 0 | 1 | 1.82±0.21 | 07KNSTD |  | 0.22±0.03 | Skewness: -1.09 | 0.23±0.03 | 0.31±0.03 | 0.31±0.05 | 0.29±0.04 | 0.21±0.03 | 0.27±0.05 | 0.26±0.05 | 0.27±0.05 | 0.00±0.00 | | 0.29±0.03 | 0.21±0.02 |  |
| Hedrick et al., 2011 | PM-3 stage | N/A | India-47 | 33.226 | 78.167 | 5257 | 3 | 2.7 | 0 | 1 | 11.74±0.47 | 07KNSTD |  | 1.52±0.1 | Outlier: 3 | 1.57±0.11 | 1.64±0.15 | 1.67±0.16 | 1.53±0.12 | 1.36±0.12 | 1.52±0.30 | 1.70±0.30 | 1.52±0.19 | 1.44±0.06 | | 1.46±0.06 | 1.34±0.05 |  |
| Hedrick et al., 2011 | PM-3 stage | N/A | India-48 | 33.226 | 78.167 | 5267 | 2 | 2.7 | 0 | 1 | 10.22±0.48 | 07KNSTD |  | 1.3±0.09 | Wt. mean: 0.26±0.05 ka | 1.32±0.1 | 1.43±0.12 | 1.46±0.12 | 1.30±0.10 | 1.17±0.11 | 1.33±0.26 | 1.44±0.26 | 1.33±0.16 | 1.25±0.06 | | 1.27±0.06 | 1.15±0.05 |  |
| Hedrick et al., 2011 | PM-3 stage | N/A | India-49 | 33.226 | 78.167 | 5260 | 4 | 2.7 | 0 | 1 | 2.40±0.31 | 07KNSTD |  | 0.29±0.04 | Ar. Mean: 0.28±0.05 ka | 0.3±0.04 | 0.41±0.06 | 0.42±0.07 | 0.38±0.05 | 0.28±0.04 | 0.37±0.07 | 0.36±0.07 | 0.37±0.07 | 0.38±0.05 | | 0.37±0.05 | 0.28±0.04 |  |
| Hedrick et al., 2011 | PM-3 stage | N/A | India-50 | 33.226 | 78.167 | 5265 | 3 | 2.7 | 0 | 1 | 2.55±0.34 | 07KNSTD |  | 0.32±0.05 | Peak: 0.29 ka ! | 0.32±0.05 | 0.44±0.07 | 0.44±0.07 | 0.40±0.06 | 0.30±0.05 | 0.39±0.08 | 0.38±0.08 | 0.39±0.08 | 0.40±0.05 | | 0.39±0.05 | 0.29±0.04 |  |
| Saha et al., 2018 | Late Holocene | mA2c | LATO-1415 | 33.6822 | 77.592 | 5366 | 3 | 2.7 | 0 | 0.95 | 13.31±0.31 | 07KNSTD | 0.26±0.08 | 1.78±0.11 | Chi-squared: 6.78 | 1.72±0.11 | 1.84±0.16 | 1.88±0.17 | 1.71±0.14 | 1.53±0.13 | 1.68±0.32 | 1.92±0.32 | 1.67±0.24 | 1.51±0.04 | | 1.64±0.04 | 1.60±0.04 |  |
|  |  |  |  |  |  |  |  |  |  |  |  |  |  |  | Skewness: +1.70 |  |  |  |  |  |  |  |  |  |  |  |  |  |
| Saha et al., 2018 | Late Holocene | mA1 | LATO-1416 | 33.6822 | 77.5921 | 5358 | 4 | 2.7 | 0 | 0.95 | 1.66±0.18 | 07KNSTD |  | 0.21±0.03 | Outlier: 2 | 0.20±0.03 | 0.28±0.04 | 0.29±0.04 | 0.26±0.04 | 0.19±0.03 | 0.25±0.05 | 0.24±0.05 | 0.25±0.04 | 0.19±0.02 | | 0.26±0.03 | ± |  |
| Saha et al., 2018 | Late Holocene | mA1 | LATO-1417 | 33.6826 | 77.592 | 5348 | 2 | 2.7 | 0 | 0.951 | 2.74±0.10 | 07KNSTD |  | 0.35±0.03 | Wt. mean: 0.26±0.08 ka | 0.34±0.03 | 0.46±0.04 | 0.47±0.04 | 0.42±0.03 | 0.32±0.03 | 0.41±0.07 | 0.40±0.07 | 0.41±0.06 | 0.31±0.01 | | 0.41±0.02 | 0.42±0.02 |  |
| Saha et al., 2018 | Late Holocene | mA1 | LATO-1418 | 33.6826 | 77.5921 | 5351 | 3 | 2.7 | 0 | 0.946 | 1.79±0.14 | 07KNSTD |  | 0.22±0.03 | Ar. Mean: 0.26±0.08 ka | 0.22±0.03 | 0.30±0.03 | 0.31±0.03 | 0.29±0.03 | 0.21±0.02 | 0.27±0.05 | 0.26±0.05 | 0.27±0.05 | 0.21±0.02 | | 0.28±0.02 | ± |  |
| Saha et al., 2018 | Late Holocene | mA1 | LATO-1419 | 33.6827 | 77.592 | 5339 | 3 | 2.7 | 0 | 0.952 | 24.66±1.85 | 07KNSTD |  | 3.43±0.32 | Peak: 0.22 ka ! | 3.31±0.32 | 3.20±0.32 | 3.26±0.35 | 3.15±0.32 | 2.87±0.32 | 3.00±0.48 | 3.31±0.48 | 2.97±0.32 | 2.82±0.21 | | 3.19±0.24 | 3.16±0.24 |  |
| ***Climatic Zone 1b: Arid and semiarid colder climatic region—southern and northeastern Tibet*** | | | | | | | | | | | | | | | | | | | | | | | | | | | | |
| Wang et al. (2013) | Qitai Valley | Group D moraines | DLJ-09 | 35.5577 | 102.7354 | 3828 | 2.1 | 2.65 | 0 | 0.991 | 60.58±1.72 | 07KNSTD | 13.45±0.25 | 13.27±0.66 | Chi-squared: ! | 13.28±0.73 | 13.60±1.10 | 13.80±1.20 | 13.20±1.10 | 13.20±1.10 | 13.50±2.10 | 14.00±1.90 | 13.30±1.90 | 13.12±0.37 | | 13.14±0.37 | 12.79±0.37 |  |
|  |  |  |  |  |  |  |  |  |  |  |  |  |  |  | Skewness: ! |  |  |  |  |  |  |  |  |  |  |  |  |  |
|  |  |  |  |  |  |  |  |  |  |  |  |  |  |  | Outlier: 0 |  |  |  |  |  |  |  |  |  |  |  |  |  |
|  |  |  |  |  |  |  |  |  |  |  |  |  |  |  | Wt. mean: 13.44±0.25 ka |  |  |  |  |  |  |  |  |  |  |  |  |  |
|  |  |  |  |  |  |  |  |  |  |  |  |  |  |  | Ar. Mean: 13.45±0.25 ka |  |  |  |  |  |  |  |  |  |  |  |  |  |
| Wang et al. (2013) | Qitai Valley | Group D moraines | DLJ-17 | 35.5583 | 102.7351 | 3816 | 2.2 | 2.65 | 0 | 0.991 | 61.81±1.71 | 07KNSTD |  | 13.62±0.67 | Peak: 13.44 ka | 13.62±0.74 | 13.90±1.10 | 14.10±1.20 | 13.60±1.10 | 13.60±1.10 | 13.90±2.10 | 14.00±2.00 | 13.70±1.90 | 13.41±0.37 | | 13.46±0.37 | 13.14±0.37 |  |
| Laserre et al. (2002) | N/A | unnamed moraine | LLL1 | 37.53 | 101.85 | 3900 | 4 | 2.7 | 0 | 1 | 33.87±2.34 | LLNL3000 | 13.16±1.05 | 6.18±0.45 | Chi-squared: 1.66 | 6.15±0.44 | 5.78±0.58 | 5.88±0.65 | 5.99±0.58 | 5.99±0.58 | 5.90±0.99 | 6.40±1.20 | 5.78±0.84 | 6.42±0.44 | | 6.34±0.44 | 5.70±0.44 |  |
| Laserre et al. (2002) | N/A | unnamed moraine | LLL2 | 37.53 | 101.85 | 3920 | 4 | 2.7 | 0 | 1 | 47.33±2.34 | LLNL3000 |  | 8.3±0.57 | Skewness: -0.95 | 8.09±0.59 | 7.93±0.75 | 8.09±0.85 | 8.12±0.75 | 8.12±0.75 | 8.10±1.30 | 8.70±1.40 | 8.00±1.20 | 8.41±0.42 | | 8.48±0.42 | 7.89±0.42 |  |
| Laserre et al. (2002) | N/A | unnamed moraine | LLL3 | 37.53 | 101.85 | 3980 | 4 | 2.7 | 0 | 1 | 76.03±3.86 | LLNL3000 |  | 12.79±0.85 | Outlier: 6 | 12.35±0.88 | 12.60±1.20 | 12.80±1.30 | 12.70±1.20 | 12.70±1.20 | 12.70±2.10 | 13.00±2.00 | 12.50±1.80 | 12.46±0.63 | | 12.71±0.65 | 12.30±0.63 |  |
| Laserre et al. (2002) | N/A | unnamed moraine | LLL4 | 37.53 | 101.85 | 3980 | 4 | 2.7 | 0 | 1 | 46.79±2.33 | LLNL3000 |  | 7.98±0.53 | Wt. mean: 13.04±1.05 ka | 7.75±0.52 | 7.59±0.69 | 7.74±0.75 | 7.78±0.69 | 7.78±0.69 | 7.80±1.30 | 8.30±1.30 | 7.60±1.10 | 8.07±0.40 | | 8.16±0.41 | 7.56±0.40 |  |
| Laserre et al. (2002) | N/A | unnamed moraine | LLL5 | 37.53 | 101.85 | 3990 | 4 | 2.7 | 0 | 1 | 85.54±4.69 | LLNL3000 |  | 14.16±0.93 | Ar. Mean:13.16±1.05 ka | 13.69±0.95 | 14.00±1.30 | 14.30±1.40 | 14.20±1.30 | 14.20±1.30 | 14.20±2.20 | 14.60±2.10 | 14.00±2.00 | 13.58±0.75 | | 13.93±0.77 | 13.77±0.75 |  |
| Laserre et al. (2002) | N/A | unnamed moraine | LLL6 | 37.53 | 101.85 | 4000 | 4 | 2.7 | 0 | 1 | 18.93±1.05 | LLNL3000 |  | 3.45±0.25 | Peak: 13.44 ka | 3.5±0.27 | 3.15±0.29 | 3.21±0.29 | 3.37±0.29 | 3.37±0.29 | 3.21±0.48 | 3.49±0.54 | 3.15±0.43 | 3.48±0.19 | | 3.45±0.19 | 3.02±0.19 |  |
| Laserre et al. (2002) | N/A | unnamed moraine | LLL7 | 37.53 | 101.85 | 4000 | 4 | 2.7 | 0 | 1 | 28.30±2.12 | LLNL3000 |  | 5.06±0.4 |  | 5.11±0.4 | 4.76±0.51 | 4.84±0.52 | 4.80±0.51 | 4.80±0.51 | 4.79±0.79 | 5.21±0.68 | 4.70±0.73 | 5.20±0.39 | | 5.11±0.38 | 4.52±0.39 |  |
| Laserre et al. (2002) | N/A | unnamed moraine | LLL8 | 37.53 | 101.85 | 4000 | 4 | 2.7 | 0 | 1 | 68.32±3.95 | LLNL3000 |  | 11.38±0.76 |  | 11.09±0.76 | 11.20±1.10 | 11.40±1.30 | 11.40±1.10 | 11.40±1.10 | 11.40±1.90 | 11.80±1.80 | 11.10±1.70 | 11.30±0.66 | | 11.45±0.66 | 10.93±0.66 |  |
| Laserre et al. (2002) | N/A | unnamed moraine | LLL9 | 37.53 | 101.85 | 4000 | 4 | 2.7 | 0 | 1 | 77.98±4.34 | LLNL3000 |  | 12.96±0.89 |  | 12.55±0.94 | 12.70±1.30 | 13.00±1.40 | 12.90±1.30 | 12.90±1.30 | 12.90±2.10 | 13.30±2.10 | 12.60±1.90 | 12.61±0.70 | | 12.87±0.72 | 12.48±0.70 |  |
| Laserre et al. (2002) | N/A | unnamed moraine | LLL10 | 37.53 | 101.85 | 4000 | 4 | 2.7 | 0 | 1 | 85.94±4.29 | LLNL3000 |  | 14.16±0.89 |  | 13.68±0.9 | 14.00±1.30 | 14.20±1.30 | 14.20±1.30 | 14.20±1.30 | 14.20±2.20 | 14.50±2.10 | 14.00±2.00 | 13.57±0.68 | | 13.93±0.70 | 13.76±0.68 |  |
| Laserre et al. (2002) | N/A | unnamed moraine | LLL10b | 37.53 | 101.85 | 4000 | 4 | 2.7 | 0 | 1 | 81.59±4.20 | LLNL3000 |  | 13.50±0.86 |  | 13.06±0.91 | 13.30±1.30 | 13.60±1.30 | 13.50±1.30 | 13.50±1.30 | 13.50±2.20 | 14.00±2.00 | 13.00±2.00 | 13.03±0.67 | | 13.34±0.69 | 13.06±0.67 |  |
| Laserre et al. (2002) | N/A | unnamed moraine | LLL11 | 37.53 | 101.85 | 4000 | 4 | 2.7 | 0 | 1 | 26.98±1.59 | LLNL3000 |  | 4.83±0.34 |  | 4.88±0.35 | 4.54±0.48 | 4.63±0.49 | 4.64±0.48 | 4.64±0.48 | 4.58±0.77 | 5.02±0.67 | 4.49±0.69 | 4.94±0.29 | | 4.84±0.29 | 4.31±0.29 |  |
| Owen et al. (2003a) | Halong glacial stage | N/A | A29 | 34.84 | 99.58 | 3945 | 5 | 2.7 | 0 | 0.994 | 66.00±2.00 | LLNL3000 | 12.89±1.26 | 12.36±0.68 | Chi-squared: 4.68 | 12.46±0.77 | 12.70±1.10 | 12.90±1.10 | 12.00±1.10 | 12.00±1.10 | 12.50±1.90 | 13.10±1.90 | 12.30±1.70 | 12.40±0.38 | | 12.34±0.38 | 11.83±0.38 |  |
| Owen et al. (2003a) | Halong glacial stage | N/A | A30 | 34.84 | 99.58 | 3945 | 5 | 2.7 | 0 | 0.994 | 74.00±2.00 | LLNL3000 |  | 13.74±0.68 | Skewness: -1.47 | 13.8±0.76 | 14.20±1.10 | 14.40±1.20 | 13.70±1.10 | 13.70±1.10 | 14.00±2.10 | 14.50±1.90 | 13.80±1.90 | 13.53±0.37 | | 13.57±0.37 | 13.27±0.37 |  |
| Owen et al. (2003a) | Halong glacial stage | N/A | A31 | 34.84 | 99.57 | 3970 | 5 | 2.7 | 0 | 0.995 | 71.00±2.00 | LLNL3000 |  | 13.06±0.66 | Outlier: 0 | 13.13±0.74 | 13.40±1.10 | 13.70±1.20 | 13.00±1.10 | 13.00±1.10 | 13.20±2.10 | 13.80±1.90 | 13.00±1.80 | 12.96±0.37 | | 12.97±0.37 | 12.55±0.37 |  |
| Owen et al. (2003a) | Halong glacial stage | N/A | A32 | 34.84 | 99.57 | 3980 | 5 | 2.7 | 0 | 0.996 | 77.00±3.00 | LLNL3000 |  | 13.98±0.79 | Wt. mean: 13.28±1.26 ka | 14.01±0.85 | 14.40±1.10 | 14.60±1.20 | 14.00±1.10 | 14.00±1.10 | 14.20±2.10 | 15.00±2.00 | 14.00±1.90 | 13.72±0.54 | | 13.79±0.54 | 13.53±0.54 |  |
| Owen et al. (2003a) | Halong glacial stage | N/A | A37 | 34.84 | 99.55 | 4045 | 5 | 2.7 | 0 | 0.996 | 77.00±4.00 | LLNL3000 |  | 13.58±0.86 | Ar. Mean: 12.89±1.26 ka | 13.61±0.91 | 14.00±1.30 | 14.20±1.30 | 13.50±1.30 | 13.50±1.30 | 13.70±2.20 | 14.00±2.00 | 13.00±2.00 | 13.33±0.70 | | 13.41±0.70 | 13.09±0.70 |  |
| Owen et al. (2003a) | Halong glacial stage | N/A | A38 | 34.84 | 99.55 | 4055 | 5 | 2.7 | 0 | 0.995 | 59.00±2.00 | LLNL3000 |  | 10.60±0.53 | Peak: 13.40 ka | 10.71±0.57 | 11.00±1.00 | 10.90±1.10 | 10.35±1.00 | 10.35±1.00 | 10.50±1.70 | 11.10±1.70 | 10.30±1.60 | 10.92±0.37 | | 10.79±0.37 | 9.98±0.37 |  |
| Owen et al. (2005) | Intermediate age | Un-named moraine | PR35 | 28.8947 | 90.2299 | 4914 | 5 | 2.7 | 0 | 0.972 | 81.90±2.47 | LLNL3000 | 11.47±0.70 | 11.66±0.6 | Chi-squared: 1.56 | 12.13±0.76 | 12.24±0.99 | 12.50±1.10 | 11.51±0.99 | 11.51±0.99 | 11.30±1.80 | 12.10±1.70 | 11.20±1.70 | 11.88±0.36 | | 11.89±0.36 | 11.10±0.36 |  |
|  |  |  |  |  |  |  |  |  |  |  |  |  |  |  | Skewness: +0.80 |  |  |  |  |  |  |  |  |  |  |  |  |  |
|  |  |  |  |  |  |  |  |  |  |  |  |  |  |  | Outlier: 0 |  |  |  |  |  |  |  |  |  |  |  |  |  |
| Owen et al. (2005) | Intermediate age | Un-named moraine | PR36 | 28.8957 | 90.2294 | 4897 | 5 | 2.7 | 0 | 0.964 | 74.80±2.47 | LLNL3000 |  | 10.99±0.5 | Wt. mean: 11.32±0.70 ka | 11.32±0.58 | 11.00±1.00 | 11.60±1.10 | 10.66±1.00 | 10.66±1.00 | 10.50±1.80 | 11.30±1.70 | 10.40±1.60 | 11.29±0.37 | | 11.26±0.37 | 10.29±0.37 |  |
| Owen et al. (2005) | Intermediate age | Un-named moraine | PR37 | 28.8958 | 90.2293 | 4891 | 5 | 2.7 | 0 | 0.968 | 85.60±2.11 | LLNL3000 |  | 12.38±0.65 | Ar. Mean: 11.47±0.70 ka | 12.89±0.74 | 13.00±1.10 | 13.20±1.20 | 12.18±1.10 | 12.18±1.10 | 12.00±1.80 | 12.80±1.90 | 11.90±1.60 | 12.54±0.31 | | 12.52±0.31 | 11.77±0.31 |  |
| Owen et al. (2005) | Intermediate age | Un-named moraine | PR38 | 28.896 | 90.2292 | 4878 | 5 | 2.7 | 0 | 0.968 | 73.20±1.77 | LLNL3000 |  | 10.86±0.47 | Peak: 11.04 ka | 11.2±0.5 | 11.19±0.95 | 11.40±1.10 | 10.47±0.95 | 10.47±0.95 | 10.30±1.70 | 11.10±1.70 | 10.20±1.60 | 11.18±0.27 | | 11.14±0.27 | 10.12±0.27 |  |
| Owen et al. (2003c) | Gangshiga valley | Holocene moraine | Q21 | 37.7 | 101.59 | 4190 | 5 | 2.7 | 0 | 0.98 | 64.00±2.00 | LLNL3000 | 10.08±0.53 | 10.08±0.53 | Chi-squared: ! | 9.75±0.6 | 9.50±0.90 | 10.00±1.00 | 9.86±0.90 | 9.86±0.90 | 9.70±1.60 | 10.20±1.60 | 9.50±1.50 | 10.06±0.32 | | 10.19±0.32 | 9.52±0.32 |  |
|  |  |  |  |  |  |  |  |  |  |  |  |  |  |  | Skewness: ! |  |  |  |  |  |  |  |  |  |  |  |  |  |
|  |  |  |  |  |  |  |  |  |  |  |  |  |  |  | Outlier: 1 |  |  |  |  |  |  |  |  |  |  |  |  |  |
|  |  |  |  |  |  |  |  |  |  |  |  |  |  |  | Wt. mean: 10.08±0.53 ka |  |  |  |  |  |  |  |  |  |  |  |  |  |
|  |  |  |  |  |  |  |  |  |  |  |  |  |  |  | Ar. Mean: 10.08±0.53 ka |  |  |  |  |  |  |  |  |  |  |  |  |  |
| Owen et al. (2003c) | Gangshiga valley | Holocene moraine | Q23 | 37.7 | 101.46 | 4180 | 5 | 2.7 | 0 | 1 | 28.00±1.00 | LLNL3000 |  | 4.61±0.25 | Peak: ! | 4.63±0.28 | 4.26±0.41 | 4.36±0.44 | 4.43±0.41 | 4.43±0.41 | 4.29±0.72 | 4.75±0.72 | 4.19±0.64 | 4.58±0.16 | | 4.56±0.16 | 4.10±0.16 |  |
| Owen et al. (2003a) | Halong glacial stage | N/A | A1 | 34.89 | 99.44 | 4285 | 5 | 2.7 | 0 | 0.997 | 26.00±1.00 | LLNL3000 | 9.48±1.70 | 4.46±0.26 | Chi-squared: ! | 4.65±0.29 | 4.21±0.42 | 4.30±0.45 | 4.26±0.42 | 4.26±0.42 | 4.11±0.72 | 4.61±0.76 | 4.04±0.64 | 4.58±0.18 | | 4.44±0.17 | 3.90±0.18 |  |
|  |  |  |  |  |  |  |  |  |  |  |  |  |  |  | Skewness: ! |  |  |  |  |  |  |  |  |  |  |  |  |  |
| Owen et al. (2003a) | Halong glacial stage | N/A | A2 | 34.88 | 99.45 | 4335 | 5 | 2.7 | 0 | 0.998 | 34.00±1.00 | LLNL3000 |  | 5.54±0.26 | Outlier: 3 | 5.67±0.27 | 5.29±0.35 | 5.37±0.38 | 5.29±0.35 | 5.29±0.35 | 5.20±0.70 | 5.60±0.60 | 5.11±0.62 | 5.78±0.17 | | 5.65±0.17 | 4.97±0.17 |  |
| Owen et al. (2003a) | Halong glacial stage | N/A | A3 | 34.88 | 99.45 | 4340 | 5 | 2.7 | 0 | 0.995 | 11.00±1.00 | LLNL3000 |  | 1.83±0.2 | Wt. mean: 9.22±1.70 ka | 1.89±0.21 | 1.80±0.21 | 1.84±0.24 | 1.84±0.21 | 1.84±0.21 | 1.78±0.31 | 1.99±0.43 | 1.76±0.28 | 1.79±0.16 | | 1.77±0.16 | 1.61±0.16 |  |
| Owen et al. (2003a) | Halong glacial stage | N/A | A4 | 34.88 | 99.43 | 4285 | 5 | 2.7 | 0 | 0.998 | 67.00±2.00 | LLNL3000 |  | 10.68±0.51 | Ar. Mean: 9.48±1.70 ka | 10.71±0.55 | 10.68±0.98 | 10.90±1.10 | 10.43±0.98 | 10.43±0.98 | 10.40±1.70 | 11.10±1.70 | 10.20±1.50 | 10.93±0.33 | | 10.87±0.33 | 10.06±0.33 |  |
| Owen et al. (2003a) | Halong glacial stage | N/A | A5 | 34.88 | 99.43 | 4285 | 5 | 2.7 | 0 | 0.998 | 52.00±1.00 | LLNL3000 |  | 8.28±0.41 | Peak: ! | 8.27±0.5 | 8.09±0.69 | 8.25±0.74 | 8.00±0.69 | 8.00±0.69 | 8.00±1.20 | 8.60±1.30 | 7.80±1.10 | 8.57±0.17 | | 8.49±0.16 | 7.80±0.17 |  |
| Owen et al. (2006b) | N/A | M2 moraines | PR1 | 35.7046 | 94.2506 | 4546 | 5 | 2.7 | 0 | 0.97 | 53.08±5.61 | LLNL3000 | 8.04±0.74 | 7.52±0.81 | Chi-squared: ! | 7.4±0.77 | 7.34±0.98 | 7.00±1.00 | 7.00±0.98 | 7.00±0.98 | 7.20±1.40 | 7.80±1.40 | 7.10±1.30 | 7.58±0.80 | | 7.67±0.81 | 7.07±0.80 |  |
|  |  |  |  |  |  |  |  |  |  |  |  |  |  |  | Skewness: ! |  |  |  |  |  |  |  |  |  |  |  |  |  |
|  |  |  |  |  |  |  |  |  |  |  |  |  |  |  | Outlier: 1 |  |  |  |  |  |  |  |  |  |  |  |  |  |
|  |  |  |  |  |  |  |  |  |  |  |  |  |  |  | Wt. mean: 8.13±0.74 ka |  |  |  |  |  |  |  |  |  |  |  |  |  |
| Owen et al. (2006b) | N/A | M2 moraines | PR2 | 35.7049 | 94.2506 | 4548 | 5 | 2.7 | 0 | 0.97 | 129.72±5.77 | LLNL3000 |  | 17.54±1.06 | Ar. Mean: 8.04±0.74 ka | 16.95±1.09 | 17.50±1.40 | 17.90±1.60 | 17.60±1.40 | 17.60±1.40 | 17.20±2.60 | 17.60±2.50 | 16.90±2.30 | 16.43±0.73 | | 17.16±0.77 | 17.32±0.73 |  |
| Owen et al. (2006b) | N/A | M2 moraines | PR3 | 35.704 | 94.2503 | 4588 | 5 | 2.7 | 0 | 0.97 | 61.90±3.69 | LLNL3000 |  | 8.56±0.68 | Peak: 8.25 ka | 8.39±0.73 | 8.30±0.90 | 8.52±0.99 | 8.29±0.90 | 8.29±0.90 | 8.20±1.40 | 8.80±1.50 | 8.00±1.20 | 8.74±0.52 | | 8.79±0.53 | 8.09±0.52 |  |
| Owen et al. (2005) | Youngest | Un-named moraine | PR31 | 28.8907 | 90.2281 | 4980 | 5 | 2.7 | 0 | 0.963 | 25.90±2.38 | LLNL3000 | 3.28±0.74 | 4.12±0.39 | Chi-squared: 6.20 | 4.41±0.43 | 3.82±0.55 | 3.90±0.58 | 3.86±0.55 | 3.86±0.55 | 3.51±0.67 | 3.90±0.80 | 3.49±0.61 | 4.24±0.39 | | 4.19±0.39 | 3.43±0.39 |  |
|  |  |  |  |  |  |  |  |  |  |  |  |  |  |  | Skewness: +1.38 |  |  |  |  |  |  |  |  |  |  |  |  |  |
|  |  |  |  |  |  |  |  |  |  |  |  |  |  |  | Outlier: 0 |  |  |  |  |  |  |  |  |  |  |  |  |  |
|  |  |  |  |  |  |  |  |  |  |  |  |  |  |  | Wt. mean: 3.05±0.74 ka |  |  |  |  |  |  |  |  |  |  |  |  |  |
| Owen et al. (2005) | Youngest | Un-named moraine | PR33 | 28.8892 | 90.2285 | 4994 | 5 | 2.7 | 0 | 0.951 | 18.50±1.01 | LLNL3000 |  | 3.02±0.22 | Ar. Mean: 3.28±0.74 ka | 3.23±0.24 | 2.82±0.19 | 2.86±0.21 | 2.90±0.19 | 2.90±0.19 | 2.64±0.36 | 2.88±0.31 | 2.64±0.32 | 3.08±0.17 | | 3.07±0.17 | 2.47±0.17 |  |
| Owen et al. (2005) | Youngest | Un-named moraine | PR34 | 28.8889 | 90.2285 | 4989 | 5 | 2.7 | 0 | 0.957 | 16.70±1.09 | LLNL3000 |  | 2.7±0.23 | Peak: ! | 2.9±0.25 | 2.60±0.22 | 2.64±0.24 | 2.64±0.22 | 2.64±0.22 | 2.41±0.37 | 2.67±0.39 | 2.42±0.33 | 2.79±0.18 | | 2.80±0.18 | 2.22±0.18 |  |
| ***Climatic Zone 2a: Transitional climatic region*—*western Himalaya*** | | | | | | | | | | | | | | | | | | | | | | | | | | | | |
| Owen et al. (2010) | N/A | M5 | Na104 | 30.4365 | 81.4479 | 5499 | 2 | 2.7 | 0 | 1 | 132.16±3.34 | 07KNSTD | 15.30±0.60 | 15.26±0.81 | Chi-squared: 0.54 | 15.11±0.70 | 16.00±0.38 | 16.30±0.38 | 15.00±0.34 | 15.00±0.38 | 15.00±0.32 | 15.20±0.31 | 14.50±0.32 | 14.55±0.37 | | 14.89±0.37 | 14.74±0.37 |  |
|  |  |  |  |  |  |  |  |  |  |  |  |  |  |  | Skewness: +0.28 |  |  |  |  |  |  |  |  |  |  |  |  |  |
| Owen et al. (2010) | N/A | M5 | Na105 | 30.4364 | 81.4475 | 5501 | 1 | 2.7 | 0 | 1 | 79.56±2.02 | 07KNSTD |  | 9.85±0.56 | Outlier: 2 | 9.54±0.53 | 9.83±0.27 | 10.04±0.27 | 8.97±0.25 | 8.93±0.23 | 8.80±0.24 | 9.50±0.25 | 8.70±0.23 | 10.02±0.26 | | 9.95±0.26 | 8.78±0.22 |  |
| Owen et al. (2010) | N/A | M5 | Na106 | 30.4363 | 81.4484 | 5499 | 2.5 | 2.7 | 0 | 1 | 137.55±3.55 | 07KNSTD |  | 15.91±0.88 | Wt. mean: 15.23±0.60 ka | 15.73±0.76 | 16.70±0.38 | 17.00±0.38 | 15.70±0.38 | 15.70±0.41 | 15.30±0.34 | 15.80±0.36 | 15.10±0.33 | 15.07±0.39 | | 15.45±0.39 | 15.41±0.40 |  |
| Owen et al. (2010) | N/A | M5 | Na107 | 30.4365 | 81.4488 | 5500 | 2 | 2.7 | 0 | 1 | 165.76±4.02 | 07KNSTD |  | 18.73±0.99 | Ar. Mean: 15.30±0.60 ka | 18.60±0.88 | 19.40±0.33 | 19.70±0.32 | 18.50±0.39 | 18.80±0.46 | 18.00±0.38 | 18.40±0.35 | 17.80±0.38 | 17.80±0.43 | | 18.21±0.43 | 18.49±0.45 |  |
| Owen et al. (2010) | N/A | M5 | Na108 | 30.4373 | 81.4464 | 5497 | 1 | 2.7 | 0 | 0.989 | 125.31±3.15 | 07KNSTD |  | 14.72±0.75 | Peak: 15.18 ka | 14.50±0.67 | 15.30±0.34 | 15.60±0.35 | 14.40±0.33 | 14.30±0.36 | 14.00±0.32 | 14.60±0.30 | 13.90±0.31 | 13.91±0.35 | | 14.25±0.35 | 14.02±0.35 |  |
| This study | Lateglacial | Mk5 | S19 | 32.3833 | 77.2783 | 3255 | 2 | 2.7 | 0 | 1 | 22.80±0.69 | 07KNSTD | 14.65±2.06 | 8.92±0.63 | Chi-squared: ! | 8.33±0.46 | 9.20±0.29 | 9.28±0.29 | 7.96±0.24 | 8.00±0.24 | 9.10±0.28 | 9.60±0.29 | 9.00±0.28 | 9.18±0.28 | | 8.51±0.28 | 7.85±0.24 |  |
| This study | Lateglacial | Mk5 | S20 | 32.3833 | 77.2783 | 3258 | 2 | 2.7 | 0 | 1 | 4.73±0.45 | 07KNSTD |  | 2.11±0.25 | Skewness: ! | 1.95±0.22 | 2.21±0.22 | 2.23±0.21 | 1.91±0.18 | 1.73±0.17 | 2.15±0.22 | 2.39±0.21 | 2.16±0.22 | 1.94±0.19 | | 1.83±0.19 | 1.70±0.16 |  |
| This study | Lateglacial | Mk5 | S21 | 32.3833 | 77.2783 | 3256 | 2 | 2.7 | 0 | 1 | 32.03±1.14 | 07KNSTD |  | 13.19±0.78 | Outlier: 4 | 12.54±0.72 | 14.00±0.45 | 14.10±0.45 | 12.00±0.43 | 12.20±0.44 | 14.00±0.45 | 14.20±0.43 | 13.70±0.45 | 12.81±0.46 | | 12.41±0.46 | 11.99±0.43 |  |
| This study | Lateglacial | Mk5 | S22 | 32.3833 | 77.2783 | 3268 | 2 | 2.7 | 0 | 1 | 44.85±1.78 | 07KNSTD |  | 16.11±1.01 | Wt. mean: 14.28±2.06 ka | 15.33±0.86 | 17.00±0.60 | 17.20±0.60 | 15.30±0.56 | 15.30±0.61 | 16.80±0.61 | 17.20±0.59 | 16.70±0.60 | 15.31±0.61 | | 14.93±0.61 | 15.02±0.60 |  |
| This study | Lateglacial | Mk5 | S23 | 32.3833 | 77.2783 | 3272 | 2 | 2.7 | 0 | 1 | 3.16±0.30 | 07KNSTD |  | 1.28±0.14 | Ar. Mean: 14.65±2.06 ka | 1.20±0.13 | 1.38±0.12 | 1.40±0.10 | 1.24±0.10 | 1.08±0.10 | 1.35±0.12 | 1.45±0.15 | 1.36±0.12 | 1.21±0.11 | | 1.17±0.11 | 1.06±0.10 |  |
| This study | Lateglacial | Mk5 | S24 | 32.3833 | 77.2783 | 3274 | 3 | 2.7 | 0 | 1 | 3.06±3.93 | 07KNSTD |  | 1.25±1.6 | Peak: ! | 1.16±1.49 | 1.40±1.61 | 1.40±1.66 | 1.20±1.28 | 1.10±1.35 | 1.30±1.51 | 1.40±1.93 | 1.30±1.52 | 1.18±1.52 | | 1.14±1.52 | 1.03±1.33 |  |
| Owen et al. (2001) | Kulti glacial stage | N/A | L44 | 32.4 | 77.6 | 4070 | 5 | 2.7 | 0 | 0.99 | 66.21±1.90 | LLNL3000 | 14.45±0.70 | 14.45±0.7 | ! | 14.83±0.8 | 15.60±1.20 | 15.80±1.30 | 14.40±1.10 | 14.30±1.30 | 15.10±2.10 | 15.60±2.10 | 15.00±1.80 | 14.08±0.41 | | 14.07±0.41 | 14.02±0.40 |  |
| Scherler et al. (2010) | Tons Valley | (location C) | DS6-27A | 31.1246 | 78.3825 | 3010 | 2 | 2.7 | 0 | 0.96 | 34.84±0.54 | 07KNSTD | 14.06±0.10 | 13.99±0.64 | Chi-squared: ! | 14.93±0.76 | 15.70±1.10 | 15.70±1.20 | 13.90±1.10 | 13.90±1.10 | 15.50±2.20 | 15.90±2.20 | 15.40±1.90 | 14.20±0.22 | | 13.68±0.21 | 13.59±0.22 |  |
|  |  |  |  |  |  |  |  |  |  |  |  |  |  |  | Skewness: ! |  |  |  |  |  |  |  |  |  |  |  |  |  |
|  |  |  |  |  |  |  |  |  |  |  |  |  |  |  | Outlier: 0 |  |  |  |  |  |  |  |  |  |  |  |  |  |
|  |  |  |  |  |  |  |  |  |  |  |  |  |  |  | Wt. mean: 14.06±0.10 ka |  |  |  |  |  |  |  |  |  |  |  |  |  |
|  |  |  |  |  |  |  |  |  |  |  |  |  |  |  | Ar. Mean: 14.06±0.10 ka |  |  |  |  |  |  |  |  |  |  |  |  |  |
| Scherler et al. (2010) | Tons Valley | (location C) | DS6-27B | 31.1246 | 78.3825 | 3010 | 2 | 2.7 | 0 | 0.96 | 35.25±0.54 | 07KNSTD |  | 14.13±0.63 | Peak: 14.06 ka | 15.05±0.76 | 15.80±1.10 | 15.90±1.30 | 14.10±1.10 | 14.10±1.10 | 15.60±2.20 | 16.10±2.30 | 15.60±1.90 | 14.37±0.22 | | 13.80±0.21 | 13.75±0.22 |  |
| Owen et al. (2001) | Kulti glacial stage | N/A | L24 | 32.5 | 77 | 2985 | 5 | 2.7 | 0 | 0.86 | 7.09±0.35 | LLNL3000 | 14.03±0.16 | 3.6±0.23 | Chi-squared: 0.05 | 3.93±0.28 | 3.76±0.39 | 3.79±0.42 | 3.40±0.30 | 3.16±0.31 | 3.72±0.74 | 4.06±0.74 | 3.71±0.56 | 3.74±0.18 | | 3.47±0.17 | 3.10±0.15 |  |
|  |  |  |  |  |  |  |  |  |  |  |  |  |  |  | Skewness: ! |  |  |  |  |  |  |  |  |  |  |  |  |  |
|  |  |  |  |  |  |  |  |  |  |  |  |  |  |  | Outlier: 2 |  |  |  |  |  |  |  |  |  |  |  |  |  |
| Owen et al. (2001) | Kulti glacial stage | N/A | L25 | 32.5 | 77 | 2985 | 5 | 2.7 | 0 | 0.93 | 8.04±0.52 | LLNL3000 |  | 3.77±0.28 | Wt. mean: 14.02±0.16 ka | 4.11±0.34 | 3.96±0.47 | 4.00±0.50 | 3.57±0.35 | 3.31±0.36 | 3.90±0.79 | 4.28±0.79 | 3.90±0.64 | 3.92±0.25 | | 3.66±0.24 | 3.25±0.21 |  |
| Owen et al. (2001) | Kulti glacial stage | N/A | L28 | 32.5 | 77 | 3000 | 5 | 2.7 | 0 | 0.9 | 33.02±1.04 | LLNL3000 |  | 14.14±0.73 | Ar. Mean: 14.03±0.16 ka | 14.95±0.84 | 15.80±1.20 | 15.90±1.30 | 14.10±1.20 | 14.00±1.20 | 15.70±2.30 | 16.00±2.30 | 16.00±2.00 | 14.24±0.45 | | 13.77±0.43 | 13.70±0.43 |  |
| Owen et al. (2001) | Kulti glacial stage | N/A | L29 | 32.5 | 77 | 2985 | 5 | 2.7 | 0 | 0.97 | 34.66±0.95 | LLNL3000 |  | 13.91±0.7 | Peak: 14.03 ka | 14.76±0.8 | 15.50±1.20 | 15.60±1.20 | 13.80±1.10 | 13.70±1.20 | 15.40±2.20 | 15.80±2.20 | 15.30±1.90 | 14.02±0.39 | | 13.58±0.37 | 13.47±0.37 |  |
| Owen et al. (2001) | Kulti glacial stage | N/A | L10 | 32.3 | 77.2 | 2390 | 5 | 2.7 | 0 | 0.99 | 25.76±1.99 | LLNL3000 | 13.95±0.88 | 14.57±1.18 | Chi-squared: 1.11 | 15.67±1.35 | 16.60±1.80 | 16.60±1.90 | 14.50±1.60 | 14.40±1.70 | 16.70±2.60 | 16.90±2.60 | 16.60±2.50 | 14.98±1.16 | | 14.11±1.09 | 14.15±1.10 |  |
|  |  |  |  |  |  |  |  |  |  |  |  |  |  |  | Skewness: ! |  |  |  |  |  |  |  |  |  |  |  |  |  |
|  |  |  |  |  |  |  |  |  |  |  |  |  |  |  | Outlier: 0 |  |  |  |  |  |  |  |  |  |  |  |  |  |
|  |  |  |  |  |  |  |  |  |  |  |  |  |  |  | Wt. mean: 13.64±0.88 ka |  |  |  |  |  |  |  |  |  |  |  |  |  |
|  |  |  |  |  |  |  |  |  |  |  |  |  |  |  | Ar. Mean: 13.95±0.88 ka |  |  |  |  |  |  |  |  |  |  |  |  |  |
| Owen et al. (2001) | Kulti glacial stage | N/A | L12 | 32.3 | 77.2 | 2415 | 5 | 2.7 | 0 | 0.99 | 23.77±0.78 | LLNL3000 |  | 13.33±0.68 | Peak: 13.47 ka | 14.41±0.8 | 15.20±1.10 | 15.20±1.20 | 13.20±1.10 | 13.10±1.20 | 15.20±2.10 | 15.50±2.10 | 15.10±1.90 | 13.79±0.45 | | 13.05±0.43 | 12.85±0.42 |  |
| Barnard et al. (2004b) | N/A | Moraine m2 | NDL15 | 30.46 | 80.13 | 3720 | 5 | 2.7 | 0 | 0.98 | 9.20±1.30 | LLNL3000 | 13.71±0.69 | 2.50±0.4 | Chi-squared: ! | 2.75±0.44 | 2.69±0.37 | 2.71±0.37 | 2.46±0.37 | 2.46±0.37 | 2.58±0.49 | 2.81±0.44 | 2.59±0.45 | 2.63±0.37 | | 2.54±0.36 | 2.14±0.37 |  |
| Barnard et al. (2004b) | N/A | Moraine m2 | NDL16 | 30.45 | 80.13 | 3817 | 5 | 2.7 | 0 | 0.98 | 0.40±0.20 | LLNL3000 |  | 0.09±0.05 | Skewness: ! | 0.09±0.05 | 0.14±0.08 | 0.14±0.08 | 0.12±0.08 | 0.12±0.08 | 0.12±0.07 | 0.12±0.07 | 0.13±0.07 | 0.11±0.05 | | 0.10±0.05 | 0.08±0.04 |  |
| Barnard et al. (2004b) | N/A | Moraine m2 | NDL23 | 30.45 | 80.14 | 3968 | 5 | 2.7 | 0 | 0.99 | 17.60±0.60 | LLNL3000 |  | 4.14±0.23 | Outlier: 5 | 4.5±0.27 | 4.30±0.40 | 4.32±0.42 | 3.90±0.40 | 3.90±0.40 | 4.04±0.69 | 4.45±0.75 | 4.04±0.62 | 4.29±0.15 | | 4.13±0.14 | 3.57±0.15 |  |
| Barnard et al. (2004b) | N/A | Moraine m2 | NDL29 | 30.43 | 80.16 | 3497 | 5 | 2.7 | 0 | 0.97 | 50.00±1.50 | LLNL3000 |  | 13.71±0.69 | Wt. mean: 13.71±0.69 ka | 14.51±0.78 | 15.10±1.10 | 15.30±1.20 | 13.60±1.10 | 13.60±1.10 | 14.80±2.10 | 15.00±2.00 | 14.70±1.80 | 13.83±0.42 | | 13.46±0.41 | 13.29±0.42 |  |
| Barnard et al. (2004b) | N/A | Moraine m2 | NDL30 | 30.43 | 80.16 | 3476 | 5 | 2.7 | 0 | 0.97 | 17.10±0.50 | LLNL3000 |  | 5.21±0.25 | Ar. Mean: 13.71±0.69 ka | 5.59±0.28 | 5.41±0.34 | 5.45±0.37 | 4.88±0.34 | 4.88±0.34 | 5.27±0.69 | 5.58±0.59 | 5.27±0.61 | 5.56±0.16 | | 5.27±0.15 | 4.59±0.16 |  |
| Barnard et al. (2004b) | N/A | Moraine m2 | NDL32 | 30.41 | 80.16 | 3397 | 5 | 2.7 | 0 | 0.97 | 12.10±0.50 | LLNL3000 |  | 3.94±0.23 | Peak: ! | 4.32±0.29 | 4.09±0.42 | 4.13±0.43 | 3.73±0.42 | 3.73±0.42 | 3.95±0.68 | 4.33±0.74 | 3.96±0.61 | 4.13±0.17 | | 3.91±0.16 | 3.39±0.17 |  |
| Murari et al. (2014) | mm1 | N/A | KAL25 | 30.7283 | 79.0198 | 4405 | 1.5 | 2.7 | 0 | 0.98 | 69.96±3.50 | 07KNSTD | 13.62±0.66 | 13.36±0.82 | Chi-squared: 0.67 | 13.81±0.91 | 14.50±1.20 | 14.70±1.20 | 13.20±1.20 | 13.20±1.20 | 13.80±2.10 | 14.00±2.00 | 13.70±1.90 | 13.21±0.66 | | 13.18±0.66 | 12.87±0.66 |  |
|  |  |  |  |  |  |  |  |  |  |  |  |  |  |  | Skewness: +0.41 |  |  |  |  |  |  |  |  |  |  |  |  |  |
|  |  |  |  |  |  |  |  |  |  |  |  |  |  |  | Outlier: 0 |  |  |  |  |  |  |  |  |  |  |  |  |  |
| Murari et al. (2014) | mm1 | N/A | KAL26 | 30.7281 | 79.0198 | 4412 | 3 | 2.7 | 0 | 0.979 | 66.50±2.66 | 07KNSTD |  | 12.9±0.75 | Wt. mean: 13.58±0.66 ka | 13.3±0.8 | 14.00±1.10 | 14.20±1.20 | 12.70±1.10 | 12.70±1.10 | 13.00±2.00 | 13.80±1.90 | 13.10±1.80 | 12.88±0.52 | | 12.81±0.51 | 12.35±0.52 |  |
| Murari et al. (2014) | mm1 | N/A | KAL27 | 30.728 | 79.0189 | 4411 | 2 | 2.7 | 0 | 0.972 | 71.69±2.87 | 07KNSTD |  | 13.78±0.76 | Ar. Mean:13.62±0.66 ka | 14.21±0.84 | 14.90±1.10 | 15.10±1.20 | 13.70±1.10 | 13.70±1.10 | 14.20±2.10 | 14.70±1.90 | 14.10±1.80 | 13.60±0.55 | | 13.57±0.54 | 13.31±0.55 |  |
| Murari et al. (2014) | mm1 | N/A | KAL28 | 30.7271 | 79.0205 | 4378 | 3 | 2.7 | 0 | 0.98 | 74.41±3.72 | 07KNSTD |  | 14.45±0.86 | Peak: 13.53 ka | 14.89±0.96 | 15.60±1.30 | 15.80±1.40 | 14.40±1.30 | 14.40±1.30 | 14.90±2.10 | 15.40±2.10 | 14.80±1.90 | 14.12±0.71 | | 14.12±0.71 | 14.03±0.71 |  |
| Lee et al., 2013 | Anantick stage | ST-3 | NK1 | 34.0466 | 75.9473 | 3506 | 3.5 | 2.7 | 0 | 0.97 | 66.36±1.41 | 07KNSTD | 13.55±0.88 | 18.32±0.86 | Chi-squared: ! | 18.67±0.98 | 19.60±1.20 | 19.70±1.30 | 18.30±1.20 | 18.30±1.20 | 19.50±2.50 | 19.70±2.30 | 19.30±2.20 | 17.88±0.38 | | 17.76±0.38 | 18.13±0.38 |  |
|  |  |  |  |  |  |  |  |  |  |  |  |  |  |  | Skewness: ! |  |  |  |  |  |  |  |  |  |  |  |  |  |
|  |  |  |  |  |  |  |  |  |  |  |  |  |  |  | Outlier: 2 |  |  |  |  |  |  |  |  |  |  |  |  |  |
| Lee et al., 2013 | Anantick stage | ST-3 | NK2 | 34.0466 | 75.9473 | 3506 | 4.5 | 2.7 | 0 | 0.95 | 58.09±2.28 | 07KNSTD |  | 16.62±0.95 | Wt. mean: 13.53±0.88 ka | 17.01±1.06 | 18.00±1.40 | 18.20±1.50 | 16.60±1.40 | 16.60±1.40 | 17.90±2.60 | 18.20±2.40 | 17.70±2.30 | 16.26±0.64 | | 16.13±0.63 | 16.33±0.64 |  |
| Lee et al., 2013 | Anantick stage | ST-3 | NK3 | 34.0466 | 75.9473 | 3506 | 3 | 2.7 | 0 | 0.97 | 45.52±1.40 | 07KNSTD |  | 12.92±0.69 | Ar. Mean: 13.55±0.88 ka | 13.27±0.75 | 14.00±1.00 | 14.30±1.10 | 12.70±1.00 | 12.70±1.00 | 14.00±2.00 | 14.40±1.90 | 13.80±1.80 | 12.90±0.40 | | 12.72±0.39 | 12.37±0.40 |  |
| Lee et al., 2013 | Anantick stage | ST-3 | NK4 | 34.0466 | 75.9473 | 3506 | 3 | 2.7 | 0 | 0.97 | 50.47±1.44 | 07KNSTD |  | 14.17±0.71 | Peak: 13.46 ka | 14.56±0.78 | 15.40±1.10 | 15.60±1.20 | 14.10±1.10 | 14.10±1.10 | 15.30±2.10 | 15.60±2.10 | 15.10±1.80 | 13.91±0.40 | | 13.81±0.40 | 13.72±0.40 |  |
| This study | YD/Early Holocene | Mk4 | S2 | 32.4 | 77.285 | 3522 | 2 | 2.7 | 0 | 0.913 | 36.64±1.92 | 07KNSTD | 12.18±0.99 | 11.96±0.86 | Chi-squared: 2.06 | 11.42±0.71 | 12.70±0.63 | 12.80±0.64 | 11.30±0.60 | 11.10±0.59 | 12.40±0.61 | 12.90±0.63 | 12.30±0.60 | 11.83±0.62 | | 11.52±0.62 | 10.92±0.58 |  |
|  |  |  |  |  |  |  |  |  |  |  |  |  |  |  | Skewness: -0.94 |  |  |  |  |  |  |  |  |  |  |  |  |  |
|  |  |  |  |  |  |  |  |  |  |  |  |  |  |  | Outlier: 0 |  |  |  |  |  |  |  |  |  |  |  |  |  |
| This study | YD/Early Holocene | Mk4 | S3 | 32.4 | 77.285 | 3527 | 2 | 2.7 | 0 | 0.915 | 39.64±1.69 | 07KNSTD |  | 12.9±0.85 | Wt. mean: 12.06±0.99 ka | 12.29±0.76 | 13.60±0.54 | 13.70±0.54 | 12.00±0.49 | 12.00±0.51 | 13.00±0.54 | 13.80±0.51 | 13.20±0.53 | 12.57±0.54 | | 12.23±0.54 | 11.76±0.50 |  |
| This study | YD/Early Holocene | Mk4 | S5 | 32.4 | 77.285 | 3526 | 2 | 2.7 | 0 | 0.915 | 32.65±1.65 | 07KNSTD |  | 10.86±0.65 | Ar. Mean: 12.18±0.99 ka | 10.33±0.63 | 11.30±0.57 | 11.50±0.56 | 10.00±0.54 | 9.87±0.50 | 11.10±0.56 | 11.60±0.53 | 11.00±0.56 | 10.96±0.56 | | 10.49±0.56 | 9.69±0.49 |  |
| This study | YD/Early Holocene | Mk4 | S6 | 32.4 | 77.285 | 3528 | 2 | 2.7 | 0 | 0.915 | 39.99±0.85 | 07KNSTD |  | 12.99±0.72 | Peak: ! | 12.40±0.63 | 14.00±0.27 | 13.80±0.27 | 12.24±0.25 | 12.00±0.26 | 13.00±0.27 | 13.90±0.26 | 13.30±0.27 | 12.65±0.27 | | 12.32±0.27 | 11.86±0.25 |  |
| Owen et al. (2001) | Kulti glacial stage | N/A | L20 | 32.6 | 76.9 | 2865 | 5 | 2.7 | 0 | 0.9 | 24.20±1.30 | LLNL3000 | 11.76±0.59 | 11.34±0.71 | Chi-squared: 0.54 | 12.1±0.89 | 12.80±1.20 | 12.90±1.30 | 11.20±1.10 | 11.00±1.10 | 13.00±2.00 | 13.00±2.00 | 12.70±1.80 | 11.93±0.64 | | 11.40±0.61 | 10.83±0.58 |  |
|  |  |  |  |  |  |  |  |  |  |  |  |  |  |  | Skewness: ! |  |  |  |  |  |  |  |  |  |  |  |  |  |
|  |  |  |  |  |  |  |  |  |  |  |  |  |  |  | Outlier: 0 |  |  |  |  |  |  |  |  |  |  |  |  |  |
|  |  |  |  |  |  |  |  |  |  |  |  |  |  |  | Wt. mean: 11.64±0.59 ka |  |  |  |  |  |  |  |  |  |  |  |  |  |
|  |  |  |  |  |  |  |  |  |  |  |  |  |  |  | Ar. Mean: 11.76±0.59 ka |  |  |  |  |  |  |  |  |  |  |  |  |  |
| Owen et al. (2001) | Kulti glacial stage | N/A | L21 | 32.6 | 76.9 | 2700 | 5 | 2.7 | 0 | 1 | 26.28±1.82 | LLNL3000 |  | 12.18±0.96 | Peak: 11.54 ka | 13.08±1.08 | 13.80±1.40 | 13.90±1.50 | 12.10±1.30 | 11.90±1.40 | 13.80±2.10 | 14.20±2.10 | 14.00±2.00 | 12.74±0.88 | | 12.11±0.84 | 11.69±0.81 |  |
| Scherler et al. (2010) | Tons Valley | location D | DS6-35 | 31.0789 | 78.4548 | 3642 | 2.5 | 2.7 | 0 | 0.98 | 1.67±0.11 | 07KNSTD | 11.09±0.50 ka | 0.53±0.05 | Chi-squared: ! | 0.50±0.04 | 0.69±0.08 | 0.72±0.06 | 0.57±0.08 | 0.57±0.08 | 0.64±0.11 | 0.63±0.11 | 0.65±0.11 | 0.6±0.04 | | 0.57±0.04 | 0.45±0.03 |  |
|  |  |  |  |  |  |  |  |  |  |  |  |  |  |  | Skewness: ! |  |  |  |  |  |  |  |  |  |  |  |  |  |
|  |  |  |  |  |  |  |  |  |  |  |  |  |  |  | Outlier: 1 |  |  |  |  |  |  |  |  |  |  |  |  |  |
|  |  |  |  |  |  |  |  |  |  |  |  |  |  |  | Wt. mean: 11.09±0.50 ka |  |  |  |  |  |  |  |  |  |  |  |  |  |
|  |  |  |  |  |  |  |  |  |  |  |  |  |  |  | Ar. Mean: 11.09±0.50 ka |  |  |  |  |  |  |  |  |  |  |  |  |  |
| Scherler et al. (2010) | Tons Valley | location D | DS6-37 | 31.0776 | 78.4564 | 3658 | 2.5 | 2.7 | 0 | 0.98 | 37.02±0.63 | 07KNSTD |  | 11.09±0.50 | Peak: ! | 10.55±0.46 | 11.56±0.89 | 12.14±0.89 | 10.19±0.89 | 10.19±0.89 | 11.20±1.70 | 11.80±1.60 | 11.10±1.60 | 11.±0.19 | | 10.78±0.18 | 9.92±0.17 |  |
| Saha et al., 2018 | Early Holocene | mH3 | HAMTAH-1401 | 32.3005 | 77.3684 | 3783 | 1.5 | 2.7 | 0 | 0.897 | 37.33±1.91 | 07KNSTD | 10.48±0.48 | 10.94±0.66 | Chi-squared: 0.48 | 10.48±0.62 | 11.40±1.10 | 11.60±1.10 | 10.00±1.00 | 10.02±0.96 | 11.10±1.70 | 11.60±1.70 | 11.00±1.60 | 9.83±0.51 | | 10.69±0.55 | 11.03±0.57 |  |
| Saha et al., 2018 | Early Holocene | mH3 | HAMTAH-1402 | 32.3006 | 77.3685 | 3769 | 2 | 2.7 | 0 | 0.887 | 19.86±1.69 | 07KNSTD |  | 6.24±0.51 | Skewness: +1.16 | 5.93±0.48 | 6.18±0.75 | 6.26±0.77 | 5.61±0.57 | 5.45±0.64 | 6.00±1.30 | 6.50±1.30 | 5.95±0.94 | 5.35±0.46 | | 6.00±0.51 | 6.33±0.54 |  |
| Saha et al., 2018 | Early Holocene | mH3 | HAMTAH-1403 | 32.2998 | 77.367 | 3808 | 1 | 2.7 | 0 | 0.879 | 58.05±2.35 | 07KNSTD |  | 16.18±1.03 | Outlier: 3 | 15.67±0.87 | 17.10±1.30 | 17.30±1.40 | 15.60±1.30 | 15.70±1.40 | 16.70±2.30 | 17.10±2.30 | 16.50±2.20 | 15.36±0.62 | | 15.24±0.62 | 15.36±0.62 |  |
| Saha et al., 2018 | Early Holocene | mH3 | HAMTAH-1404 | 32.2992 | 77.3662 | 3808 | 2 | 2.7 | 0 | 0.879 | 12.21±0.57 | 07KNSTD |  | 4.05±0.28 | Wt. mean: 10.42±0.48 ka | 3.79±0.23 | 3.87±0.38 | 3.90±0.40 | 3.60±0.30 | 3.31±0.31 | 3.70±0.73 | 4.12±0.73 | 3.72±0.54 | 3.25±0.15 | | 3.71±0.18 | 3.87±0.18 |  |
| Saha et al., 2018 | Early Holocene | mH3 | HAMTAH-1503 | 32.2942 | 77.3647 | 4112 | 3 | 2.7 | 0 | 0.898 | 41.47±0.86 | 07KNSTD |  | 10.36±0.53 | Ar. Mean: 10.48±0.48 ka | 10.02±0.47 | 10.76±0.89 | 10.91±0.97 | 9.57±0.86 | 9.50±0.80 | 10.30±1.60 | 10.90±1.60 | 10.20±1.50 | 9.33±0.19 | | 10.21±0.21 | 10.48±0.22 |  |
| Saha et al., 2018 | Early Holocene | mH3 | HAMTAH-1504 | 32.2954 | 77.3655 | 4083 | 3 | 2.7 | 0 | 0.879 | 38.89±0.73 | 07KNSTD |  | 10.13±0.54 | Peak: 10.37 ka | 9.77±0.47 | 10.46±0.86 | 10.61±0.94 | 9.28±0.82 | 9.23±0.77 | 10.00±1.60 | 10.60±1.60 | 9.90±1.40 | 9.07±0.17 | | 10.01±0.19 | 10.23±0.19 |  |
| Scherler et al. (2010) | Tons Valley | location F' | DS6-48 | 31.1458 | 78.4346 | 3544 | 2.5 | 2.7 | 0 | 0.97 | 34.43±0.55 | 07KNSTD | 10.26±0.35 ka | 10.51±0.46 | Chi-squared: ! | 11.08±0.5 | 11.56±0.88 | 11.67±0.96 | 10.15±0.88 | 10.15±0.88 | 11.20±1.70 | 11.80±1.60 | 11.20±1.60 | 11.12±0.18 | | 10.74±0.17 | 9.89±0.18 |  |
|  |  |  |  |  |  |  |  |  |  |  |  |  |  |  | Skewness: ! |  |  |  |  |  |  |  |  |  |  |  |  |  |
|  |  |  |  |  |  |  |  |  |  |  |  |  |  |  | Outlier: 0 |  |  |  |  |  |  |  |  |  |  |  |  |  |
|  |  |  |  |  |  |  |  |  |  |  |  |  |  |  | Wt. mean: 10.25±0.35 ka |  |  |  |  |  |  |  |  |  |  |  |  |  |
|  |  |  |  |  |  |  |  |  |  |  |  |  |  |  | Ar. Mean: 10.26±0.35 ka |  |  |  |  |  |  |  |  |  |  |  |  |  |
| Scherler et al. (2010) | Tons Valley | location F' | DS6-49 | 31.1461 | 78.4327 | 3514 | 2 | 2.7 | 0 | 0.97 | 32.17±0.59 | 07KNSTD |  | 10.01±0.45 | Peak: 10.25 ka | 10.61±0.51 | 10.94±0.92 | 11.05±0.97 | 9.56±0.92 | 9.56±0.92 | 10.60±1.70 | 11.20±1.70 | 10.60±1.50 | 10.70±0.20 | | 10.19±0.19 | 9.35±0.20 |  |
| Murari et al. (2014) | N/A | mk1 | KAL29 | 30.7301 | 79.0635 | 3648 | 2.5 | 2.7 | 0 | 0.948 | 35.72±3.21 | 07KNSTD | 10.25±0.83 | 10.66±0.92 | Chi-squared: 0.64 | 11.18±1.02 | 11.70±1.30 | 11.80±1.40 | 10.30±1.30 | 10.30±1.30 | 11.00±2.00 | 11.90±1.90 | 11.30±1.80 | 11.22±1.01 | | 10.88±0.98 | 10.04±1.01 |  |
| Murari et al. (2014) | N/A | mk1 | KAL30 | 30.7296 | 79.0635 | 3650 | 2.5 | 2.7 | 0 | 0.948 | 57.92±9.85 | 07KNSTD |  | 16.47±2.63 | Skewness: -1.12 | 17.29±2.7 | 18.10±2.90 | 18.30±2.90 | 16.50±2.90 | 16.50±2.90 | 17.60±3.60 | 18.10±3.50 | 17.50±3.40 | 16.43±2.81 | | 16.06±2.74 | 16.29±2.81 |  |
| Murari et al. (2014) | N/A | mk1 | KAL31 | 30.7295 | 79.0635 | 3642 | 2.5 | 2.7 | 0 | 0.944 | 36.63±8.06 | 07KNSTD |  | 10.95±2.3 | Outlier: 1 | 11.46±2.34 | 12.00±2.60 | 12.20±2.60 | 10.70±2.60 | 10.70±2.60 | 12.00±3.00 | 12.20±2.90 | 11.60±2.90 | 11.44±2.52 | | 11.13±2.46 | 10.37±2.52 |  |
| Murari et al. (2014) | N/A | mk1 | KAL32 | 30.7287 | 79.0635 | 3641 | 2.5 | 2.7 | 0 | 0.94 | 29.59±3.85 | 07KNSTD |  | 8.98±1.25 | Wt. mean: 10.29±0.83 ka | 9.67±1.33 | 9.80±1.60 | 9.90±1.60 | 8.50±1.60 | 8.50±1.60 | 9.00±2.00 | 10.00±2.00 | 9.40±1.90 | 9.84±1.28 | | 9.32±1.21 | 8.42±1.28 |  |
| Murari et al. (2014) | N/A | mk1 | KAL33 | 30.7295 | 79.0635 | 3643 | 2 | 2.7 | 0 | 0.944 | 32.61±1.63 | 07KNSTD |  | 9.85±0.64 | Ar. Mean: 10.25±0.83 ka | 10.43±0.66 | 11.00±1.00 | 10.80±1.10 | 9.37±1.00 | 9.37±1.00 | 10.30±1.70 | 11.00±1.70 | 10.30±1.50 | 10.48±0.53 | | 10.08±0.51 | 9.18±0.53 |  |
| Murari et al. (2014) | N/A | mk1 | KAL34 | 30.7244 | 79.0652 | 3605 | 3 | 2.7 | 0 | 0.958 | 35.71±1.79 | 07KNSTD |  | 10.82±0.62 | Peak: 10.38 ka | 11.33±0.7 | 12.00±1.00 | 12.00±1.10 | 10.00±1.00 | 10.00±1.00 | 11.50±1.80 | 12.10±1.70 | 11.50±1.60 | 11.34±0.57 | | 11.01±0.55 | 10.21±0.57 |  |
| Owen et al. (2010) | N/A | M7 | Na64 | 30.4704 | 81.2058 | 5195 | 2 | 2.7 | 0 | 0.98 | 26.50±0.66 | 07KNSTD | 8.75±0.55 | 4.29±0.25 | Chi-squared: ! | 4.06±0.20 | 3.98±0.11 | 4.07±0.11 | 3.81±0.08 | 3.50±0.09 | 3.62±0.09 | 3.98±0.11 | 3.60±0.09 | 4.11±0.10 | | 4.06±0.10 | 3.42±0.09 |  |
|  |  |  |  |  |  |  |  |  |  |  |  |  |  |  | Skewness: ! |  |  |  |  |  |  |  |  |  |  |  |  |  |
|  |  |  |  |  |  |  |  |  |  |  |  |  |  |  | Outlier: 2 |  |  |  |  |  |  |  |  |  |  |  |  |  |
| Owen et al. (2010) | N/A | M7 | Na66 | 30.4711 | 81.2056 | 5159 | 5 | 2.7 | 0 | 0.978 | 82.78±2.37 | 07KNSTD |  | 12.00±0.74 | Wt. mean: 8.71±0.55 ka | 11.72±0.60 | 12.52±0.34 | 12.70±0.35 | 11.55±0.33 | 11.35±0.33 | 11.60±0.32 | 12.20±0.30 | 11.40±0.32 | 11.82±0.34 | | 11.91±0.34 | 11.19±0.32 |  |
| Owen et al. (2010) | N/A | M7 | Na67 | 30.4712 | 81.2056 | 5154 | 2 | 2.7 | 0 | 0.978 | 57.37±2.22 | 07KNSTD |  | 8.36±0.60 | Ar. Mean: 8.75±0.55 ka | 8.12±0.48 | 8.41±0.35 | 8.57±0.35 | 7.68±0.29 | 7.70±0.30 | 7.80±0.29 | 8.40±0.31 | 8.00±0.28 | 8.57±0.33 | | 8.41±0.33 | 7.57±0.29 |  |
| Owen et al. (2010) | N/A | M7 | Na68 | 30.4712 | 81.2056 | 5154 | 1 | 2.7 | 0 | 0.978 | 62.51±1.84 | 07KNSTD |  | 9.14±0.66 | Peak: 8.68 ka | 8.77±0.54 | 9.14±0.29 | 9.30±0.30 | 8.31±0.26 | 8.33±0.25 | 8.40±0.25 | 9.00±0.27 | 8.30±0.25 | 9.42±0.28 | | 9.25±0.28 | 8.18±0.24 |  |
| Barnard et al. (2004a) | Kedar glacial stage | N/A | BH19 | 30.9436 | 78.952 | 4323 | 5 | 2.7 | 0 | 0.97 | 47.24±1.13 | LLNL3000 | 8.28±0.45 | 8.59±0.48 | Chi-squared: ! | 9.06±0.63 | 9.17±0.81 | 9.32±0.88 | 8.20±0.81 | 8.20±0.81 | 8.70±1.40 | 9.30±1.50 | 8.60±1.30 | 9.32±0.22 | | 8.92±0.21 | 8.07±0.22 |  |
|  |  |  |  |  |  |  |  |  |  |  |  |  |  |  | Skewness: ! |  |  |  |  |  |  |  |  |  |  |  |  |  |
|  |  |  |  |  |  |  |  |  |  |  |  |  |  |  | Outlier: 0 |  |  |  |  |  |  |  |  |  |  |  |  |  |
|  |  |  |  |  |  |  |  |  |  |  |  |  |  |  | Wt. mean: 8.22±0.45 ka |  |  |  |  |  |  |  |  |  |  |  |  |  |
|  |  |  |  |  |  |  |  |  |  |  |  |  |  |  | Ar. Mean: 8.28±0.45 ka |  |  |  |  |  |  |  |  |  |  |  |  |  |
| Barnard et al. (2004a) | Kedar glacial stage | N/A | BH20 | 30.9442 | 78.9505 | 4242 | 5 | 2.7 | 0 | 0.97 | 42.00±1.00 | LLNL3000 |  | 7.96±0.4 | Peak: 8.15 ka | 8.31±0.52 | 8.46±0.71 | 8.60±0.80 | 7.54±0.71 | 7.54±0.71 | 8.10±1.20 | 8.70±1.30 | 8.00±1.10 | 8.49±0.20 | | 8.20±0.20 | 7.46±0.20 |  |
| Scherler et al. (2010) | Tons Valley | Location F | DS6-54/64 | 31.1487 | 78.4268 | 3504 | 2.5 | 2.7 | 0 | 0.98 | 20.22±0.52 | 07KNSTD | 6.09±0.54 | 6.41±0.29 | Chi-squared: 4.00 | 6.81±0.35 | 6.80±0.60 | 6.92±0.65 | 6.08±0.60 | 6.08±0.60 | 6.70±1.10 | 7.23±0.98 | 6.62±0.97 | 6.90±0.18 | | 6.54±0.17 | 5.87±0.18 |  |
|  |  |  |  |  |  |  |  |  |  |  |  |  |  |  | Skewness: +0.21 |  |  |  |  |  |  |  |  |  |  |  |  |  |
|  |  |  |  |  |  |  |  |  |  |  |  |  |  |  | Outlier: 0 |  |  |  |  |  |  |  |  |  |  |  |  |  |
| Scherler et al. (2010) | Tons Valley | Location F | DS6-61 | 31.1459 | 78.4278 | 3412 | 2 | 2.7 | 0 | 0.95 | 19.69±0.47 | 07KNSTD |  | 6.68±0.3 | Wt. mean: 6.00±0.54 ka | 7.12±0.36 | 7.22±0.54 | 7.28±0.59 | 6.35±0.54 | 6.35±0.54 | 7.10±1.10 | 8.00±1.00 | 7.00±1.00 | 7.20±0.17 | | 6.82±0.16 | 6.17±0.17 |  |
| Scherler et al. (2010) | Tons Valley | Location F | DS6-63 | 31.1493 | 78.4279 | 3516 | 2.5 | 2.7 | 0 | 0.97 | 17.36±0.44 | 07KNSTD |  | 5.64±0.25 | Ar. Mean: 6.09±0.54 ka | 6.03±0.3 | 5.87±0.45 | 5.93±0.49 | 5.30±0.45 | 5.30±0.45 | 5.73±0.76 | 6.10±1.10 | 5.71±0.66 | 6.05±0.15 | | 5.70±0.14 | 5.06±0.15 |  |
| Scherler et al. (2010) | Tons Valley | Location F | DS05-05B | 31.1489 | 78.4272 | 3495 | 2.5 | 2.7 | 0 | 0.98 | 17.28±0.43 | 07KNSTD |  | 5.63±0.24 | Peak: ! | 6.02±0.3 | 5.86±0.43 | 5.92±0.48 | 5.30±0.43 | 5.30±0.43 | 5.72±0.75 | 6.10±1.10 | 5.70±0.66 | 6.03±0.15 | | 5.68±0.14 | 5.04±0.15 |  |
| Srivastava (2012; Sci. report) | Shivling glacial stage | N/A | MILAP4 | 30.9964 | 78.9308 | 3029 | 5 | 2.7 | 0 | 0.918 | 10.70±0.38 | 07KNSTD | 5.22±0.27 | 5.48±0.28 | Chi-squared: 0.63 | 5.04±0.28 | 5.33±0.35 | 5.36±0.39 | 4.73±0.37 | 4.50±0.40 | 5.25±0.68 | 5.54±0.58 | 5.20±0.60 | 5.41±0.19 | | 5.04±0.19 | 4.43±0.16 |  |
| Srivastava (2012; Sci. report) | Shivling glacial stage | N/A | MILAP5 | 30.9971 | 78.9274 | 3012 | 5 | 2.7 | 0 | 0.907 | 9.66±0.31 | 07KNSTD |  | 5.13±0.29 | Skewness: -0.86 | 4.68±0.29 | 5.00±0.38 | 5.02±0.38 | 4.39±0.34 | 4.17±0.36 | 4.91±0.71 | 5.24±0.61 | 4.91±0.63 | 5.02±0.16 | | 4.60±0.16 | 4.09±0.13 |  |
| Srivastava (2012; Sci. report) | Shivling glacial stage | N/A | MILAP8 | 30.9971 | 78.9311 | 3021 | 2 | 2.7 | 0 | 0.899 | 10.68±0.61 | 07KNSTD |  | 5.48±0.35 | Outlier: 0 | 5.04±0.35 | 5.30±0.40 | 5.36±0.43 | 4.72±0.42 | 4.51±0.45 | 5.25±0.71 | 5.54±0.61 | 5.24±0.63 | 5.41±0.31 | | 5.03±0.31 | 4.42±0.26 |  |
| Srivastava (2012; Sci. report) | Shivling glacial stage | N/A | MIALP10 | 30.9968 | 78.9289 | 3017 | 2 | 2.7 | 0 | 0.911 | 10.50±0.62 | 07KNSTD |  | 5.35±0.35 | Wt. mean: 5.23±0.27 ka | 4.89±0.35 | 5.20±0.40 | 5.24±0.42 | 4.60±0.42 | 4.39±0.44 | 5.13±0.71 | 5.44±0.62 | 5.12±0.64 | 5.28±0.31 | | 4.87±0.31 | 4.30±0.25 |  |
| Srivastava (2012; Sci. report) | Shivling glacial stage | N/A | MILAP11 | 30.9967 | 78.9284 | 3022 | 2 | 2.7 | 0 | 0.907 | 10.44±0.58 | 07KNSTD |  | 5.33±0.35 | Ar. Mean: 5.22±0.27 ka | 4.89±0.35 | 5.20±0.41 | 5.22±0.41 | 4.59±0.41 | 4.37±0.43 | 5.11±0.71 | 5.42±0.62 | 5.11±0.63 | 5.26±0.29 | | 4.85±0.29 | 4.29±0.24 |  |
| Srivastava (2012; Sci. report) | Shivling glacial stage | N/A | MILAP12 | 30.9968 | 78.9277 | 3017 | 2 | 2.7 | 0 | 0.905 | 9.68±0.43 | 07KNSTD |  | 5.02±0.32 | Peak: 5.27 ka | 4.57±0.32 | 4.90±0.40 | 4.92±0.41 | 4.30±0.36 | 4.07±0.38 | 4.80±0.73 | 5.15±0.64 | 4.80±0.66 | 4.88±0.22 | | 4.49±0.22 | 3.99±0.18 |  |
| Srivastava (2012; Sci. report) | Shivling glacial stage | N/A | MILAP13 | 30.9969 | 78.9272 | 3039 | 2 | 2.7 | 0 | 0.907 | 9.25±0.57 | 07KNSTD |  | 4.76±0.36 |  | 4.33±0.36 | 4.62±0.46 | 4.65±0.48 | 4.07±0.36 | 3.83±0.39 | 4.52±0.76 | 4.90±0.73 | 4.50±0.70 | 4.56±0.28 | | 4.25±0.28 | 3.76±0.23 |  |
| Owen et al. (2010) | N/A | M8 | Na58 | 30.4683 | 81.2064 | 5325 | 5 | 2.7 | 0 | 1 | 38.41±1.64 | 07KNSTD | 5.01±0.88 | 5.63±0.30 | Chi-squared: ! | 5.41±0.28 | 5.38±0.17 | 5.45±0.17 | 5.04±0.19 | 4.78±0.20 | 4.97±0.18 | 5.34±0.16 | 4.93±0.18 | 5.62±0.24 | | 5.54±0.24 | 4.71±0.20 |  |
|  |  |  |  |  |  |  |  |  |  |  |  |  |  |  | Skewness: ! |  |  |  |  |  |  |  |  |  |  |  |  |  |
|  |  |  |  |  |  |  |  |  |  |  |  |  |  |  | Outlier: 0 |  |  |  |  |  |  |  |  |  |  |  |  |  |
|  |  |  |  |  |  |  |  |  |  |  |  |  |  |  | Wt. mean: 4.92±0.88 ka |  |  |  |  |  |  |  |  |  |  |  |  |  |
|  |  |  |  |  |  |  |  |  |  |  |  |  |  |  | Ar. Mean: 5.01±0.88 ka |  |  |  |  |  |  |  |  |  |  |  |  |  |
| Owen et al. (2010) | N/A | M8 | Na59 | 30.4683 | 81.205 | 5325 | 1 | 2.7 | 0 | 1 | 29.51±0.83 | 07KNSTD |  | 4.39±0.26 | Peak: ! | 4.16±0.21 | 4.08±0.12 | 4.17±0.13 | 3.89±0.09 | 3.56±0.10 | 3.70±0.10 | 4.05±0.13 | 3.66±0.11 | 4.20±0.12 | | 4.15±0.12 | 3.50±0.10 |  |
| Srivastava (2012; Sci. report) | Gangotri glacial stage | N/A | MILAP2 | 30.7633 | 79.0775 | 4343 | 5 | 2.7 | 0 | 0.98 | 9.83±0.29 | 07KNSTD | 2.16±0.35 | 2.41±0.18 | Chi-squared: ! | 2.22±0.18 | 2.43±0.19 | 2.50±0.20 | 2.20±0.20 | 1.94±0.17 | 2.28±0.36 | 2.52±0.33 | 2.29±0.32 | 2.26±0.07 | | 2.17±0.07 | 1.91±0.06 |  |
|  |  |  |  |  |  |  |  |  |  |  |  |  |  |  | Skewness: ! |  |  |  |  |  |  |  |  |  |  |  |  |  |
|  |  |  |  |  |  |  |  |  |  |  |  |  |  |  | Outlier: 0 |  |  |  |  |  |  |  |  |  |  |  |  |  |
|  |  |  |  |  |  |  |  |  |  |  |  |  |  |  | Wt. mean: 2.08±0.35 ka |  |  |  |  |  |  |  |  |  |  |  |  |  |
|  |  |  |  |  |  |  |  |  |  |  |  |  |  |  | Ar. Mean: 2.16±0.35 ka |  |  |  |  |  |  |  |  |  |  |  |  |  |
| Srivastava (2012; Sci. report) | Gangotri glacial stage | N/A | MILAP3 | 30.9129 | 79.0783 | 4335 | 5 | 2.7 | 0 | 0.98 | 7.98±0.26 | 07KNSTD |  | 1.91±0.13 | Peak: ! | 1.80±0.13 | 1.97±0.18 | 2.00±0.18 | 1.76±0.15 | 1.58±0.14 | 1.84±0.29 | 2.06±0.35 | 1.85±0.27 | 1.76±0.06 | | 1.72±0.06 | 1.55±0.05 |  |
| Scherler et al. (2010) | Tons Valley | Location G | DS6-57 | 31.1418 | 78.4536 | 3636 | 2.5 | 2.7 | 0 | 0.95 | 1.37±0.05 | 07KNSTD | 0.66±0.34 | 0.42±0.03 | Chi-squared: ! | 0.44±0.03 | 0.58±0.05 | 0.59±0.06 | 0.49±0.05 | 0.49±0.05 | 0.54±0.08 | 0.53±0.08 | 0.55±0.08 | 0.51±0.02 | | 0.49±0.02 | 0.38±0.02 |  |
|  |  |  |  |  |  |  |  |  |  |  |  |  |  |  | Skewness: ! |  |  |  |  |  |  |  |  |  |  |  |  |  |
|  |  |  |  |  |  |  |  |  |  |  |  |  |  |  | Outlier: 0 |  |  |  |  |  |  |  |  |  |  |  |  |  |
|  |  |  |  |  |  |  |  |  |  |  |  |  |  |  | Wt. mean: 0.55±0.34 ka |  |  |  |  |  |  |  |  |  |  |  |  |  |
|  |  |  |  |  |  |  |  |  |  |  |  |  |  |  | Ar. Mean: 0.66±0.34 ka |  |  |  |  |  |  |  |  |  |  |  |  |  |
| Scherler et al. (2010) | Tons Valley | Location G | DS6-58 | 31.1418 | 78.4531 | 3623 | 2.5 | 2.7 | 0 | 0.94 | 2.82±0.08 | 07KNSTD |  | 0.9±0.05 | Peak: ! | 0.96±0.07 | 1.10±0.07 | 1.11±0.06 | 0.98±0.07 | 0.98±0.07 | 1.06±0.12 | 1.09±0.14 | 1.07±0.11 | 0.99±0.03 | | 0.95±0.03 | 0.80±0.03 |  |
| Saha et al. (2015) | N/A | N/A | ZK73 | 32.7927 | 77.4293 | 4757 | 5 | 2.7 | 0 | 1 | 3.10±0.50 | 07KNSTD | 0.62±0.15 | 0.51±0.09 | Chi-squared: 2.13 | 0.52±0.09 | 0.68±0.14 | 0.72±0.11 | 0.59±0.10 | 0.47±0.08 | 0.62±0.12 | 0.61±0.12 | 0.62±0.13 | 0.60±0.10 | | 0.58±0.09 | 0.46±0.07 |  |
|  |  |  |  |  |  |  |  |  |  |  |  |  |  |  | Skewness: ! |  |  |  |  |  |  |  |  |  |  |  |  |  |
|  |  |  |  |  |  |  |  |  |  |  |  |  |  |  | Outlier: 1 |  |  |  |  |  |  |  |  |  |  |  |  |  |
|  |  |  |  |  |  |  |  |  |  |  |  |  |  |  | Wt. mean: 0.59±0.15 ka |  |  |  |  |  |  |  |  |  |  |  |  |  |
| Saha et al. (2015) | N/A | N/A | ZK74 | 32.7929 | 77.4292 | 4764 | 5 | 2.7 | 0 | 1 | 4.40±0.70 | 07KNSTD |  | 0.72±0.12 | Ar. Mean: 0.62±0.15 ka | 0.75±0.13 | 0.93±0.12 | 0.97±0.13 | 0.82±0.13 | 0.66±0.12 | 0.86±0.20 | 0.87±0.20 | 0.87±0.18 | 0.83±0.13 | | 0.81±0.13 | 0.65±0.10 |  |
| Saha et al. (2015) | N/A | N/A | ZK75 | 32.7929 | 77.4292 | 4749 | 5 | 2.7 | 0 | 1 | 159.70±7.00 | 07KNSTD |  | 23.16±1.31 | Peak: 0.54 ka | 22.67±1.3 | 23.60±1.80 | 24.80±1.80 | 23.10±1.90 | 24.30±2.20 | 22.90±3.20 | 23.00±3.20 | 22.50±2.90 | 21.82±0.96 | | 22.56±1.00 | 23.84±1.05 |  |
| Barnard et al. (2004b) | N/A | Moraine m4 | NDL2 | 30.45 | 80.12 | 3534 | 5 | 2.7 | 0 | 0.97 | 1.40±0.50 | LLNL3000 | 0.60±0.28 | 0.4±0.15 | Chi-squared: ! | 0.43±0.16 | 0.56±0.24 | 0.56±0.25 | 0.47±0.24 | 0.47±0.24 | 0.52±0.22 | 0.51±0.21 | 0.53±0.23 | ± | | ± | ± |  |
|  |  |  |  |  |  |  |  |  |  |  |  |  |  |  | Skewness: ! |  |  |  |  |  |  |  |  |  |  |  |  |  |
|  |  |  |  |  |  |  |  |  |  |  |  |  |  |  | Outlier: 0 |  |  |  |  |  |  |  |  |  |  |  |  |  |
|  |  |  |  |  |  |  |  |  |  |  |  |  |  |  | Wt. mean: 0.62±0.28 ka |  |  |  |  |  |  |  |  |  |  |  |  |  |
|  |  |  |  |  |  |  |  |  |  |  |  |  |  |  | Ar. Mean: 0.60±0.28 ka |  |  |  |  |  |  |  |  |  |  |  |  |  |
| Barnard et al. (2004b) | N/A | Moraine m4 | NDL4 | 30.45 | 80.12 | 3522 | 5 | 2.7 | 0 | 0.96 | 2.70±0.40 | LLNL3000 |  | 0.79±0.13 | Peak: ! | 0.86±0.14 | 1.01±0.14 | 1.02±0.14 | 0.89±0.14 | 0.89±0.14 | 0.97±0.18 | 0.99±0.21 | 0.99±0.16 | 0.92±0.14 | | 0.87±0.13 | 0.71±0.14 |  |
| Barnard et al. (2004a) | Gangotri glacial stage | N/A | BH29 | 30.945 | 79.0615 | 3973 | 5 | 2.7 | 0 | 0.97 | 3.12±0.84 | LLNL3000 | 0.56±0.30 | 0.71±0.2 | Chi-squared: 4.45 | 0.75±0.21 | 0.93±0.21 | 0.94±0.21 | 0.80±0.21 | 0.80±0.21 | 0.87±0.29 | 0.87±0.31 | 0.89±0.28 | 0.82±0.22 | | 0.79±0.21 | 0.63±0.22 |  |
|  |  |  |  |  |  |  |  |  |  |  |  |  |  |  | Skewness: +0.86 |  |  |  |  |  |  |  |  |  |  |  |  |  |
| Barnard et al. (2004a) | Gangotri glacial stage | N/A | BH30 | 30.9449 | 79.0616 | 3956 | 5 | 2.7 | 0 | 0.97 | 1.53±0.67 | LLNL3000 |  | 0.34±0.16 | Outlier: 0 | 0.37±0.16 | 0.48±0.26 | 0.49±0.22 | 0.41±0.26 | 0.41±0.26 | 0.44±0.24 | 0.43±0.23 | 0.45±0.24 | 0.43±0.19 | | 0.41±0.18 | 0.31±0.19 |  |
| Barnard et al. (2004a) | Gangotri glacial stage | N/A | BH31 | 30.9448 | 79.0616 | 3973 | 5 | 2.7 | 0 | 0.97 | 1.12±0.68 | LLNL3000 |  | 0.26±0.16 | Wt. mean: 0.49±0.30 ka | 0.24±0.15 | 0.35±0.26 | 0.35±0.27 | 0.31±0.26 | 0.31±0.26 | 0.32±0.23 | 0.31±0.23 | 0.32±0.23 | 1.07±0.17 | | 1.04±0.17 | 0.90±0.17 |  |
| Barnard et al. (2004a) | Gangotri glacial stage | N/A | BH32 | 30.9448 | 79.0619 | 3956 | 5 | 2.7 | 0 | 0.97 | 4.37±0.70 | LLNL3000 |  | 1.01±0.17 | Ar. Mean: 0.56±0.30 ka | 1.07±0.19 | 1.20±0.17 | 1.21±0.21 | 1.08±0.17 | 1.08±0.17 | 1.14±0.22 | 1.18±0.24 | 1.15±0.21 | 0.51±0.02 | | 0.49±0.02 | 0.38±0.02 |  |
| Srivastava (2012; Sci. report) | Gangotri glacial stage | N/A | Milap7 | 30.9454 | 79.06 | 3935 | 2 | 2.7 | 0 | 0.949 | 1.69±0.07 | 07KNSTD |  | 0.47±0.04 | Peak: ! | 0.44±0.04 | 0.61±0.06 | 0.62±0.06 | 0.52±0.04 | 0.41±0.04 | 0.57±0.09 | 0.55±0.09 | 0.58±0.08 | 0.54±0.02 | | 0.52±0.02 | 0.40±0.02 |  |
| Lee et al., 2014 | Lonp stage | TG-3 | NK29 | 34.0559 | 75.9181 | 3679 | 2.5 | 2.7 | 0 | 0.957 | 1.56±0.05 | 07KNSTD | 0.53±0.13 | 0.43±0.03 | Chi-squared: 12.53 | 0.44±0.03 | 0.57±0.04 | 0.57±0.05 | 0.50±0.04 | 0.40±0.04 | 0.54±0.08 | 0.54±0.08 | 0.55±0.08 | 0.51±0.02 | | 0.49±0.02 | 0.39±0.01 |  |
|  |  |  |  |  |  |  |  |  |  |  |  |  |  |  | Skewness: ! |  |  |  |  |  |  |  |  |  |  |  |  |  |
|  |  |  |  |  |  |  |  |  |  |  |  |  |  |  | Outlier: 0 |  |  |  |  |  |  |  |  |  |  |  |  |  |
|  |  |  |  |  |  |  |  |  |  |  |  |  |  |  | Wt. mean: 0.47±0.13 ka |  |  |  |  |  |  |  |  |  |  |  |  |  |
|  |  |  |  |  |  |  |  |  |  |  |  |  |  |  | Ar. Mean: 0.53±0.13 ka |  |  |  |  |  |  |  |  |  |  |  |  |  |
| Lee et al., 2014 | Lonp stage | TG-3 | NK30 | 34.0548 | 75.9173 | 3720 | 4 | 2.7 | 0 | 0.957 | 2.26±0.18 | 07KNSTD |  | 0.62±0.06 | Peak: ! | 0.65±0.07 | 0.80±0.08 | 0.81±0.07 | 0.71±0.08 | 0.57±0.06 | 0.77±0.14 | 0.77±0.14 | 0.78±0.11 | 0.72±0.06 | | 0.68±0.05 | 0.56±0.04 |  |
| This study | LIA | Mk2 | S9 | 32.4233 | 77.3067 | 3676 | 2 | 2.7 | 0 | 0.917 | 1.27±0.46 | 07KNSTD | 0.51±0.16 | 0.40±0.15 | Chi-squared: ! | 0.37±0.14 | 0.52±0.19 | 0.53±0.19 | 0.45±0.14 | 0.35±0.13 | 0.49±0.18 | 0.48±0.17 | 0.50±0.18 | 0.47±0.17 | | 0.44±0.17 | 0.35±0.13 |  |
|  |  |  |  |  |  |  |  |  |  |  |  |  |  |  | Skewness: ! |  |  |  |  |  |  |  |  |  |  |  |  |  |
|  |  |  |  |  |  |  |  |  |  |  |  |  |  |  | Outlier: 0 |  |  |  |  |  |  |  |  |  |  |  |  |  |
|  |  |  |  |  |  |  |  |  |  |  |  |  |  |  | Wt. mean: 0.57±0.16 ka |  |  |  |  |  |  |  |  |  |  |  |  |  |
|  |  |  |  |  |  |  |  |  |  |  |  |  |  |  | Ar. Mean: 0.51±0.16 ka |  |  |  |  |  |  |  |  |  |  |  |  |  |
| This study | LIA | Mk2 | S10 | 32.4233 | 77.3067 | 3678 | 2 | 2.7 | 0 | 0.913 | 1.95±0.19 | 07KNSTD |  | 0.62±0.08 | Peak: 0.61 ka | 0.59±0.07 | 0.79±0.07 | 0.80±0.06 | 0.68±0.07 | 0.54±0.05 | 0.75±0.07 | 0.74±0.07 | 0.76±0.07 | 0.70±0.07 | | 0.66±0.07 | 0.53±0.05 |  |
| Owen et al. (2010) | N/A | M9 | Na54 | 30.4637 | 81.2157 | 5503 | 1 | 2.7 | 0 | 0.947 | 2.98±0.39 | 07KNSTD | 0.46±0.10 | 0.38±0.06 | Chi-squared: 2.57 | 0.39±0.06 | 0.54±0.08 | 0.55±0.08 | 0.47±0.08 | 0.47±0.08 | 0.47±0.10 | 0.45±0.09 | 0.48±0.10 | 0.47±0.06 | | 0.47±0.06 | 0.35±0.06 |  |
|  |  |  |  |  |  |  |  |  |  |  |  |  |  |  | Skewness: +1.65 |  |  |  |  |  |  |  |  |  |  |  |  |  |
|  |  |  |  |  |  |  |  |  |  |  |  |  |  |  | Outlier: 0 |  |  |  |  |  |  |  |  |  |  |  |  |  |
| Owen et al. (2010) | N/A | M9 | Na55 | 30.4635 | 81.2157 | 5520 | 1.5 | 2.7 | 0 | 0.947 | 3.09±0.55 | 07KNSTD |  | 0.39±0.08 | Wt. mean: 0.46±0.10 ka | 0.41±0.08 | 0.55±0.10 | 0.57±0.12 | 0.48±0.10 | 0.48±0.10 | 0.48±0.12 | 0.46±0.11 | 0.49±0.11 | 0.49±0.09 | | 0.49±0.09 | 0.36±0.09 |  |
| Owen et al. (2010) | N/A | M9 | Na56 | 30.4629 | 81.2157 | 5509 | 2 | 2.7 | 0 | 0.928 | 4.09±0.36 | 07KNSTD |  | 0.54±0.06 | Ar. Mean: 0.46±0.10 ka | 0.57±0.06 | 0.75±0.07 | 0.76±0.08 | 0.65±0.07 | 0.65±0.07 | 0.66±0.13 | 0.62±0.11 | 0.67±0.12 | 0.65±0.06 | | 0.65±0.06 | 0.49±0.06 |  |
| Owen et al. (2010) | N/A | M9 | Na57 | 30.4632 | 81.2155 | 5508 | 1 | 2.7 | 0 | 0.933 | 6.69±0.86 | 07KNSTD |  | 0.89±0.13 | Peak: ! | 0.93±0.14 | 1.08±0.12 | 1.10±0.10 | 1.00±0.12 | 1.00±0.12 | 1.00±0.15 | 1.00±0.18 | 1.00±0.14 | 0.98±0.13 | | 0.98±0.13 | 0.79±0.13 |  |
| Murari et al. (2014) | mk2 | N/A | KAL35 | 30.7448 | 79.6503 | 3841 | 4 | 2.7 | 0 | 0.953 | 1.81±0.41 | 07KNSTD | 0.31±0.17 | 0.51±0.13 | Chi-squared: 19.33 | 0.54±0.13 | 0.71±0.13 | 0.72±0.14 | 0.59±0.13 | 0.59±0.13 | 0.65±0.19 | 0.64±0.18 | 0.67±0.19 | 0.61±0.14 | | 0.59±0.13 | 0.46±0.14 |  |
|  |  |  |  |  |  |  |  |  |  |  |  |  |  |  | Skewness: +1.73 |  |  |  |  |  |  |  |  |  |  |  |  |  |
| Murari et al. (2014) | mk2 | N/A | KAL36 | 30.7454 | 79.0652 | 3853 | 5 | 2.7 | 0 | 0.95 | 0.80±0.09 | 07KNSTD |  | 0.22±0.03 | Outlier: 2 | 0.24±0.03 | 0.31±0.04 | 0.32±0.05 | 0.28±0.04 | 0.28±0.04 | 0.29±0.06 | 0.28±0.06 | 0.29±0.05 | ± | | 0.27±0.03 | 0.21±0.00 |  |
| Murari et al. (2014) | mk2 | N/A | KAL37 | 30.7462 | 79.0655 | 3884 | 3 | 2.7 | 0 | 0.959 | 72.22±22.22 | 07KNSTD |  | 17.93±4.86 | Wt. mean: 0.22±0.17 ka | 18.64±4.86 | 19.40±4.40 | 19.60±4.50 | 17.90±4.40 | 17.90±4.40 | 18.90±5.40 | 19.30±4.90 | 18.70±5.20 | 17.75±5.49 | | 17.45±5.49 | 17.83±5.51 |  |
| Murari et al. (2014) | mk2 | N/A | KAL38 | 30.7473 | 79.0661 | 3909 | 5 | 2.7 | 0 | 0.959 | 0.80±0.08 | 07KNSTD |  | 0.21±0.02 | Ar. Mean: 0.31±0.17 ka | 0.23±0.03 | 0.31±0.03 | 0.31±0.03 | 0.27±0.03 | 0.27±0.03 | 0.28±0.05 | 0.27±0.05 | 0.28±0.05 | ± | | 0.26±0.03 | 0.20±0.00 |  |
| Murari et al. (2014) | mk2 | N/A | KAL39 | 30.7478 | 79.0662 | 3915 | 2.5 | 2.7 | 0 | 0.096 | 1.22±0.22 | 07KNSTD |  | 3.47±0.61 | Peak: ! | 3.75±0.67 | 3.50±0.65 | 3.55±0.72 | 3.30±0.65 | 3.30±0.65 | 3.35±0.78 | 3.66±0.86 | 3.35±0.75 | 3.29±0.59 | | 3.17±0.57 | 2.79±0.59 |  |
| Saha et al., 2018 | Late Holocene | mH1a | HAMTAH-1405 | 32.2725 | 77.3574 | 4014 | 2 | 2.7 | 0 | 0.943 | 0.89±0.19 | 07KNSTD | 0.26±0.13 | 0.23±0.05 | Chi-squared: 8.55 | 0.21±0.05 | 0.30±0.06 | 0.31±0.05 | 0.27±0.06 | 0.20±0.05 | 0.28±0.08 | 0.27±0.08 | 0.28±0.08 | ± | | 0.26±0.00 | 0.20±0.04 |  |
|  |  |  |  |  |  |  |  |  |  |  |  |  |  |  | Skewness: +0.87 |  |  |  |  |  |  |  |  |  |  |  |  |  |
| Saha et al., 2018 | Late Holocene | mH1a | HAMTAH-1406 | 32.2722 | 77.3575 | 4023 | 1 | 2.7 | 0 | 0.944 | 1.56±0.41 | 07KNSTD |  | 0.40±0.11 | Outlier: 2 | 0.37±0.10 | 0.52±0.13 | 0.52±0.14 | 0.45±0.11 | 0.35±0.10 | 0.48±0.15 | 0.47±0.15 | 0.49±0.14 | 0.34±0.09 | | 0.44±0.12 | 0.46±0.12 |  |
| Saha et al., 2018 | Late Holocene | mH1a | HAMTAH-1408 | 32.2686 | 77.3585 | 4111 | 2.5 | 2.7 | 0 | 0.954 | 9.06±0.55 | 07KNSTD |  | 2.35±0.22 | Wt. mean: 0.18±0.13 ka | 2.21±0.18 | 2.44±0.22 | 2.47±0.24 | 2.17±0.24 | 1.90±0.20 | 2.32±0.36 | 2.58±0.36 | 2.33±0.32 | 1.91±0.12 | | 2.11±0.13 | 2.21±0.14 |  |
| Saha et al., 2018 | Late Holocene | mH1a | HAMTAH-1410 | 32.2678 | 77.3589 | 4125 | 2.5 | 2.7 | 0 | 0.948 | 0.60±0.10 | 07KNSTD |  | 0.14±0.03 | Ar. Mean: 0.26±0.13 ka | 0.13±0.03 | 0.19±0.04 | 0.19±0.04 | 0.17±0.03 | 0.13±0.02 | 0.17±0.04 | 0.17±0.04 | 0.17±0.04 | 0.13±0.02 | | 0.16±0.03 | ± |  |
| Saha et al., 2018 | Late Holocene | mH1a | HAMTAH-1502 | 32.2703 | 77.3579 | 3861 | 2 | 2.7 | 0 | 0.923 | 2.57±0.25 | 07KNSTD |  | 0.74±0.09 | Peak: 0.15 ka ! | 0.70±0.07 | 0.92±0.08 | 0.93±0.09 | 0.80±0.09 | 0.65±0.08 | 0.87±0.17 | 0.88±0.17 | 0.89±0.14 | 0.63±0.06 | | 0.78±0.08 | 0.82±0.08 |  |
| Scherler et al. (2010) | Tons Valley | Location E | DS6-32 | 31.0715 | 78.4992 | 4071 | 3 | 2.7 | 0 | 0.95 | 1.21±0.06 | 07KNSTD | 0.26±0.08 | 0.31±0.02 | Chi-squared: ! | 0.29±0.02 | 0.41±0.04 | 0.44±0.04 | 0.36±0.04 | 0.36±0.04 | 0.38±0.06 | 0.37±0.06 | 0.39±0.06 | 0.37±0.02 | | 0.36±0.02 | 0.27±0.01 |  |
|  |  |  |  |  |  |  |  |  |  |  |  |  |  |  | Skewness: ! |  |  |  |  |  |  |  |  |  |  |  |  |  |
|  |  |  |  |  |  |  |  |  |  |  |  |  |  |  | Outlier: 0 |  |  |  |  |  |  |  |  |  |  |  |  |  |
|  |  |  |  |  |  |  |  |  |  |  |  |  |  |  | Wt. mean: 0.28±0.08 ka |  |  |  |  |  |  |  |  |  |  |  |  |  |
|  |  |  |  |  |  |  |  |  |  |  |  |  |  |  | Ar. Mean: 0.26±0.08 ka |  |  |  |  |  |  |  |  |  |  |  |  |  |
| Scherler et al. (2010) | Tons Valley | Location E | DS6-33 | 31.0715 | 78.4977 | 4046 | 2 | 2.7 | 0 | 0.97 | 0.77±0.09 | 07KNSTD |  | 0.18±0.02 | Peak: ! | 0.2±0.03 | 0.26±0.03 | 0.27±0.04 | 0.23±0.03 | 0.23±0.03 | 0.23±0.05 | 0.23±0.05 | 0.24±0.04 | ± | | 0.22±0.03 | 0.17±0.00 |  |
| Owen et al. (2010) | N/A | M10 | Na48 | 30.4633 | 81.2169 | 5508 | 2 | 2.7 | 0 | 0.946 | 2.90±0.41 | 07KNSTD | 0.24±0.15 | 0.39±0.06 | Chi-squared: 10.04 | 0.37±0.06 | 0.53±0.08 | 0.54±0.07 | 0.46±0.06 | 0.34±0.05 | 0.46±0.07 | 0.44±0.06 | 0.47±0.07 | 0.47±0.07 | | 0.46±0.07 | 0.34±0.05 |  |
|  |  |  |  |  |  |  |  |  |  |  |  |  |  |  | Skewness: +0.20 |  |  |  |  |  |  |  |  |  |  |  |  |  |
| Owen et al. (2010) | N/A | M10 | Na49 | 30.4634 | 81.2168 | 5506 | 1 | 2.7 | 0 | 0.946 | 22.99±0.65 | 07KNSTD |  | 3.40±0.21 | Outlier: 2 | 3.24±0.17 | 3.09±0.08 | 3.15±0.09 | 3.07±0.08 | 2.71±0.08 | 2.87±0.06 | 3.08±0.08 | 2.86±0.06 | 3.18±0.09 | | 3.17±0.09 | 2.67±0.08 |  |
| Owen et al. (2010) | N/A | M10 | Na50 | 30.4634 | 81.2169 | 5509 | 1.5 | 2.7 | 0 | 0.946 | 0.75±0.27 | 07KNSTD |  | 0.09±0.04 | Wt. mean: 0.22±0.15 ka | 0.09±0.03 | 0.14±0.05 | 0.14±0.05 | 0.12±0.04 | 0.09±0.03 | 0.12±0.04 | 0.11±0.04 | 0.12±0.04 | ± | | 0.12±0.00 | 0.09±0.03 |  |
| Owen et al. (2010) | N/A | M10 | Na52 | 30.464 | 81.217 | 5514 | 1.5 | 2.7 | 0 | 0.952 | 28.09±0.69 | 07KNSTD |  | 4.09±0.24 | Ar. Mean: 0.24±0.15 ka | 3.87±0.18 | 3.75±0.10 | 3.83±0.10 | 3.65±0.08 | 3.30±0.08 | 3.39±0.08 | 3.70±0.09 | 3.36±0.08 | 3.92±0.10 | | 3.90±0.10 | 3.24±0.08 |  |
| Owen et al. (2010) | N/A | M10 | Na53 | 30.4638 | 81.2159 | 5509 | 2 | 2.7 | 0 | 0.955 | 1.78±0.12 | 07KNSTD |  | 0.23±0.02 | Peak: ! | 0.22±0.02 | 0.32±0.02 | 0.33±0.02 | 0.29±0.02 | 0.21±0.01 | 0.28±0.02 | 0.26±0.02 | 0.28±0.02 | ± | | ± | 0.21±0.01 |  |
| Srivastava (2012; Sci. report) | Bhujbas glacial stage | N/A | MILAP1 | 30.9395 | 79.0635 | 3908 | 5 | 2.7 | 0 | 0.947 | 0.71±0.05 | 07KNSTD | 0.21±0.02 | 0.21±0.02 | ! | 0.19±0.02 | 0.27±0.03 | 0.27±0.03 | 0.24±0.03 | 0.18±0.02 | 0.25±0.04 | 0.24±0.04 | 0.25±0.04 | ± | | 0.23±0.00 | 0.18±0.01 |  |
| Murari et al. (2014) | mbd1 | N/A | KAL18 | 30.7565 | 78.9629 | 4132 | 2 | 2.7 | 0 | 0.949 | 0.86±0.12 | 07KNSTD | 0.21±0.02 | 0.2±0.03 | Chi-squared: 0.61 | 0.22±0.03 | 0.29±0.05 | 0.29±0.05 | 0.25±0.05 | 0.25±0.05 | 0.26±0.06 | 0.25±0.06 | 0.26±0.06 | ± | | 0.25±0.04 | 0.19±0.00 |  |
|  |  |  |  |  |  |  |  |  |  |  |  |  |  |  | Skewness: +1.73 |  |  |  |  |  |  |  |  |  |  |  |  |  |
|  |  |  |  |  |  |  |  |  |  |  |  |  |  |  | Outlier: 1 |  |  |  |  |  |  |  |  |  |  |  |  |  |
| Murari et al. (2014) | mbd1 | N/A | KAL19 | 30.7585 | 78.9627 | 4182 | 2 | 2.7 | 0 | 0.937 | 1.07±0.08 | 07KNSTD |  | 0.23±0.02 | Wt. mean: 0.22±0.02 ka | 0.26±0.03 | 0.35±0.04 | 0.36±0.04 | 0.31±0.04 | 0.31±0.04 | 0.32±0.06 | 0.31±0.05 | 0.33±0.05 | ± | | 0.31±0.02 | 0.23±0.00 |  |
| Murari et al. (2014) | mbd1 | N/A | KAL20 | 30.7567 | 78.9628 | 4115 | 2 | 2.7 | 0 | 0.94 | 3.64±0.30 | 07KNSTD |  | 0.9±0.09 | Ar. Mean: 0.21±0.02 ka | 0.96±0.1 | 1.11±0.08 | 1.12±0.09 | 1.00±0.08 | 1.00±0.08 | 1.05±0.13 | 1.08±0.15 | 1.06±0.12 | 1.00±0.08 | | 0.97±0.08 | 0.81±0.08 |  |
| Murari et al. (2014) | mbd1 | N/A | KAL21 | 30.7567 | 78.9653 | 4108 | 2 | 2.7 | 0 | 0.939 | 0.86±0.12 | 07KNSTD |  | 0.2±0.03 | Peak: 0.22 ka | 0.22±0.03 | 0.30±0.05 | 0.30±0.05 | 0.26±0.05 | 0.26±0.05 | 0.27±0.06 | 0.26±0.06 | 0.27±0.06 | ± | | 0.26±0.04 | 0.19±0.00 |  |
| Murari et al. (2014) | mbd2 | N/A | KAL1 | 30.7778 | 78.9515 | 3641 | 1.5 | 2.7 | 0 | 0.937 | 0.99±0.08 | 07KNSTD | 0.16±0.15 | 0.3±0.03 | Chi-squared: 103.54 | 0.32±0.03 | 0.43±0.05 | 0.44±0.05 | 0.37±0.05 | 0.37±0.05 | 0.40±0.07 | 0.39±0.07 | 0.41±0.07 | 0.39±0.03 | | 0.37±0.03 | 0.28±0.03 |  |
|  |  |  |  |  |  |  |  |  |  |  |  |  |  |  | Skewness: +0.58 |  |  |  |  |  |  |  |  |  |  |  |  |  |
| Murari et al. (2014) | mbd2 | N/A | KAL2 | 30.7777 | 78.9512 | 3650 | 2 | 2.7 | 0 | 0.923 | 1.14±0.12 | 07KNSTD |  | 0.35±0.04 | Outlier: 0 | 0.36±0.05 | 0.50±0.06 | 0.51±0.05 | 0.43±0.06 | 0.43±0.06 | 0.46±0.09 | 0.45±0.08 | 0.47±0.08 | 0.45±0.05 | | 0.42±0.04 | 0.33±0.05 |  |
| Murari et al. (2014) | mbd2 | N/A | KAL3 | 30.7782 | 78.9506 | 3646 | 1.5 | 2.7 | 0 | 0.935 | 0.25±0.03 | 07KNSTD |  | 0.07±0.01 | Wt. mean: 0.07±0.15 ka | 0.08±0.01 | 0.11±0.01 | 0.11±0.01 | 0.09±0.01 | 0.09±0.01 | 0.10±0.02 | 0.10±0.02 | 0.10±0.02 | 0.10±0.01 | | 0.09±0.01 | 0.07±0.01 |  |
| Murari et al. (2014) | mbd2 | N/A | KAL4 | 30.7782 | 78.9504 | 3657 | 1.5 | 2.7 | 0 | 0.923 | 0.05±0.02 | 07KNSTD |  | 0.02±0.01 | Ar. Mean: 0.16±0.15 ka | 0.02±0.01 | 0.02±0.01 | 0.02±0.01 | 0.02±0.01 | 0.02±0.01 | 0.02±0.01 | 0.02±0.01 | 0.02±0.01 | 0.02±0.01 | | 0.02±0.01 | 0.02±0.01 |  |
| Murari et al. (2014) | mbd2 | N/A | KAL5 | 30.7782 | 78.9533 | 3644 | 5 | 2.7 | 0 | 0.929 | 0.24±0.03 | 07KNSTD |  | 0.07±0.01 | Peak: ! | 0.08±0.02 | 0.11±0.01 | 0.11±0.01 | 0.09±0.01 | 0.09±0.01 | 0.10±0.02 | 0.09±0.02 | 0.10±0.02 | 0.10±0.01 | | 0.09±0.01 | 0.07±0.01 |  |
| Murari et al. (2014) | mbd3 | N/A | KAL10 | 30.7839 | 78.9511 | 3579 | 3 | 2.7 | 0 | 0.937 | 0.62±0.12 | 07KNSTD | 0.15±0.10 | 0.19±0.04 | Chi-squared: 71.63 | 0.21±0.05 | 0.28±0.07 | 0.28±0.07 | 0.24±0.07 | 0.24±0.07 | 0.26±0.07 | 0.25±0.07 | 0.26±0.07 | ± | | 0.24±0.05 | 0.18±0.00 |  |
|  |  |  |  |  |  |  |  |  |  |  |  |  |  |  | Skewness: -1.57 |  |  |  |  |  |  |  |  |  |  |  |  |  |
| Murari et al. (2014) | mbd3 | N/A | KAL11 | 30.7838 | 78.9509 | 3576 | 2.5 | 2.7 | 0 | 0.937 | 0.70±0.09 | 07KNSTD |  | 0.22±0.03 | Outlier: 2 | 0.24±0.03 | 0.32±0.05 | 0.32±0.05 | 0.28±0.05 | 0.28±0.05 | 0.29±0.06 | 0.28±0.06 | 0.30±0.06 | ± | | 0.27±0.03 | 0.21±0.00 |  |
| Murari et al. (2014) | mbd3 | N/A | KAL12 | 30.7836 | 78.9507 | 3568 | 2 | 2.7 | 0 | 0.937 | 4.73±0.25 | 07KNSTD |  | 1.59±0.12 | Wt. mean: 0.06±0.10 ka | 1.72±0.14 | 1.79±0.18 | 1.81±0.17 | 1.58±0.18 | 1.58±0.18 | 1.72±0.26 | 1.90±0.34 | 1.73±0.25 | 1.60±0.08 | | 1.51±0.08 | 1.39±0.08 |  |
| Murari et al. (2014) | mbd3 | N/A | KAL13 | 30.7834 | 78.9504 | 3571 | 3 | 2.7 | 0 | 0.943 | 6.93±0.31 | 07KNSTD |  | 2.38±0.17 | Ar. Mean: 0.15±0.10 ka | 2.61±0.21 | 2.60±0.20 | 2.60±0.20 | 2.34±0.20 | 2.34±0.20 | 2.51±0.36 | 2.74±0.34 | 2.52±0.31 | 2.51±0.11 | | 2.36±0.11 | 2.04±0.11 |  |
| Murari et al. (2014) | mbd3 | N/A | KAL14 | 30.7831 | 78.9501 | 3571 | 2.5 | 2.7 | 0 | 0.938 | 0.10±0.01 | 07KNSTD |  | 0.03±0.01 | Peak: ! | 0.03±0.01 | 0.05±0.01 | 0.05±0.01 | 0.04±0.01 | 0.04±0.01 | 0.04±0.01 | 0.04±0.01 | 0.04±0.01 | 0.04±0.01 | | 0.04±0.00 | 0.03±0.01 |  |
| Murari et al. (2014) | mbd4 | N/A | KAL7 | 30.7898 | 78.9522 | 3635 | 3 | 2.7 | 0 | 0.911 | 0.68±0.12 | 07KNSTD | 0.13±0.11 | 0.21±0.04 | Chi-squared: ! | 0.23±0.04 | 0.31±0.04 | 0.31±0.05 | 0.27±0.04 | 0.27±0.04 | 0.28±0.07 | 0.27±0.07 | 0.29±0.07 | ± | | 0.26±0.00 | 0.20±0.04 |  |
|  |  |  |  |  |  |  |  |  |  |  |  |  |  |  | Skewness: ! |  |  |  |  |  |  |  |  |  |  |  |  |  |
|  |  |  |  |  |  |  |  |  |  |  |  |  |  |  | Outlier: 0 |  |  |  |  |  |  |  |  |  |  |  |  |  |
|  |  |  |  |  |  |  |  |  |  |  |  |  |  |  | Wt. mean: 0.06±0.11 ka |  |  |  |  |  |  |  |  |  |  |  |  |  |
|  |  |  |  |  |  |  |  |  |  |  |  |  |  |  | Ar. Mean: 0.13±0.11 ka |  |  |  |  |  |  |  |  |  |  |  |  |  |
| Murari et al. (2014) | mbd4 | N/A | KAL9 | 30.7897 | 78.9523 | 3637 | 3 | 2.7 | 0 | 0.911 | 0.17±0.03 | 07KNSTD |  | 0.05±0.01 | Peak: ! | 0.06±0.02 | 0.08±0.02 | 0.08±0.02 | 0.07±0.02 | 0.07±0.02 | 0.07±0.02 | 0.07±0.02 | 0.07±0.02 | 0.07±0.01 | | 0.06±0.01 | 0.05±0.01 |  |
| This study | MP1 | Late Holocene | PAR1 | 34.0835 | 75.9998 | 3700 | 2 | 2.7 | 0 | 0.968 | 0.56±0.06 | 07KNSTD | 0.16±0.05 | 0.15±0.02 | Chi-squared: 5.44 | 0.14±0.02 | 0.20±0.03 | 0.20±0.03 | 0.18±0.03 | 0.14±0.02 | 0.19±0.04 | 0.18±0.04 | 0.19±0.03 | 0.19±0.02 | | 0.18±0.02 | 0.14±0.02 |  |
|  |  |  |  |  |  |  |  |  |  |  |  |  |  |  | Skewness: -0.86 |  |  |  |  |  |  |  |  |  |  |  |  |  |
|  |  |  |  |  |  |  |  |  |  |  |  |  |  |  | Outlier: 0 |  |  |  |  |  |  |  |  |  |  |  |  |  |
| This study | MP1 | Late Holocene | PAR2 | 34.0844 | 76.0001 | 3687 | 3 | 2.7 | 0 | 0.973 | 0.33±0.08 | 07KNSTD |  | 0.09±0.02 | Wt. mean: 0.15x±0.05 ka | 0.08±0.02 | 0.12±0.03 | 0.12±0.03 | 0.11±0.03 | 0.08±0.02 | 0.11±0.03 | 0.10±0.03 | 0.11±0.03 | 0.11±0.02 | | 0.10±0.02 | 0.08±0.02 |  |
| This study | MP1 | Late Holocene | PAR3 | 34.0846 | 76.0002 | 3678 | 3.5 | 2.7 | 0 | 0.974 | 0.71±0.05 | 07KNSTD |  | 0.19±0.02 | Ar. Mean: 0.16±0.05 ka | 0.19±0.02 | 0.26±0.03 | 0.26±0.03 | 0.24±0.03 | 0.18±0.02 | 0.24±0.04 | 0.23±0.04 | 0.24±0.04 | 0.25±0.01 | | 0.23±0.01 | 0.18±0.01 |  |
| This study | MP1 | Late Holocene | PAR6 | 34.085 | 76.0004 | 3667 | 2 | 2.7 | 0 | 0.969 | 0.74±0.12 | 07KNSTD |  | 0.21±0.04 | Peak: 0.17 ka | 0.19±0.04 | 0.27±0.04 | 0.27±0.04 | 0.24±0.04 | 0.18±0.03 | 0.25±0.06 | 0.24±0.06 | 0.25±0.06 | 0.26±0.03 | | 0.24±0.03 | 0.18±0.03 |  |
| ***Climatic Zone 2b: Transitional climatic region—central and eastern Himalaya*** | | | | | | | | | | | | | | | | | | | | | | | | | | | | |
| Schaefer et al. (2008) | Fu Qu valley, Puluo moraine sequence | Puluo 2 moraine | Ny-12-1 | 28.168 | 85.933 | 4261 | 2.5 | 2.7 | 0 | 0.978 | 66.54±2.93 | S555 | 13.47±0.58 | 13.42±0.75 | Chi-squared: 0.41 | 14.07±0.88 | 15.30±1.50 | 15.50±1.60 | 14.20±1.50 | 14.20±1.50 | 14.60±2.30 | 15.20±2.20 | 14.50±2.10 | 13.50±0.60 | | 13.32±0.59 | 12.86±0.60 |  |
|  |  |  |  |  |  |  |  |  |  |  |  |  |  |  | Skewness: +1.30 |  |  |  |  |  |  |  |  |  |  |  |  |  |
| Schaefer et al. (2008) | Fu Qu valley, Puluo moraine sequence | Puluo 2 moraine | Ny-12-2 | 28.168 | 85.933 | 4261 | 2.5 | 2.7 | 0 | 0.978 | 65.58±2.62 | S555 |  | 13.23±0.72 | Outlier: 1 | 13.91±0.85 | 14.30±1.20 | 14.40±1.20 | 13.10±1.20 | 13.10±1.20 | 13.50±2.10 | 14.00±2.00 | 13.40±1.90 | 13.32±0.54 | | 13.15±0.53 | 12.67±0.54 |  |
| Schaefer et al. (2008) | Fu Qu valley, Puluo moraine sequence | Puluo 2 moraine | Ny-13 | 28.168 | 85.933 | 4273 | 4 | 2.7 | 0 | 0.978 | 70.95±5.11 | S555 |  | 14.29±1.07 | Wt. mean: 13.34±0.58 ka | 14.97±1.19 | 14.40±1.20 | 14.60±1.20 | 13.30±1.20 | 13.30±1.20 | 13.70±2.10 | 14.00±2.00 | 13.60±1.90 | 14.22±1.03 | | 14.07±1.02 | 13.80±1.03 |  |
| Schaefer et al. (2008) | Fu Qu valley, Puluo moraine sequence | Puluo 2 moraine | Ny-14-1 | 28.168 | 85.933 | 4265 | 6.1 | 2.7 | 0 | 0.978 | 47.58±2.62 | S555 |  | 10.19±0.66 | Ar. Mean: 13.47±0.58 ka | 10.8±0.65 | 29.10±2.30 | 29.50±2.50 | 27.90±2.30 | 27.90±2.30 | 28.20±3.90 | 28.50±3.80 | 27.90±3.50 | 10.82±0.60 | | 10.52±0.58 | 9.44±0.60 |  |
| Schaefer et al. (2008) | Fu Qu valley, Puluo moraine sequence | Puluo 2 moraine | Ny-14-2 | 28.168 | 85.933 | 4265 | 6.1 | 2.7 | 0 | 0.978 | 62.00±2.48 | S555 |  | 12.93±0.75 | Peak: 13.27 ka | 13.59±0.8 | 20.00±1.40 | 20.20±1.50 | 18.90±1.40 | 18.90±1.40 | 19.20±2.60 | 19.70±2.30 | 19.10±2.30 | 13.05±0.52 | | 12.91±0.52 | 12.32±0.52 |  |
| Finkel et al. (2003) | Chhukung glacial stage | N/A | E79 | 27.92 | 86.81 | 4624 | 5 | 2.7 | 0 | 0.974 | 67.90±1.70 | LLNL3000 | 11.52±0.11 | 11.40±0.51 | Chi-squared: 0.04 | 11.93±0.72 | 12.12±0.94 | 12.00±1.00 | 11.20±0.94 | 11.20±0.94 | 11.30±1.80 | 12.00±1.70 | 11.20±1.60 | 11.69±0.29 | | 11.63±0.29 | 10.79±0.29 |  |
|  |  |  |  |  |  |  |  |  |  |  |  |  |  |  | Skewness: -1.15 |  |  |  |  |  |  |  |  |  |  |  |  |  |
|  |  |  |  |  |  |  |  |  |  |  |  |  |  |  | Outlier: 0 |  |  |  |  |  |  |  |  |  |  |  |  |  |
|  |  |  |  |  |  |  |  |  |  |  |  |  |  |  | Wt. mean: 11.50±0.11 ka |  |  |  |  |  |  |  |  |  |  |  |  |  |
| Finkel et al. (2003) | Chhukung glacial stage | N/A | E80 | 27.92 | 86.81 | 4624 | 5 | 2.7 | 0 | 0.974 | 69.00±3.10 | LLNL3000 |  | 11.55±0.66 | Ar. Mean: 11.52±0.11 ka | 12.13±0.83 | 12.30±1.10 | 12.50±1.20 | 11.40±1.10 | 11.40±1.10 | 11.50±1.90 | 12.20±1.80 | 11.40±1.70 | 11.85±0.53 | | 11.78±0.53 | 10.97±0.53 |  |
| Finkel et al. (2003) | Chhukung glacial stage | N/A | E81 | 27.92 | 86.81 | 4628 | 5 | 2.7 | 0 | 0.974 | 69.50±2.70 | LLNL3000 |  | 11.61±0.64 | Peak: 11.49 ka | 12.21±0.8 | 12.40±1.10 | 12.60±1.20 | 11.00±1.10 | 11.00±1.10 | 11.50±1.80 | 12.20±1.80 | 11.50±1.70 | 11.91±0.46 | | 11.83±0.46 | 11.02±0.46 |  |
| Finkel et al. (2003) | Chhukung glacial stage | N/A | E29 | 27.9 | 86.87 | 4755 | 5 | 2.7 | 0 | 0.986 | 133.80±1.60 | LLNL3000 | 10.97±0.03 | 19.77±0.82 | Chi-squared: ! | 20.38±0.91 | 20.50±1.10 | 20.80±1.30 | 19.60±1.10 | 19.60±1.10 | 19.50±2.30 | 20.00±2.10 | 19.40±2.10 | 19.50±0.23 | | 19.39±0.23 | 19.83±0.23 |  |
|  |  |  |  |  |  |  |  |  |  |  |  |  |  |  | Skewness: ! |  |  |  |  |  |  |  |  |  |  |  |  |  |
|  |  |  |  |  |  |  |  |  |  |  |  |  |  |  | Outlier: 1 |  |  |  |  |  |  |  |  |  |  |  |  |  |
|  |  |  |  |  |  |  |  |  |  |  |  |  |  |  | Wt. mean: 10.97±0.03 ka |  |  |  |  |  |  |  |  |  |  |  |  |  |
| Finkel et al. (2003) | Chhukung glacial stage | N/A | E30 | 27.9 | 86.87 | 4837 | 5 | 2.7 | 0 | 0.986 | 72.00±2.20 | LLNL3000 |  | 10.99±0.49 | Ar. Mean: 10.97±0.03 ka | 11.37±0.57 | 11.50±0.97 | 12.00±1.00 | 10.63±0.97 | 10.63±0.97 | 10.60±1.80 | 11.30±1.70 | 10.50±1.60 | 11.30±0.35 | | 11.27±0.35 | 10.26±0.35 |  |
| Finkel et al. (2003) | Chhukung glacial stage | N/A | E31 | 27.9 | 86.87 | 4812 | 5 | 2.7 | 0 | 0.986 | 70.80±1.60 | LLNL3000 |  | 10.95±0.46 | Peak: 10.96 ka | 11.31±0.53 | 11.44±0.96 | 12.00±1.00 | 10.57±0.96 | 10.57±0.96 | 10.50±1.80 | 11.30±1.70 | 10.50±1.60 | 11.27±0.26 | | 11.23±0.25 | 10.20±0.26 |  |
| Owen et al. (2005) | Local LGM | Local LGM moraines | GS17 | 29.6122 | 102.1035 | 1858 | 5 | 2.7 | 0 | 0.948 | 14.59±0.80 | LLNL3000 | 10.10±0.73 | 11.25±0.7 | Chi-squared: 0.89 | 12.65±0.95 | 12.70±1.30 | 12.50±1.30 | 11.10±1.30 | 11.10±1.30 | 12.80±2.10 | 13.10±2.10 | 12.70±1.90 | 12.34±0.68 | | 11.32±0.62 | 10.75±0.68 |  |
|  |  |  |  |  |  |  |  |  |  |  |  |  |  |  | Skewness: +1.03 |  |  |  |  |  |  |  |  |  |  |  |  |  |
| Owen et al. (2005) | Local LGM | Local LGM moraines | GS19 | 29.6118 | 102.1048 | 1864 | 5 | 2.7 | 0 | 0.968 | 13.52±0.90 | LLNL3000 |  | 10.3±0.75 | Outlier: 0 | 11.42±0.88 | 11.40±1.30 | 11.30±1.30 | 10.00±1.30 | 10.00±1.30 | 12.00±2.00 | 11.90±1.90 | 11.50±1.80 | 11.32±0.76 | | 10.44±0.70 | 9.72±0.76 |  |
| Owen et al. (2005) | Local LGM | Local LGM moraines | GS20 | 29.6118 | 102.1051 | 1884 | 5 | 2.7 | 0 | 0.965 | 12.86±1.10 | LLNL3000 |  | 9.79±0.94 | Wt. mean: 10.28±0.73 ka | 10.93±0.93 | 10.70±1.50 | 10.60±1.50 | 9.40±1.50 | 9.40±1.50 | 10.90±2.10 | 11.00±2.00 | 10.80±1.90 | 10.90±0.94 | | 9.96±0.85 | 9.15±0.94 |  |
| Owen et al. (2005) | Local LGM | Local LGM moraines | GS19R | 29.6118 | 102.1048 | 1864 | 5 | 2.7 | 0 | 0.968 | 12.85±1.02 | LLNL3000 |  | 9.87±0.89 | Ar. Mean: 10.10±0.73 ka | 11.02±0.9 | 10.90±1.40 | 10.80±1.40 | 9.50±1.40 | 9.50±1.40 | 11.00±2.10 | 11.00±2.00 | 10.90±1.90 | 10.98±0.87 | | 10.03±0.80 | 9.23±0.87 |  |
| Owen et al. (2005) | Local LGM | Local LGM moraines | GS20R | 29.6115 | 102.1051 | 1884 | 5 | 2.7 | 0 | 0.965 | 12.27±1.18 | LLNL3000 |  | 9.31±1 | Peak: 10.12 ka | 10.49±0.99 | 10.20±1.50 | 10.10±1.50 | 8.90±1.50 | 8.90±1.50 | 10.30±2.10 | 11.00±2.00 | 10.30±1.90 | 10.42±1.00 | | 9.53±0.92 | 8.73±1.00 |  |
| Owen et al. (2005) | N/A | "Recessional moraine" | GS14 | 29.57 | 102.02 | 1670 | 5 | 2.7 | 0 | 1 | 8.80±0.66 | LLNL3000 | 6.03±1.97 | 7.42±0.6 | Chi-squared: ! | 8.42±0.82 | 8.08±0.97 | 7.98±0.99 | 7.12±0.97 | 7.12±0.97 | 8.20±1.50 | 8.60±1.50 | 8.20±1.40 | 8.43±0.63 | | 7.51±0.56 | 6.97±0.63 |  |
|  |  |  |  |  |  |  |  |  |  |  |  |  |  |  | Skewness: ! |  |  |  |  |  |  |  |  |  |  |  |  |  |
|  |  |  |  |  |  |  |  |  |  |  |  |  |  |  | Outlier: 0 |  |  |  |  |  |  |  |  |  |  |  |  |  |
|  |  |  |  |  |  |  |  |  |  |  |  |  |  |  | Wt. mean: 6.22±1.97 ka |  |  |  |  |  |  |  |  |  |  |  |  |  |
|  |  |  |  |  |  |  |  |  |  |  |  |  |  |  | Ar. Mean: 6.03±1.97 ka |  |  |  |  |  |  |  |  |  |  |  |  |  |
| Owen et al. (2005) | N/A | "Recessional moraine" | GS15 | 29.59 | 102.02 | 1670 | 5 | 2.7 | 0 | 1 | 5.13±0.79 | LLNL3000 |  | 4.64±0.69 | Peak: ! | 5.28±0.73 | 4.90±0.85 | 4.85±0.89 | 4.43±0.85 | 4.43±0.85 | 5.00±1.00 | 5.00±1.00 | 4.92±0.99 | 5.27±0.81 | | 4.70±0.73 | 4.06±0.81 |  |
| Finkel et al. (2003) | Thuklha glacial stage | N/A | E61 | 27.91 | 86.89 | 5005 | 5 | 2.7 | 0 | 0.979 | 29.70±2.20 | LLNL3000 | 4.43±0.32 | 4.73±0.37 | Chi-squared: 1.08 | 5.04±0.39 | 4.53±0.53 | 4.61±0.55 | 4.36±0.53 | 4.36±0.53 | 4.12±0.78 | 4.55±0.84 | 4.10±0.71 | 4.91±0.36 | | 4.85±0.36 | 3.95±0.36 |  |
|  |  |  |  |  |  |  |  |  |  |  |  |  |  |  | Skewness: -0.55 |  |  |  |  |  |  |  |  |  |  |  |  |  |
|  |  |  |  |  |  |  |  |  |  |  |  |  |  |  | Outlier: 0 |  |  |  |  |  |  |  |  |  |  |  |  |  |
|  |  |  |  |  |  |  |  |  |  |  |  |  |  |  | Wt. mean: 4.39±0.32 ka |  |  |  |  |  |  |  |  |  |  |  |  |  |
| Finkel et al. (2003) | Thuklha glacial stage | N/A | E62 | 27.91 | 86.88 | 4955 | 5 | 2.7 | 0 | 0.979 | 27.40±1.10 | LLNL3000 |  | 4.47±0.25 | Ar. Mean: 4.43±0.32 ka | 4.78±0.29 | 4.26±0.41 | 4.35±0.44 | 4.14±0.41 | 4.14±0.41 | 3.88±0.67 | 4.29±0.76 | 3.90±0.60 | 4.61±0.19 | | 4.55±0.18 | 3.72±0.19 |  |
| Finkel et al. (2003) | Thuklha glacial stage | N/A | E63 | 27.91 | 86.88 | 5032 | 5 | 2.7 | 0 | 0.979 | 25.70±1.50 | LLNL3000 |  | 4.09±0.28 | Peak: 4.42 ka | 4.39±0.33 | 3.82±0.41 | 3.90±0.45 | 3.82±0.41 | 3.82±0.41 | 3.49±0.59 | 3.83±0.68 | 3.48±0.53 | 4.19±0.25 | | 4.17±0.24 | 3.37±0.25 |  |
| Owen et al. (2009) | N/A | T5c | Ron-25 | 28.1423 | 86.8536 | 5242 | 2.5 | 2.7 | 0 | 0.98 | 20.00±1.00 | KNSTD | 3.18±0.23 | 3.02±0.2 | Chi-squared: 1.18 | 3.22±0.24 | 2.84±0.18 | 2.88±0.21 | 2.89±0.18 | 2.89±0.18 | 2.63±0.35 | 2.90±0.30 | 2.63±0.31 | 3.05±0.15 | | 3.06±0.15 | 2.44±0.15 |  |
| Owen et al. (2009) | N/A | T5c | Ron-26 | 28.1424 | 86.8535 | 5240 | 3.5 | 2.7 | 0 | 0.98 | 22.10±0.70 | KNSTD |  | 3.35±0.19 | Skewness: +0.38 | 3.58±0.22 | 3.07±0.26 | 3.13±0.27 | 3.17±0.26 | 3.17±0.26 | 2.86±0.33 | 3.07±0.47 | 2.86±0.29 | 3.38±0.11 | | 3.39±0.11 | 2.72±0.11 |  |
| Owen et al. (2009) | N/A | T5c | Ron-27 | 28.1427 | 86.854 | 5247 | 5 | 2.7 | 0 | 0.98 | 8.00±0.60 | KNSTD |  | 1.16±0.1 | Outlier: 4 | 1.22±0.12 | 1.28±0.12 | 1.30±0.12 | 1.22±0.12 | 1.22±0.12 | 1.17±0.16 | 1.20±0.18 | 1.19±0.14 | 1.17±0.09 | | 1.18±0.09 | 0.99±0.09 |  |
| Owen et al. (2009) | N/A | T5c | Ron-28 | 28.1431 | 86.8544 | 5244 | 3 | 2.7 | 0 | 0.98 | 23.00±1.00 | KNSTD |  | 3.47±0.21 | Wt. mean: 3.19±0.23 ka | 3.69±0.24 | 3.17±0.29 | 3.24±0.31 | 3.27±0.29 | 3.27±0.29 | 2.93±0.32 | 3.20±0.50 | 2.93±0.28 | 3.52±0.15 | | 3.54±0.15 | 2.81±0.15 |  |
| Owen et al. (2009) | N/A | T5c | Ron-29 | 28.1432 | 86.8539 | 5240 | 1 | 2.7 | 0 | 0.98 | 19.70±1.10 | KNSTD |  | 2.93±0.22 | Ar. Mean: 3.18±0.23 ka | 3.13±0.24 | 2.79±0.21 | 2.80±0.20 | 2.83±0.21 | 2.83±0.21 | 2.58±0.35 | 2.81±0.35 | 2.58±0.31 | 2.98±0.17 | | 3.00±0.17 | 2.37±0.17 |  |
| Owen et al. (2009) | N/A | T5c | Ron-30 | 28.1436 | 86.854 | 5245 | 3 | 2.7 | 0 | 0.98 | 20.60±1.00 | KNSTD |  | 3.12±0.2 | Peak: 3.14 ka | 3.32±0.23 | 2.90±0.18 | 2.94±0.19 | 2.97±0.18 | 2.97±0.18 | 2.70±0.36 | 2.91±0.29 | 2.70±0.31 | 3.13±0.15 | | 3.14±0.15 | 2.52±0.15 |  |
| Owen et al. (2009) | N/A | T5c | Ron-31 | 28.1432 | 86.8523 | 5193 | 4.5 | 2.7 | 0 | 0.95 | 8.40±0.60 | KNSTD |  | 1.29±0.11 |  | 1.36±0.13 | 1.38±0.13 | 1.40±0.12 | 1.32±0.13 | 1.32±0.13 | 1.27±0.19 | 1.32±0.23 | 1.29±0.16 | 1.28±0.09 | | 1.28±0.09 | 1.10±0.09 |  |
| Owen et al. (2009) | N/A | T5c | Ron-32 | 28.1432 | 86.8523 | 5190 | 0.5 | 2.7 | 0 | 0.95 | 8.10±0.80 | KNSTD |  | 1.2±0.14 |  | 1.27±0.15 | 1.31±0.13 | 1.33±0.15 | 1.25±0.13 | 1.25±0.13 | 1.21±0.18 | 1.24±0.23 | 1.22±0.17 | 1.20±0.12 | | 1.20±0.12 | 1.02±0.12 |  |
| Owen et al. (2009) | N/A | T5c | Ron-33 | 28.1428 | 86.8523 | 5195 | 1 | 2.7 | 0 | 0.96 | 9.40±0.50 | KNSTD |  | 1.38±0.1 |  | 1.46±0.12 | 1.47±0.13 | 1.50±0.13 | 1.41±0.13 | 1.41±0.13 | 1.35±0.18 | 1.43±0.24 | 1.36±0.17 | 1.38±0.07 | | 1.37±0.07 | 1.18±0.07 |  |
| Owen et al. (2009) | N/A | T6 | Ron-61 | 28.1363 | 86.8515 | 5175 | 1.5 | 2.7 | 0 | 0.98 | 14.30±0.50 | KNSTD | 2.08±0.09 | 2.14±0.14 | Chi-squared: 0.51 | 2.29±0.17 | 2.17±0.21 | 2.20±0.20 | 2.11±0.21 | 2.11±0.21 | 1.96±0.32 | 2.19±0.36 | 1.96±0.29 | 2.16±0.08 | | 2.14±0.08 | 1.78±0.08 |  |
|  |  |  |  |  |  |  |  |  |  |  |  |  |  |  | Skewness: -1.49 |  |  |  |  |  |  |  |  |  |  |  |  |  |
|  |  |  |  |  |  |  |  |  |  |  |  |  |  |  | Outlier: 0 |  |  |  |  |  |  |  |  |  |  |  |  |  |
|  |  |  |  |  |  |  |  |  |  |  |  |  |  |  | Wt. mean: 2.06±0.09 ka |  |  |  |  |  |  |  |  |  |  |  |  |  |
| Owen et al. (2009) | N/A | T6 | Ron-62 | 28.1364 | 86.8515 | 5178 | 5 | 2.7 | 0 | 0.98 | 12.90±0.40 | KNSTD |  | 1.98±0.11 | Ar. Mean: 2.08±0.09 ka | 2.11±0.15 | 2.00±0.18 | 2.04±0.21 | 1.93±0.18 | 1.93±0.18 | 1.81±0.28 | 2.01±0.32 | 1.82±0.25 | 1.97±0.06 | | 1.94±0.06 | 1.65±0.06 |  |
| Owen et al. (2009) | N/A | T6 | Ron-63 | 28.1364 | 86.8515 | 5174 | 2.5 | 2.7 | 0 | 0.98 | 13.90±0.70 | KNSTD |  | 2.11±0.16 | Peak:2.05 ka | 2.25±0.19 | 2.12±0.21 | 2.17±0.24 | 2.06±0.21 | 2.06±0.21 | 1.90±0.30 | 2.14±0.36 | 1.92±0.29 | 2.10±0.11 | | 2.08±0.11 | 1.74±0.11 |  |
| Owen et al. (2005) | Neoglacial | Neoglacial moraines | GS1 | 29.5699 | 101.9855 | 3174 | 5 | 2.7 | 0 | 0.924 | 3.11±0.39 | LLNL3000 | 1.04±0.10 | 1.17±0.16 | Chi-squared: 0.31 | 1.29±0.18 | 1.34±0.16 | 1.35±0.17 | 1.24±0.16 | 1.24±0.16 | 1.32±0.21 | 1.34±0.25 | 1.30±0.20 | 1.25±0.16 | | 1.18±0.15 | 1.05±0.16 |  |
| Owen et al. (2005) | Neoglacial | Neoglacial moraines | GS2 | 29.5699 | 101.9855 | 3160 | 5 | 2.7 | 0 | 0.939 | 4.79±0.61 | LLNL3000 |  | 1.84±0.25 | Skewness: +0.72 | 2.03±0.3 | 1.90±0.30 | 1.90±0.30 | 1.83±0.30 | 1.83±0.30 | 1.88±0.37 | 2.04±0.48 | 1.89±0.35 | 1.94±0.25 | | 1.81±0.23 | 1.60±0.25 |  |
| Owen et al. (2005) | Neoglacial | Neoglacial moraines | GS3 | 29.5758 | 102.0037 | 3056 | 5 | 2.7 | 0 | 0.942 | 2.46±0.32 | LLNL3000 |  | 0.98±0.14 | Outlier: 2 | 1.07±0.16 | 1.17±0.16 | 1.18±0.17 | 1.06±0.16 | 1.06±0.16 | 1.10±0.20 | 1.10±0.20 | 1.16±0.19 | 1.06±0.14 | | 1.02±0.13 | 0.87±0.14 |  |
| Owen et al. (2005) | Neoglacial | Neoglacial moraines | GS4 | 29.5758 | 102.0037 | 3056 | 5 | 2.7 | 0 | 0.942 | 8.20±1.40 | LLNL3000 |  | 3.39±0.58 | Wt. mean: 1.06±0.10 ka | 3.77±0.64 | 3.29±0.67 | 3.31±0.71 | 3.29±0.67 | 3.29±0.67 | 3.24±0.81 | 3.48±0.87 | 3.24±0.77 | 3.73±0.64 | | 3.51±0.60 | 2.89±0.64 |  |
| Owen et al. (2005) | Neoglacial | Neoglacial moraines | GS8 | 29.5768 | 102.0031 | 2972 | 5 | 2.7 | 0 | 0.976 | 2.14±1.01 | LLNL3000 |  | 0.95±0.44 | Ar. Mean: 1.04±0.10 ka | 1.01±0.47 | 1.07±0.42 | 1.07±0.42 | 0.95±0.42 | 0.95±0.42 | 1.04±0.42 | 1.04±0.44 | 1.06±0.42 | 1.0±0.51 | | 1.03±0.49 | 0.89±0.42 |  |
| Owen et al. (2005) | Neoglacial | Neoglacial moraines | GS9 | 29.5769 | 102.0031 | 2959 | 5 | 2.7 | 0 | 1 | 2.71±0.29 | LLNL3000 |  | 1.07±0.13 | Peak: 1.06 ka | 1.18±0.15 | 1.26±0.14 | 1.26±0.15 | 1.14±0.14 | 1.14±0.14 | 1.23±0.19 | 1.24±0.22 | 1.25±0.18 | 1.15±0.12 | | 1.09±0.12 | 0.95±0.12 |  |
| Owen et al. (2005) | Little Ice Age | Little Ice Age moraines | GS5 | 29.5713 | 101.999 | 3010 | 5 | 2.7 | 0 | 0.936 | 1.03±0.30 | LLNL3000 | 0.46±0.06 | 0.41±0.12 | Chi-squared: ! | 0.45±0.14 | 0.59±0.19 | 0.60±0.17 | 0.49±0.19 | 0.49±0.19 | 0.56±0.22 | 0.54±0.21 | 0.58±0.22 | ± | | 0.49±0.00 | 0.37±0.11 |  |
|  |  |  |  |  |  |  |  |  |  |  |  |  |  |  | Skewness: ! |  |  |  |  |  |  |  |  |  |  |  |  |  |
|  |  |  |  |  |  |  |  |  |  |  |  |  |  |  | Outlier: 1 |  |  |  |  |  |  |  |  |  |  |  |  |  |
|  |  |  |  |  |  |  |  |  |  |  |  |  |  |  | Wt. mean: 0.48±0.06 ka |  |  |  |  |  |  |  |  |  |  |  |  |  |
| Owen et al. (2005) | Little Ice Age | Little Ice Age moraines | GS6 | 29.5717 | 101.9989 | 2988 | 5 | 2.7 | 0 | 0.932 | 7.84±2.95 | LLNL3000 |  | 3.41±1.25 | Ar. Mean: 0.46±0.06 ka | 3.8±1.37 | 3.30±1.50 | 3.30±1.50 | 3.30±1.50 | 3.30±1.50 | 3.30±1.50 | 3.50±1.70 | 3.30±1.50 | 3.69±1.39 | | 3.46±1.39 | 2.86±1.08 |  |
| Owen et al. (2005) | Little Ice Age | Little Ice Age moraines | GS7 | 29.5703 | 101.9989 | 2979 | 5 | 2.7 | 0 | 0.927 | 1.21±0.13 | LLNL3000 |  | 0.5±0.06 | Peak: 0.49 ka | 0.54±0.07 | 0.72±0.09 | 0.72±0.09 | 0.59±0.09 | 0.59±0.09 | 0.68±0.14 | 0.65±0.13 | 0.71±0.11 | 0.62±0.07 | | 0.59±0.06 | 0.45±0.07 |  |
| Owen et al. (2009) | N/A | T7 | Ron-51 | 28.1298 | 86.8531 | 5216 | 3.5 | 2.7 | 0 | 0.97 | 1.10±0.80 | KNSTD | 0.33±0.19 | 0.18±0.13 | Chi-squared: 12.31 | 0.16±0.11 | 0.21±0.17 | 0.22±0.18 | 0.19±0.17 | 0.19±0.17 | 0.18±0.15 | 0.18±0.15 | 0.19±0.15 | 0.2±0.16 | | ± | 0.15±0.11 |  |
|  |  |  |  |  |  |  |  |  |  |  |  |  |  |  | Skewness: +1.32 |  |  |  |  |  |  |  |  |  |  |  |  |  |
|  |  |  |  |  |  |  |  |  |  |  |  |  |  |  | Outlier: 0 |  |  |  |  |  |  |  |  |  |  |  |  |  |
|  |  |  |  |  |  |  |  |  |  |  |  |  |  |  | Wt. mean: 0.34±0.19 ka |  |  |  |  |  |  |  |  |  |  |  |  |  |
| Owen et al. (2009) | N/A | T7 | Ron-53 | 28.1294 | 86.8542 | 5213 | 2.3 | 2.7 | 0 | 0.97 | 2.00±0.20 | KNSTD |  | 0.27±0.03 | Ar. Mean: 0.33±0.19 ka | 0.28±0.03 | 0.38±0.06 | 0.39±0.06 | 0.35±0.06 | 0.35±0.06 | 0.33±0.07 | 0.32±0.06 | 0.34±0.06 | 0.35±0.04 | | 0.00±0.00 | 0.25±0.04 |  |
| Owen et al. (2009) | N/A | T7 | Ron-55 | 28.1296 | 86.856 | 5225 | 2 | 2.7 | 0 | 0.97 | 3.90±0.30 | KNSTD |  | 0.55±0.05 | Peak: ! | 0.58±0.06 | 0.74±0.07 | 0.75±0.07 | 0.65±0.07 | 0.65±0.07 | 0.66±0.12 | 0.62±0.11 | 0.68±0.12 | 0.65±0.05 | | 0.66±0.05 | 0.48±0.05 |  |
| ***Climatic Zone 3: Wet-temperate climatic region—central and eastern Himalaya*** | | | | | | | | | | | | | | | | | | | | | | | | | | | | |
| Kong et al. (2009b) | No stage name given | Ganhaizi | YN-62 | 27.1217 | 100.2547 | 3070 | 2 | 2.7 | 0 | 1 | 32.70±2.00 | NIST_Certified | 12.97±1.41 | 13.97±0.96 | Chi-squared: ! | 15.12±1.11 | 15.30±1.30 | 15.30±1.40 | 14.00±1.30 | 14.00±1.30 | 15.00±2.20 | 15.50±2.30 | 15.00±2.00 | 14.54±0.89 | | 13.87±0.85 | 13.49±0.89 |  |
|  |  |  |  |  |  |  |  |  |  |  |  |  |  |  | Skewness: ! |  |  |  |  |  |  |  |  |  |  |  |  |  |
|  |  |  |  |  |  |  |  |  |  |  |  |  |  |  | Outlier: 0 |  |  |  |  |  |  |  |  |  |  |  |  |  |
|  |  |  |  |  |  |  |  |  |  |  |  |  |  |  | Wt. mean: 12.97±1.41 ka |  |  |  |  |  |  |  |  |  |  |  |  |  |
|  |  |  |  |  |  |  |  |  |  |  |  |  |  |  | Ar. Mean: 12.97±1.41 ka |  |  |  |  |  |  |  |  |  |  |  |  |  |
| Kong et al. (2009b) | No stage name given | Ganhaizi | YN-63 | 27.1218 | 100.2543 | 3060 | 2 | 2.7 | 0 | 1 | 27.60±2.00 | NIST_Certified |  | 11.97±0.96 | Peak: ! | 13.13±1.1 | 13.10±1.40 | 13.20±1.50 | 11.90±1.40 | 11.90±1.40 | 12.80±2.20 | 13.40±2.20 | 13.00±2.00 | 12.78±0.93 | | 12.13±0.88 | 11.44±0.93 |  |
| Kong et al. (2009b) | No stage name given | Ganheba | YN-64 | 27.0523 | 100.2555 | 2930 | 2 | 2.7 | 0 | 1 | 22.10±0.80 | NIST_Certified | 11.96±1.94 | 10.58±0.53 | Chi-squared: ! | 11.45±0.64 | 11.00±1.00 | 11.40±1.10 | 10.26±1.00 | 10.26±1.00 | 11.10±1.90 | 11.70±1.80 | 11.20±1.70 | 11.31±0.41 | | 10.88±0.40 | 9.88±0.41 |  |
|  |  |  |  |  |  |  |  |  |  |  |  |  |  |  | Skewness: ! |  |  |  |  |  |  |  |  |  |  |  |  |  |
|  |  |  |  |  |  |  |  |  |  |  |  |  |  |  | Outlier: 0 |  |  |  |  |  |  |  |  |  |  |  |  |  |
|  |  |  |  |  |  |  |  |  |  |  |  |  |  |  | Wt. mean: 11.62±1.94 ka |  |  |  |  |  |  |  |  |  |  |  |  |  |
|  |  |  |  |  |  |  |  |  |  |  |  |  |  |  | Ar. Mean: 11.96±1.94 ka |  |  |  |  |  |  |  |  |  |  |  |  |  |
| Kong et al. (2009b) | No stage name given | Ganheba | YN-65 | 27.053 | 100.2543 | 2960 | 2 | 2.7 | 0 | 1 | 29.10±1.00 | NIST_Certified |  | 13.33±0.68 | Peak: ! | 14.55±0.81 | 14.60±1.10 | 14.70±1.20 | 13.30±1.10 | 13.30±1.10 | 14.30±2.10 | 15.00±2.00 | 14.40±1.90 | 13.92±0.48 | | 13.30±0.46 | 12.80±0.48 |  |
| Kong et al. (2009b) | No stage name given | N/A | YN-48 | 27.0383 | 100.1137 | 2440 | 2 | 2.7 | 0 | 0.99 | 2.36±0.24 | NIST_Certified | ! | 1.64±0.2 | ! | 1.89±0.23 | 1.75±0.21 | 1.75±0.21 | 1.60±0.21 | 1.60±0.21 | 1.70±0.30 | 1.86±0.35 | 1.75±0.28 | 1.79±0.18 | | 1.66±0.17 | 1.43±0.18 |  |
| Kong et al. (2009b) | No stage name given | N/A | YN-50 | 27.038 | 100.0973 | 2210 | 2 | 2.7 | 0 | 0.99 | 30.20±0.60 | NIST_Certified |  | 20.93±0.87 |  | 22.78±1.14 | 23.10±1.70 | 23.00±1.80 | 20.80±1.70 | 20.80±1.70 | 23.20±3.30 | 23.40±3.30 | 23.00±3.00 | 21.96±0.44 | | 20.50±0.41 | 21.15±0.44 |  |
| Pratt-Sitaula (2005) | Younger Dryas | Yak Glacier lower | Be-425 | 28.6856 | 84.1772 | 4581 | 1.5 | 2.65 | 0 | 0.971 | 95.62±2.10 | LLNL3000 | 11.66±1.29 | 15.19±0.68 | Chi-squared: ! | 15.78±0.84 | 16.40±1.30 | 16.60±1.30 | 15.20±1.30 | 15.20±1.30 | 15.40±2.10 | 16.00±2.30 | 15.30±1.90 | 14.99±0.33 | | 14.97±0.33 | 14.84±0.33 |  |
|  |  |  |  |  |  |  |  |  |  |  |  |  |  |  | Skewness: ! |  |  |  |  |  |  |  |  |  |  |  |  |  |
|  |  |  |  |  |  |  |  |  |  |  |  |  |  |  | Outlier: 1 |  |  |  |  |  |  |  |  |  |  |  |  |  |
|  |  |  |  |  |  |  |  |  |  |  |  |  |  |  | Wt. mean: 11.39±1.29 ka |  |  |  |  |  |  |  |  |  |  |  |  |  |
| Pratt-Sitaula (2005) | Younger Dryas | Yak Glacier lower | Be-426 | 28.6846 | 84.1777 | 4530 | 1.5 | 2.65 | 0 | 0.971 | 75.23±2.00 | LLNL3000 |  | 12.57±0.65 | Ar. Mean: 11.66±1.29 ka | 13.11±0.72 | 13.50±1.10 | 13.70±1.20 | 12.00±1.10 | 12.00±1.10 | 12.60±1.90 | 13.00±2.00 | 12.60±1.70 | 12.74±0.34 | | 12.61±0.34 | 11.95±0.34 |  |
| Pratt-Sitaula (2005) | Younger Dryas | Yak Glacier lower | Be-427 | 28.6838 | 84.1788 | 4496 | 1.5 | 2.65 | 0 | 0.971 | 62.10±1.58 | LLNL3000 |  | 10.75±0.48 | Peak: ! | 11.18±0.51 | 11.39±0.98 | 12.00±1.00 | 10.36±0.98 | 10.36±0.98 | 10.60±1.80 | 11.40±1.70 | 10.60±1.60 | 11.17±0.29 | | 11.04±0.28 | 10.02±0.29 |  |
| Pratt-Sitaula (2005) | Early Holocene | Lyapche Glacier | Be-218 | 28.6987 | 84.2545 | 3815 | 4 | 2.7 | 0 | 0.931 | 41.83±1.18 | LLNL3000 | 11.54±0.80 | 10.75±0.49 | Chi-squared: 2.02 | 11.32±0.56 | 11.59±0.96 | 12.00±1.00 | 10.39±0.96 | 10.39±0.96 | 11.10±1.80 | 11.80±1.70 | 11.10±1.60 | 11.27±0.32 | | 11.01±0.31 | 10.07±0.32 |  |
|  |  |  |  |  |  |  |  |  |  |  |  |  |  |  | Skewness: +0.02 |  |  |  |  |  |  |  |  |  |  |  |  |  |
|  |  |  |  |  |  |  |  |  |  |  |  |  |  |  | Outlier: 1 |  |  |  |  |  |  |  |  |  |  |  |  |  |
| Pratt-Sitaula (2005) | Early Holocene | Lyapche Glacier | Be-219 | 28.6987 | 84.2545 | 3815 | 4 | 2.7 | 0 | 0.931 | 27.48±0.80 | LLNL3000 |  | 7.14±0.34 | Wt. mean: 11.38±0.80 ka | 7.61±0.43 | 7.47±0.59 | 7.55±0.63 | 6.76±0.59 | 6.76±0.59 | 7.20±1.10 | 7.80±1.10 | 7.16±0.99 | 7.63±0.22 | | 7.32±0.21 | 6.61±0.22 |  |
| Pratt-Sitaula (2005) | Early Holocene | Lyapche Glacier | Be-220 | 28.6987 | 84.2545 | 3815 | 3 | 2.7 | 0 | 0.94 | 46.65±1.31 | LLNL3000 |  | 11.54±0.56 | Ar. Mean: 11.54±0.80 ka | 12.35±0.75 | 13.00±1.00 | 12.80±1.10 | 11.42±1.00 | 11.42±1.00 | 12.10±1.80 | 12.80±1.90 | 12.10±1.60 | 12.04±0.34 | | 11.71±0.33 | 11.03±0.34 |  |
| Pratt-Sitaula (2005) | Early Holocene | Lyapche Glacier | Be-221 | 28.6987 | 84.2545 | 3815 | 3 | 2.7 | 0 | 0.94 | 49.85±1.35 | LLNL3000 |  | 12.34±0.67 | Peak: 11.20 ka | 13.12±0.73 | 13.50±1.10 | 13.60±1.20 | 12.18±1.10 | 12.18±1.10 | 13.00±2.00 | 13.60±1.90 | 12.90±1.80 | 12.75±0.35 | | 12.39±0.34 | 11.79±0.35 |  |
| Abramowski (2004) | LT3: Langtang Stage | N/A | LT32 | 28.2 | 85.62 | 3853 | 2.5 | 2.7 | 0 | 0.968 | 43.10±1.70 | S555 | 10.90±0.43 | 10.96±0.55 | Chi-squared: 0.55 | 11.55±0.68 | 12.00±1.00 | 11.90±1.10 | 11.00±1.00 | 11.00±1.00 | 11.30±1.80 | 12.00±1.70 | 11.30±1.70 | 11.43±0.45 | | 11.20±0.44 | 10.30±0.45 |  |
|  |  |  |  |  |  |  |  |  |  |  |  |  |  |  | Skewness: -0.59 |  |  |  |  |  |  |  |  |  |  |  |  |  |
|  |  |  |  |  |  |  |  |  |  |  |  |  |  |  | Outlier: 1 |  |  |  |  |  |  |  |  |  |  |  |  |  |
| Abramowski (2004) | LT3: Langtang Stage | N/A | LT33 | 28.2 | 85.62 | 3851 | 2.5 | 2.7 | 0 | 0.957 | 40.30±1.60 | S555 |  | 10.45±0.55 | Wt. mean: 10.87±0.43 ka | 11.1±0.59 | 11.00±1.00 | 11.30±1.10 | 10.06±1.00 | 10.06±1.00 | 10.70±1.80 | 11.40±1.70 | 10.70±1.60 | 11.08±0.44 | | 10.79±0.43 | 9.75±0.44 |  |
| Abramowski (2004) | LT3: Langtang Stage | N/A | LT35 | 28.2 | 85.62 | 3846 | 1 | 2.7 | 0 | 0.978 | 45.80±2.10 | S555 |  | 11.3±0.62 | Ar. Mean: 10.90±0.43 ka | 12.03±0.83 | 12.30±1.10 | 12.40±1.20 | 11.10±1.10 | 11.10±1.10 | 11.80±1.90 | 12.40±1.80 | 11.80±1.70 | 11.78±0.54 | | 11.50±0.53 | 10.74±0.54 |  |
| Abramowski (2004) | LT3: Langtang Stage | N/A | LT36 | 28.2 | 85.62 | 3846 | 2 | 2.7 | 0 | 0.92 | 19.20±0.80 | S555 |  | 5.5±0.28 | Peak: 10.87 ka | 5.9±0.33 | 5.51±0.36 | 5.56±0.39 | 5.16±0.36 | 5.16±0.36 | 5.30±0.70 | 5.66±0.72 | 5.31±0.62 | 5.88±0.25 | | 5.65±0.24 | 4.82±0.25 |  |
| Pratt-Sitaula (2011) | Early-mid Holocene | Syaktan Glacier | Be-226 | 28.6882 | 84.0234 | 4500 | 3 | 2.65 | 0 | 0.969 | 52.35±1.63 | LLNL3000 | 9.48±0.91 | 9.25±0.58 | Chi-squared: 3.38 | 9.86±0.59 | 9.62±0.93 | 10.00±1.00 | 8.72±0.93 | 8.72±0.93 | 9.00±1.50 | 9.70±1.60 | 8.90±1.40 | 9.96±0.31 | | 9.73±0.30 | 8.55±0.31 |  |
|  |  |  |  |  |  |  |  |  |  |  |  |  |  |  | Skewness: +0.97 |  |  |  |  |  |  |  |  |  |  |  |  |  |
|  |  |  |  |  |  |  |  |  |  |  |  |  |  |  | Outlier: 0 |  |  |  |  |  |  |  |  |  |  |  |  |  |
| Pratt-Sitaula (2011) | Early-mid Holocene | Syaktan Glacier | Be-227 | 28.6882 | 84.0234 | 4500 | 3 | 2.65 | 0 | 0.969 | 61.14±1.66 | LLNL3000 |  | 10.72±0.49 | Wt. mean: 9.51±0.91 ka | 11.16±0.52 | 11.35±0.97 | 12.00±1.00 | 10.32±0.97 | 10.32±0.97 | 10.60±1.70 | 11.30±1.70 | 10.50±1.60 | 11.15±0.30 | | 11.02±0.30 | 9.99±0.30 |  |
| Pratt-Sitaula (2011) | Early-mid Holocene | Syaktan Glacier | Be-228 | 28.6944 | 84.0253 | 4744 | 3 | 2.65 | 0 | 0.962 | 58.76±1.65 | LLNL3000 |  | 9.39±0.56 | Ar. Mean: 9.48±0.91 ka | 9.91±0.56 | 9.68±0.93 | 9.86±0.99 | 8.81±0.93 | 8.81±0.93 | 8.90±1.50 | 9.70±1.60 | 8.90±1.40 | 10.00±0.28 | | 9.84±0.28 | 8.63±0.28 |  |
| Pratt-Sitaula (2011) | Early-mid Holocene | Syaktan Glacier | Be-229 | 28.6944 | 84.0253 | 4744 | 3 | 2.65 | 0 | 0.962 | 54.11±1.59 | LLNL3000 |  | 8.54±0.5 | Peak: 9.08 ka | 9.07±0.65 | 8.83±0.83 | 9.00±0.89 | 8.10±0.83 | 8.10±0.83 | 8.20±1.30 | 8.90±1.40 | 8.10±1.20 | 9.23±0.27 | | 8.96±0.26 | 7.94±0.27 |  |
| Pratt-Sitaula (2011) | Early-mid Holocene | Danfe Glacier | Be-433 | 28.4735 | 84.3172 | 4009 | 1.5 | 2.75 | 0 | 0.917 | 37.42±0.96 | LLNL3000 | 8.87±0.36 | 8.73±0.52 | Chi-squared: 0.53 | 9.48±0.63 | 9.21±0.86 | 9.35±0.92 | 8.28±0.86 | 8.28±0.86 | 8.70±1.50 | 9.50±1.50 | 8.70±1.30 | 9.59±0.25 | | 9.16±0.24 | 8.14±0.25 |  |
| Pratt-Sitaula (2011) | Early-mid Holocene | Danfe Glacier | Be-434 | 28.4739 | 84.3165 | 4057 | 1 | 2.75 | 0 | 0.945 | 37.99±0.98 | LLNL3000 |  | 8.33±0.44 | Skewness: -0.35 | 8.98±0.63 | 8.80±0.80 | 8.90±0.88 | 7.92±0.80 | 7.92±0.80 | 8.30±1.40 | 9.00±1.40 | 8.30±1.20 | 9.11±0.24 | | 8.65±0.22 | 7.79±0.24 |  |
| Pratt-Sitaula (2011) | Early-mid Holocene | Danfe Glacier | Be-435 | 28.4741 | 84.3163 | 4068 | 2 | 2.75 | 0 | 0.945 | 40.08±1.03 | LLNL3000 |  | 8.85±0.53 | Outlier: 0 | 9.59±0.6 | 9.33±0.86 | 9.47±0.94 | 8.39±0.86 | 8.39±0.86 | 8.80±1.50 | 9.60±1.50 | 8.80±1.30 | 9.71±0.25 | | 9.31±0.24 | 8.25±0.25 |  |
| Pratt-Sitaula (2011) | Early-mid Holocene | Danfe Glacier | Be-437 | 28.4734 | 84.3178 | 3978 | 2 | 2.75 | 0 | 0.916 | 38.65±0.99 | LLNL3000 |  | 9.28±0.55 | Wt. mean: 8.82±0.36 ka | 9.99±0.56 | 9.78±0.92 | 9.92±0.97 | 8.76±0.92 | 8.76±0.92 | 9.30±1.60 | 10.00±1.60 | 9.30±1.40 | 10.03±0.26 | | 9.71±0.25 | 8.59±0.26 |  |
| Pratt-Sitaula (2011) | Early-mid Holocene | Danfe Glacier | Be-438 | 28.4784 | 84.3126 | 4213 | 1 | 2.75 | 0 | 0.962 | 45.56±1.17 | LLNL3000 |  | 9.19±0.55 | Ar. Mean: 8.87±0.36 ka | 9.87±0.56 | 9.60±0.90 | 9.78±0.98 | 8.67±0.90 | 8.67±0.90 | 9.10±1.60 | 9.80±1.60 | 9.00±1.40 | 9.95±0.26 | | 9.65±0.25 | 8.50±0.26 |  |
| Pratt-Sitaula (2011) | Early-mid Holocene | Danfe Glacier | Be-439 | 28.4784 | 84.3126 | 4215 | 1 | 2.75 | 0 | 0.962 | 42.92±0.94 | LLNL3000 |  | 8.57±0.47 | Peak: 8.82 ka | 9.25±0.63 | 9.00±0.80 | 9.14±0.89 | 8.13±0.80 | 8.13±0.80 | 8.50±1.40 | 9.20±1.50 | 8.50±1.20 | 9.38±0.21 | | 8.97±0.20 | 8.00±0.21 |  |
| Pratt-Sitaula (2011) | Early-mid Holocene | Danfe Glacier | Be-440 | 28.4789 | 84.3116 | 4249 | 2 | 2.75 | 0 | 0.98 | 46.68±1.20 | LLNL3000 |  | 9.17±0.54 |  | 9.83±0.57 | 9.57±0.89 | 9.73±0.98 | 8.63±0.89 | 8.63±0.89 | 9.00±1.50 | 9.80±1.60 | 9.00±1.40 | 9.92±0.26 | | 9.63±0.25 | 8.47±0.26 |  |
| Pratt-Sitaula (2005) | Early-mid Holocene | Yak upper | Be-420 | 28.6915 | 84.1773 | 4821 | 1 | 2.65 | 0 | 0.977 | 60.89±1.57 | LLNL3000 | 8.72±0.40 | 9.05±0.55 | Chi-squared: 0.75 | 9.6±0.6 | 9.33±0.86 | 9.51±0.94 | 8.52±0.86 | 8.52±0.86 | 8.60±1.40 | 9.30±1.50 | 8.50±1.30 | 9.75±0.25 | | 9.57±0.25 | 8.36±0.25 |  |
|  |  |  |  |  |  |  |  |  |  |  |  |  |  |  | Skewness: -1.14 |  |  |  |  |  |  |  |  |  |  |  |  |  |
| Pratt-Sitaula (2005) | Early-mid Holocene | Yak upper | Be-421 | 28.6908 | 84.1778 | 4798 | 1 | 2.65 | 0 | 0.977 | 60.40±1.55 | LLNL3000 |  | 9.07±0.55 | Outlier: 0 | 9.63±0.6 | 9.36±0.87 | 9.54±0.95 | 8.54±0.87 | 8.54±0.87 | 8.60±1.50 | 9.40±1.50 | 8.60±1.30 | 9.77±0.25 | | 9.58±0.25 | 8.38±0.25 |  |
| Pratt-Sitaula (2005) | Early-mid Holocene | Yak upper | Be-422 | 28.6903 | 84.1786 | 4770 | 1 | 2.65 | 0 | 0.977 | 57.58±1.48 | LLNL3000 |  | 8.7±0.52 | Wt. mean: 8.64±0.40 ka | 9.25±0.64 | 9.01±0.82 | 9.18±0.92 | 8.23±0.82 | 8.23±0.82 | 8.30±1.40 | 9.10±1.40 | 8.30±1.20 | 9.42±0.24 | | 9.20±0.24 | 8.09±0.24 |  |
| Pratt-Sitaula (2005) | Early-mid Holocene | Yak upper | Be-423 | 28.6894 | 84.18 | 4702 | 2 | 2.65 | 0 | 0.977 | 55.33±1.43 | LLNL3000 |  | 8.69±0.52 | Ar. Mean: 8.72±0.40 ka | 9.28±0.64 | 9.01±0.82 | 9.18±0.92 | 8.22±0.82 | 8.22±0.82 | 8.30±1.40 | 9.10±1.40 | 8.30±1.20 | 9.43±0.24 | | 9.19±0.24 | 8.09±0.24 |  |
| Pratt-Sitaula (2005) | Early-mid Holocene | Yak upper | Be-424 | 28.6894 | 84.18 | 4702 | 1 | 2.65 | 0 | 0.977 | 52.00±1.31 | LLNL3000 |  | 8.09±0.42 | Peak: 8.74 ka | 8.53±0.56 | 8.35±0.71 | 8.50±0.82 | 7.67±0.71 | 7.67±0.71 | 7.80±1.20 | 8.50±1.30 | 7.70±1.10 | 8.65±0.22 | | 8.42±0.21 | 7.54±0.22 |  |
| Gayer et al. (2006) | N/A | M3 | GA24 | 28.232 | 85.188 | 4490 | 3 | 2.7 | 0 | 0.99 | 31.78±3.24 | NIST_27900 | 7.04±0.64 | 6.58±0.62 | Chi-squared: ! | 6.96±0.65 | 6.62±0.87 | 6.73±0.92 | 6.21±0.87 | 6.21±0.87 | 6.20±1.20 | 6.80±1.40 | 6.20±1.10 | 7.01±0.72 | | 6.82±0.70 | 5.96±0.72 |  |
|  |  |  |  |  |  |  |  |  |  |  |  |  |  |  | Skewness: ! |  |  |  |  |  |  |  |  |  |  |  |  |  |
|  |  |  |  |  |  |  |  |  |  |  |  |  |  |  | Outlier: 0 |  |  |  |  |  |  |  |  |  |  |  |  |  |
|  |  |  |  |  |  |  |  |  |  |  |  |  |  |  | Wt. mean: 7.08±0.64 ka |  |  |  |  |  |  |  |  |  |  |  |  |  |
|  |  |  |  |  |  |  |  |  |  |  |  |  |  |  | Ar. Mean: 7.04±0.64 ka |  |  |  |  |  |  |  |  |  |  |  |  |  |
| Gayer et al. (2006) | N/A | M3 | GA54 | 28.227 | 85.188 | 4434 | 3 | 2.7 | 0 | 0.98 | 35.74±2.41 | NIST_27900 |  | 7.49±0.56 | Peak: 7.17 ka | 7.96±0.65 | 7.71±0.77 | 7.83±0.83 | 7.08±0.77 | 7.08±0.77 | 7.30±1.20 | 7.90±1.20 | 7.20±1.10 | 8.02±0.54 | | 7.77±0.53 | 6.95±0.54 |  |
| Zech et al. (2009) | Lete | N/A | LE12 | 28.624 | 83.642 | 2440 | 2 | 2.7 | 0 | 0.966 | 8.97±2.02 | 07KNSTD | 6.36±1.21 | 6.36±1.21 | ! | 5.73±1.13 | 6.10±1.50 | 6.10±1.50 | 5.30±1.30 | 5.50±1.20 | 6.00±1.70 | 6.50±1.90 | 6.00±1.60 | 6.37±1.44 | | 5.84±1.32 | 5.15±1.16 |  |
| Barnard et al. (2006) | Langtang glacial stage | N/A | KTM25 | 28.21 | 85.47 | 3230 | 5 | 2.7 | 0 | 0.85 | 14.00±1.90 | LLNL3000 | 5.47±0.40 | 5.75±0.68 | Chi-squared: ! | 6.28±0.74 | 5.88±0.94 | 6.00±1.00 | 5.45±0.94 | 5.45±0.94 | 5.70±1.10 | 6.20±1.50 | 6.00±1.00 | 6.28±0.85 | | 5.91±0.80 | 5.13±0.85 |  |
|  |  |  |  |  |  |  |  |  |  |  |  |  |  |  | Skewness: ! |  |  |  |  |  |  |  |  |  |  |  |  |  |
|  |  |  |  |  |  |  |  |  |  |  |  |  |  |  | Outlier: 0 |  |  |  |  |  |  |  |  |  |  |  |  |  |
|  |  |  |  |  |  |  |  |  |  |  |  |  |  |  | Wt. mean: 5.31±0.40 ka |  |  |  |  |  |  |  |  |  |  |  |  |  |
|  |  |  |  |  |  |  |  |  |  |  |  |  |  |  | Ar. Mean: 5.47±0.40 ka |  |  |  |  |  |  |  |  |  |  |  |  |  |
| Barnard et al. (2006) | Langtang glacial stage | N/A | KTM26 | 28.21 | 85.47 | 3239 | 5 | 2.7 | 0 | 0.85 | 12.40±0.80 | LLNL3000 |  | 5.19±0.35 | Peak: 5.47 ka | 5.65±0.38 | 5.31±0.45 | 5.34±0.48 | 4.87±0.45 | 4.87±0.45 | 5.18±0.75 | 5.52±0.66 | 5.19±0.68 | 5.60±0.36 | | 5.32±0.34 | 4.52±0.36 |  |
| Abramowski (2004) | N/A | MK4: LIA | MK41 | 28.33 | 84.76 | 3900 | 4 | 2.7 | 0 | 1 | 24.90±2.20 | S555 | 4.99±0.92 | 6.29±0.52 | Chi-squared: 2.83 | 6.71±0.57 | 6.39±0.79 | 6.47±0.83 | 5.94±0.79 | 5.94±0.79 | 6.10±1.10 | 6.70±1.40 | 6.00±1.00 | 6.75±0.60 | | 6.51±0.58 | 5.67±0.60 |  |
|  |  |  |  |  |  |  |  |  |  |  |  |  |  |  | Skewness: +1.35 |  |  |  |  |  |  |  |  |  |  |  |  |  |
| Abramowski (2004) | N/A | MK4: LIA | MK42 | 28.33 | 84.76 | 3900 | 4 | 2.7 | 0 | 1 | 10.10±1.50 | S555 |  | 2.76±0.44 | Outlier: 1 | 3.05±0.49 | 2.79±0.37 | 2.81±0.36 | 2.69±0.37 | 2.69±0.37 | 2.70±0.50 | 2.88±0.43 | 2.68±0.47 | 2.90±0.43 | | 2.83±0.42 | 2.30±0.43 |  |
| Abramowski (2004) | N/A | MK4: LIA | MK43 | 28.33 | 84.76 | 3900 | 4 | 2.7 | 0 | 1 | 18.00±1.40 | S555 |  | 4.79±0.39 | Wt. mean: 5.05±0.92 ka | 5.21±0.41 | 4.80±0.50 | 4.89±0.52 | 4.47±0.50 | 4.47±0.50 | 4.59±0.82 | 5.01±0.74 | 4.60±0.74 | 5.13±0.40 | | 4.89±0.38 | 4.10±0.40 |  |
| Abramowski (2004) | N/A | MK4: LIA | MK44 | 28.33 | 84.76 | 3900 | 4 | 2.7 | 0 | 1 | 15.40±2.40 | S555 |  | 4.14±0.62 | Ar. Mean: 4.99±0.92 ka | 4.55±0.68 | 4.12±0.85 | 4.18±0.84 | 3.90±0.85 | 3.90±0.85 | 3.90±0.94 | 4.30±1.10 | 3.90±0.90 | 4.34±0.68 | | 4.19±0.65 | 3.50±0.68 |  |
| Abramowski (2004) | N/A | MK4: LIA | MK45 | 28.33 | 84.76 | 3900 | 4 | 2.7 | 0 | 1 | 17.80±3.40 | S555 |  | 4.74±0.82 | Peak: ! | 5.17±0.85 | 4.78±0.96 | 5.00±1.00 | 4.43±0.96 | 4.43±0.96 | 4.50±1.20 | 5.00±1.10 | 4.60±1.20 | 5.06±0.97 | | 4.83±0.92 | 4.05±0.97 |  |
| Barnard et al. (2006) | Langtang glacial stage | N/A | KTM19 | 28.21 | 85.51 | 3581 | 5 | 2.7 | 0 | 0.95 | 14.50±0.90 | LLNL3000 | 4.60±0.33 | 4.60±0.33 | Chi-squared: ! | 5.05±0.35 | 4.66±0.49 | 4.70±0.50 | 4.30±0.49 | 4.30±0.49 | 4.46±0.78 | 4.88±0.76 | 4.48±0.71 | 4.92±0.31 | | 4.65±0.29 | 3.93±0.31 |  |
|  |  |  |  |  |  |  |  |  |  |  |  |  |  |  | Skewness: ! |  |  |  |  |  |  |  |  |  |  |  |  |  |
|  |  |  |  |  |  |  |  |  |  |  |  |  |  |  | Outlier: 2 |  |  |  |  |  |  |  |  |  |  |  |  |  |
|  |  |  |  |  |  |  |  |  |  |  |  |  |  |  | Wt. mean: 4.60±0.33 ka |  |  |  |  |  |  |  |  |  |  |  |  |  |
| Barnard et al. (2006) | Langtang glacial stage | N/A | KTM20 | 28.21 | 85.51 | 3507 | 5 | 2.7 | 0 | 0.95 | 2.50±0.70 | LLNL3000 |  | 0.80±0.24 | Ar. Mean: 4.60±0.33 ka | 0.87±0.26 | 1.00±0.21 | 1.01±0.23 | 0.89±0.21 | 0.89±0.21 | 0.96±0.26 | 0.97±0.32 | 0.98±0.25 | 0.92±0.26 | | 0.88±0.25 | 0.70±0.26 |  |
| Barnard et al. (2006) | Langtang glacial stage | N/A | KTM21 | 28.21 | 85.51 | 3510 | 5 | 2.7 | 0 | 0.95 | 3.90±0.70 | LLNL3000 |  | 1.25±0.24 | Peak: ! | 1.38±0.28 | 1.41±0.27 | 1.42±0.28 | 1.29±0.27 | 1.29±0.27 | 1.40±0.30 | 1.44±0.38 | 1.38±0.28 | 1.30±0.23 | | 1.24±0.22 | 1.10±0.23 |  |
| Abramowski (2004) | N/A | LT6 | LT61 | 28.21 | 85.53 | 3523 | 1.5 | 2.7 | 0 | 0.957 | 12.90±0.80 | S555 | 4.42±0.15 | 4.31±0.31 | Chi-squared: ! | 4.76±0.35 | 4.36±0.47 | 4.40±0.49 | 4.05±0.47 | 4.05±0.47 | 4.18±0.75 | 4.59±0.81 | 4.19±0.68 | 4.57±0.28 | | 4.34±0.27 | 3.67±0.28 |  |
|  |  |  |  |  |  |  |  |  |  |  |  |  |  |  | Skewness: ! |  |  |  |  |  |  |  |  |  |  |  |  |  |
|  |  |  |  |  |  |  |  |  |  |  |  |  |  |  | Outlier: 0 |  |  |  |  |  |  |  |  |  |  |  |  |  |
|  |  |  |  |  |  |  |  |  |  |  |  |  |  |  | Wt. mean: 4.42±0.15 ka |  |  |  |  |  |  |  |  |  |  |  |  |  |
|  |  |  |  |  |  |  |  |  |  |  |  |  |  |  | Ar. Mean: 4.42±0.15 ka |  |  |  |  |  |  |  |  |  |  |  |  |  |
| Abramowski (2004) | N/A | LT6 | LT63 | 28.21 | 85.53 | 3525 | 1 | 2.7 | 0 | 0.941 | 13.40±0.70 | S555 |  | 4.52±0.29 | Peak: 4.43 ka | 4.97±0.33 | 4.59±0.45 | 4.63±0.46 | 4.23±0.45 | 4.23±0.45 | 4.40±0.75 | 4.82±0.74 | 4.41±0.67 | 4.82±0.25 | | 4.55±0.24 | 3.85±0.25 |  |
| Zech et al. (2009) | Neoglacial | N/A | DK21 | 28.602 | 84.459 | 3150 | 2 | 2.7 | 0 | 0.911 | 4.86±1.35 | S555 | 1.70±0.50 | 2.05±0.61 | Chi-squared: ! | 2.32±0.7 | 2.26±0.75 | 2.28±0.72 | 2.03±0.75 | 2.03±0.75 | 2.19±0.76 | 2.40±0.73 | 2.22±0.72 | 2.18±0.61 | | 2.00±0.56 | 1.78±0.61 |  |
|  |  |  |  |  |  |  |  |  |  |  |  |  |  |  | Skewness: ! |  |  |  |  |  |  |  |  |  |  |  |  |  |
|  |  |  |  |  |  |  |  |  |  |  |  |  |  |  | Outlier: 0 |  |  |  |  |  |  |  |  |  |  |  |  |  |
|  |  |  |  |  |  |  |  |  |  |  |  |  |  |  | Wt. mean: 1.54±0.50 ka |  |  |  |  |  |  |  |  |  |  |  |  |  |
|  |  |  |  |  |  |  |  |  |  |  |  |  |  |  | Ar. Mean: 1.70±0.50 ka |  |  |  |  |  |  |  |  |  |  |  |  |  |
| Zech et al. (2009) | Neoglacial | N/A | DK22 | 28.602 | 84.459 | 3150 | 2 | 2.7 | 0 | 0.911 | 3.22±0.87 | S555 |  | 1.34±0.38 | Peak: 1.43 ka | 1.48±0.43 | 1.52±0.39 | 1.50±0.40 | 1.37±0.39 | 1.37±0.39 | 1.47±0.44 | 1.57±0.53 | 1.49±0.43 | 1.40±0.38 | | 1.31±0.36 | 1.18±0.38 |  |
| Barnard et al. (2006) | Yala I glacial stage | Yala I | KTM4 | 28.21 | 85.56 | 3924 | 5 | 2.7 | 0 | 0.97 | 2.50±0.80 | LLNL3000 | 0.76±0.20 | 0.62±0.21 | Chi-squared: 0.61 | 0.68±0.23 | 0.83±0.29 | 0.84±0.28 | 0.72±0.29 | 0.72±0.29 | 0.77±0.27 | 0.76±0.30 | 0.80±0.27 | 0.73±0.23 | | 0.72±0.23 | 0.55±0.18 |  |
| Barnard et al. (2006) | Yala I glacial stage | Yala I | KTM5 | 28.21 | 85.56 | 3922 | 5 | 2.7 | 0 | 0.97 | 4.40±1.20 | LLNL3000 |  | 1.12±0.31 | Skewness: +1.21 | 1.22±0.35 | 1.28±0.36 | 1.29±0.31 | 1.18±0.36 | 1.18±0.36 | 1.21±0.35 | 1.26±0.44 | 1.23±0.35 | 1.15±0.31 | | 1.12±0.31 | 0.96±0.26 |  |
| Barnard et al. (2006) | Yala I glacial stage | Yala I | KTM6 | 28.21 | 85.56 | 3923 | 5 | 2.7 | 0 | 0.97 | 2.20±0.80 | LLNL3000 |  | 0.55±0.21 | Outlier: 0 | 0.6±0.22 | 0.74±0.26 | 0.75±0.29 | 0.63±0.26 | 0.63±0.26 | 0.69±0.32 | 0.67±0.31 | 0.72±0.26 | 0.65±0.24 | | 0.63±0.24 | 0.48±0.18 |  |
| Barnard et al. (2006) | Yala I glacial stage | Yala I | KTM7 | 28.21 | 85.57 | 3840 | 5 | 2.7 | 0 | 0.98 | 2.70±0.90 | LLNL3000 |  | 0.7±0.24 | Wt. mean: 0.72±0.20 ka | 0.76±0.26 | 0.92±0.25 | 0.93±0.26 | 0.79±0.25 | 0.79±0.25 | 0.86±0.33 | 0.84±0.35 | 0.88±0.32 | 0.81±0.27 | | 0.79±0.27 | 0.61±0.20 |  |
| Barnard et al. (2006) | Yala I glacial stage | Yala I | KTM8 | 28.21 | 85.57 | 3838 | 5 | 2.7 | 0 | 0.98 | 2.80±0.80 | LLNL3000 |  | 0.72±0.22 | Ar. Mean: 0.76±0.20 ka | 0.79±0.24 | 0.94±0.21 | 0.95±0.22 | 0.82±0.21 | 0.82±0.21 | 0.89±0.30 | 0.88±0.32 | 0.91±0.22 | 0.83±0.24 | | 0.81±0.24 | 0.63±0.18 |  |
| Barnard et al. (2006) | Yala I glacial stage | Yala I | KTM9 | 28.21 | 85.57 | 3839 | 5 | 2.7 | 0 | 0.98 | 3.30±1.00 | LLNL3000 |  | 0.86±0.27 | Peak: 0.68 ka | 0.94±0.29 | 1.05±0.26 | 1.06±0.27 | 0.95±0.26 | 0.95±0.26 | 1.00±0.27 | 1.02±0.31 | 1.02±0.26 | 0.96±0.29 | | 0.93±0.29 | 0.74±0.23 |  |
| Heimsath and McGlynn (2008) | N/A | E moraine crest | NP222 | 28.635 | 84.042 | 4000 | 1.5 | 2.7 | 0 | 0.91 | 4.35±0.65 | LLNL3000 | 0.55±0.16 | 1.1±0.18 | Chi-squared: 5.54 | 1.18±0.2 | 1.30±0.20 | 1.27±0.21 | 1.15±0.20 | 1.15±0.20 | 1.19±0.22 | 1.23±0.29 | 1.21±0.22 | 1.14±0.17 | | 1.11±0.17 | 0.95±0.17 |  |
| Heimsath and McGlynn (2008) | N/A | E moraine crest | NP223 | 28.635 | 84.042 | 3813 | 1.5 | 2.7 | 0 | 0.912 | 2.31±0.36 | LLNL3000 |  | 0.62±0.1 | Skewness: -1.07 | 0.67±0.11 | 0.83±0.16 | 0.84±0.15 | 0.72±0.16 | 0.72±0.16 | 0.78±0.16 | 0.76±0.18 | 0.80±0.16 | 0.74±0.12 | | 0.72±0.11 | 0.56±0.12 |  |
| Heimsath and McGlynn (2008) | N/A | E moraine crest | NP233 | 28.635 | 84.042 | 4275 | 1.5 | 2.7 | 0 | 0.907 | 1.51±0.34 | LLNL3000 |  | 0.31±0.07 | Outlier: 3 | 0.34±0.08 | 0.44±0.11 | 0.45±0.11 | 0.39±0.11 | 0.39±0.11 | 0.40±0.12 | 0.39±0.12 | 0.41±0.12 | 0.40±0.09 | | 0.40±0.09 | 0.29±0.09 |  |
| Heimsath and McGlynn (2008) | N/A | E moraine crest | NP234 | 28.635 | 84.042 | 4216 | 1.5 | 2.7 | 0 | 1 | 2.48±0.20 | LLNL3000 |  | 0.49±0.05 | Wt. mean: 0.55±0.16 ka | 0.53±0.06 | 0.68±0.09 | 0.70±0.08 | 0.58±0.09 | 0.58±0.09 | 0.62±0.12 | 0.60±0.10 | 0.64±0.11 | 0.60±0.05 | | 0.59±0.05 | 0.45±0.05 |  |
| Heimsath and McGlynn (2008) | N/A | E moraine crest | NP235 | 28.635 | 84.042 | 4157 | 1.5 | 2.7 | 0 | 0.91 | 0.74±0.06 | LLNL3000 |  | 0.16±0.02 | Ar. Mean: 0.55±0.16 ka | 0.18±0.02 | 0.23±0.03 | 0.23±0.03 | 0.20±0.03 | 0.20±0.03 | 0.21±0.04 | 0.20±0.03 | 0.21±0.04 | 0.21±0.02 | | 0.20±0.02 | 0.15±0.02 |  |
| Heimsath and McGlynn (2008) | N/A | W moraine crest | NP212 | 28.641 | 84.0438 | 3844 | 1.5 | 2.7 | 0 | 1 | 2.65±0.16 | LLNL3000 |  | 0.64±0.05 | Peak: ! | 0.7±0.06 | 0.85±0.08 | 0.87±0.09 | 0.74±0.08 | 0.74±0.08 | 0.80±0.12 | 0.78±0.13 | 0.82±0.11 | 0.76±0.05 | | 0.74±0.05 | 0.57±0.05 |  |
| Heimsath and McGlynn (2008) | N/A | W moraine crest | NP213 | 28.641 | 84.0438 | 3797 | 1.5 | 2.7 | 0 | 0.935 | 0.70±0.18 | LLNL3000 |  | 0.18±0.04 |  | 0.2±0.05 | 0.25±0.08 | 0.26±0.08 | 0.22±0.08 | 0.22±0.08 | 0.23±0.08 | 0.23±0.08 | 0.24±0.08 | 0.23±0.06 | | 0.22±0.06 | 0.17±0.06 |  |
| Heimsath and McGlynn (2008) | N/A | W moraine crest | NP214 | 28.641 | 84.0438 | 3669 | 1.5 | 2.7 | 0 | 0.943 | 1.98±0.14 | LLNL3000 |  | 0.72±0.07 |  | 0.66±0.06 | 0.77±0.06 | 0.78±0.06 | 0.65±0.06 | 0.52±0.04 | 0.72±0.05 | 0.70±0.05 | 0.74±0.05 | 0.78±0.06 | | 0.75±0.06 | 0.59±0.04 |  |
| a | Reported [Be-10] values have been corrected from background Be-10 detected in procedural blanks | | | |  |  |  |  |  |  |  |  |  |  |  |  |  |  |  |  |  |  |  |  | |  |  |  |
|  | All ages, local and regional glacial stages, weighted [Wt.] and arithmetic [Ar.] means, median, and peaks are reported in thousand years with ± 1σ | | | | | | | | | | | | | | | | | | | | | | | | | | | |
| ! | Undefined peak/age statistics is not possible | | | | | | | | | | | | | | | | | | | | | | | | | | | |
| Note: | Blue colored samples are identified as outliers and removed from further analysis | | | | | | | | | | | | | | | | | | | | | | | | | | | |
| Scaling models: | Major scaling schemes include Lifton-Sato-Dunai (LSD; [8] or SA and SF in [9]), Lal and Stone time-dependent (Lm; [3]), Lal and Stone time-independent (St; [10,11]). | | | | | | | | | | | | | | | | | | | | | | | | | | | |
